# Exploring prognostic factors and treatment strategies for long-term survival in pleomorphic xanthoastrocytoma patients

**DOI:** 10.1038/s41598-024-55202-6

**Published:** 2024-02-26

**Authors:** Chaejin Lee, Yukyeng Byeon, Gung Ju Kim, Juhee Jeon, Chang Ki Hong, Jeong Hoon Kim, Young-Hoon Kim, Young Hyun Cho, Seok Ho Hong, Sang Joon Chong, Sang Woo Song

**Affiliations:** Department of Neurological Surgery, Asan Medical Center, University of Ulsan College of Medicine, 88, Olympic-ro 43-gil, Songpa-gu, Seoul, 05505 Republic of Korea

**Keywords:** Pleomorphic xanthoastrocytoma, Circumscribed glioma, Infiltrative tumor margin, Prognosis, Radiological feature, Cancer therapy, CNS cancer

## Abstract

Pleomorphic xanthoastrocytomas (PXA) are rare, accounting for < 1% of all astrocytomas. Literature on the clinical course and treatment outcomes of PXAs is limited. The study aimed to determine prognosis and treatment strategies for PXAs. Patients who had PXAs surgery between 2000–2021 were retrospectively analyzed for demographics and radiological characteristics. Initial and salvage treatment outcomes were recorded. Overall, 40 and 9 patients had grade 2 and 3 PXAs; their 5-year progression-free survival (PFS) rates were 75.8% and 37.0%, respectively (*p* = 0.003). Univariate analysis revealed that strong T1 enhancement (*p* = 0.036), infiltrative tumor margins (*p* < 0.001), peritumoral edema (*p* = 0.003), WHO grade (*p* = 0.005), and gross total resection (*p* = 0.005) affected the PFS. Multivariate analysis revealed that the WHO grade (*p* = 0.010) and infiltrative tumor margins (*p* = 0.008) influenced the PFS. The WHO grade (*p* = 0.027) and infiltrative tumor margins (*p* = 0.027) also affected the overall survival (OS). Subgroup analysis for grade 2 PXAs revealed no significant associations between adjuvant radiation therapy and the PFS and OS. This study highlighted the heterogeneous nature of PXAs and its impact on patient prognosis. Infiltrative tumor margins emerged as a key prognostic factor. Our findings have emphasized the prognostic relevance of radiological features and the need for larger studies on comprehensive management.

## Introduction

Pleomorphic xanthoastrocytomas (PXAs) are uncommon primary central nervous system tumors, accounting for < 1% of all astrocytomas. They primarily occur in children and young adults and, as per the 2021 World Health Organization (WHO) classification, are categorized as circumscribed gliomas^1^. Despite their rarity and pleomorphic morphological features, PXAs are generally associated with a positive prognosis. They are divided into grade 2 and grade 3, with most falling into grade 2, where complete resection alone often results in a good prognosis. However, PXAs notably present as a distinctive disease entity with a relatively poorer prognosis compared to other conditions classified as circumscribed gliomas (a group commonly associated with more favorable outcomes).

Despite their classification as circumscribed gliomas, PXAs show heterogeneous features on magnetic resonance imaging (MRI)^2,3^. Their relatively typical presentation includes contact with the cortex, presence of a large cystic portion, and diverse enhancement patterns. However, PXAs with a more solid appearance and poorly defined margins are often preoperatively mistaken for high-grade gliomas^3,4^. Whether patient prognosis is affected by such radiological heterogeneity and other clinical factors remains unclear. Thus, we performed the present study to analyze the prognosis of patients with PXAs concerning various clinical factors and radiological characteristics.

Given the rarity of PXAs, we acknowledge the limitations of proposing comprehensive treatment strategies based on the findings of this single-center, retrospective study. Nevertheless, our study has several strengths. First, though single-centered, our study has encompassed a substantial number of cases. Second, we have meticulously analyzed radiological, clinical, and treatment outcome data. Finally, we have diligently documented salvage treatment outcomes upon recurrence. Although a multicenter, prospective study involving larger patient cohorts is warranted in the future, the present study may contribute to developing guidelines for PXA management.

## Methods

### Patient selection and pathological examination

This single-institutional, retrospective study on the clinical outcomes of patients with PXA was approved by the Institutional Review Board of Asan Medical Center on April 3, 2023, with approval number S2023-0703–0001.We screened the institutional database for patients who underwent surgery and were diagnosed with a PXA or anaplastic PXA between January 2001 and January 2021. Only cases with a minimum follow-up period of 12 months and a documented treatment course from the initial diagnosis were included. Overall, 52 patients were identified and their records were reviewed; among these, three patients were excluded due to a follow-up period of < 1 year. Thus, a total of 49 patients were included in this study.

Tumors were classified based on the 2016 WHO criteria. Grade 3 tumors had a mitotic index of ≥ 5/10 high-power fields. The extent of resection (EOR) was determined based on the surgeon’s intraoperative assessment and the postoperative MRI findings: gross total resection (GTR) was considered when there was no evidence of a residual tumor, whereas subtotal resection (STR) was considered when the tumor was majorly but incompletely resected. In the context of the study, STR was defined as achieving a removal of 90% or more of the tumor volume measured from preoperative T1-weighted (T1E) MR images and the volume of residual tumor on postoperative T1E MR images. No patients in this study underwent biopsy alone.

### Clinical and radiological data collection

Data on demographic characteristics, radiological features, and treatment outcomes were retrospectively collected. Demographic data included sex, age, Karnofsky Performance Status (KPS) and symptoms at the initial diagnosis. The tumor volume was manually segmented along the margin of enhancement observed in preoperative contrast-enhanced T1E MR images, with the area calculated using the PACS software (PetaVision for Clinics, 3.1, Asan Medical Center, Seoul, Korea). Similarly, for regions showing abnormal high signal on T2-weighted (T2) MR images, the area was calculated, and the peritumoral edema volume was determined by subtracting the tumor volume. If the volume of peritumoral edema exceeded the tumor volume, it was classified as “evident”; otherwise, it was categorized as “minimal.” The tumor components were categorized as follows: (1) solid tumors without cystic areas within the tumor mass; (2) cystic tumors with cystic features (even solid nodular lesions with predominantly cystic features); and mixed lesions with approximately equal solid and cystic components^2^.

**Figure 1:**
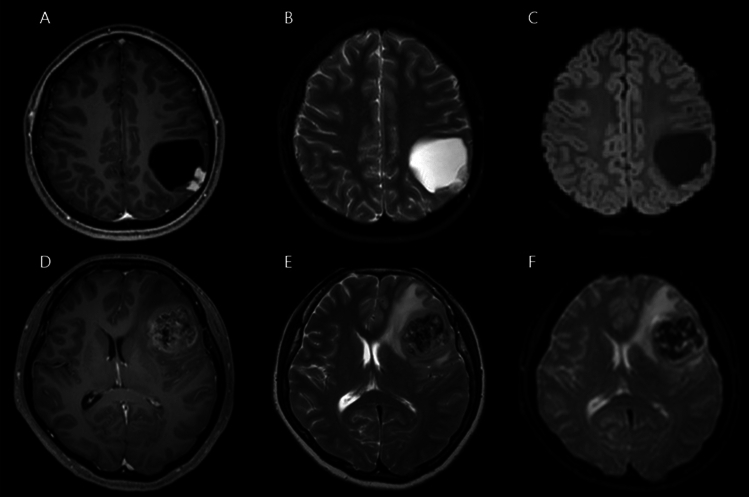
(**a**, **b**, **c**) Images from a case with circumscribed tumor margins. A well-demarcated margin is observed on enhanced T1-weighted image (T1E) and T2-weighted image with an almost absent peritumoral edema. (**d**, **e**, **f**) Images from a case of infiltrative tumor margins. (**d**) A fuzzy, poorly demarcated margin on T1E is observed. (**e**) A heterogeneous T2-high signal is seen in a non-enhancing area (implying mixed components of vasogenic edema and tumor cell infiltration). (**f**) Diffusion restriction with hindered movement of water molecules (due to tumor cell infiltration) is observed.

The methodology for assessing tumor margins involved three neurosurgeons (C.L., Y.B., and S.W.S), who independently evaluated MR images with reference to formal interpretations in neuroradiology. Each neurosurgeon conducted an individual review of the radiologic data, and the final categorization of tumor margins was based on achieving consensus among two or more reviewers. The calculated concordance rate among the three reviewers was 0.670, indicating good agreement. Tumor margins were comprehensively evaluated using T1E images, T2 images, and diffusion-weighted images (DWI) (Fig. 1). Infiltrative tumor margins were characterized by fuzzy, poorly demarcated margins on T1E, coupled with heterogeneous high T2 signals in a non-enhancing area and diffusion restriction. Conversely, circumscribed tumor margins were identified by well-demarcated margins on T1E and homogeneous high T2 signals with relatively low diffusion restriction. This classification was based on previous studies on tumor cell infiltration within non-enhancing peritumoral T2-high lesions (i.e., non-enhancing peritumoral signal abnormality)^5,6^. The quantification of tumor margin nature posed challenges, given that the majority exhibited clear, circumscribed margins. Nevertheless, any areas with suspected infiltrative margins, even if only partial, were diligently classified as infiltrative margins in the analysis.

### Treatment and outcome measurements

All patients underwent surgical resection and received a pathological diagnosis at our institution. Radiotherapy (RTx) was administered either immediately after surgery or as salvage therapy when recurrence was confirmed. The gross target volume encompassed the residual tumor and the resection cavity, as seen on MRI performed postoperatively or upon recurrence confirmation. Adjuvant postoperative RTx was delivered at a median dose of 54 Gy (range, 30.0–60.0 Gy), with a daily fractionation of 2.0 Gy per fraction. No patients underwent stereotactic radiosurgery as adjuvant postoperative therapy. In case of post-RTx recurrence, surgery was considered a primary option; however, chemotherapy (CTx) was considered when surgery was not feasible. Cyberknife radiosurgery was performed in three cases. CTx predominantly comprised temozolomide or bevacizumab; in one case, a combination of ifosfamide, carboplatin, and etoposide (ICE therapy) was administered as adjuvant CTx following surgery after recurrence.

### End points and statistical analysis

The primary endpoint was progression-free survival (PFS), i.e., the time from diagnosis to local recurrence. The secondary endpoint was the overall survival (OS), i.e., the time from diagnosis to the date of death. The last clinical follow-up was determined as the date at which the most recent follow-up imaging data were obtained. Cox proportional hazard models were used for both univariate and multivariate analyses to assess the prognostic importance of clinicopathological parameters in patients with PXAs. The following variables were assessed: age, sex, tumor location, tumor component, T1 enhancement, tumor margin, peritumoral edema, tumor volume, and the WHO grade. Among these, variables that were significant (*p* < 0.05) in the univariate analysis were incorporated into the multivariate analysis. To ensure inter-rater reliability in the assessment of tumor margins, Fleiss' generalized kappa was utilized. All statistical analyses were performed using SPSS version 26.0 (SPSS Inc., Chicago, Illinois, United States) and R (Version 4.3.1).

### Ethics approval

This study was performed in line with the principles of the Declaration of Helsinki. The study protocol was approved by the Institutional Review Board of the Asan Medical Center on April 3, 2023, with approval number S2023-0703-0001.

### Consent to participate

Informed consent was obtained from all individual participants included in the study.

## Results

### Patient characteristics and radiological features

Table 1 summarizes the demographic data of the patients; the median age was 24 years (range, 7–60 years). The overall median follow-up period was of 63 months (range, 12–259 months); however, for patients with grade 2 and 3 PXAs, the median follow-up durations were of 67 months (range, 12–259 months) and 38 months (range, 15–179 months), respectively. The tumors were located in the superficial cerebral hemisphere, the cerebellum, the brainstem, and a periventricular deep white matter in 40 (81.6%), 2, 1, and 6 patients, respectively. Among the 40 patients with tumors in the cerebral hemisphere, 18 (36.7%) presented with tumors specifically in the temporal lobe. Histopathologically, 40 (81.6%) and 9 (18.4%) patients were diagnosed with grade 2 and 3 PXAs, respectively. The preoperative KPS had a median of 90 (range: 80–100), indicating good performance.

**Table 1:** Demographic and radiological characteristics of the patients.

Characteristics	No. of patients (N = 49)
Sex	
Male, n (%)	21 (57.1)
Female, n (%)	28 (42.9)
Age (years)	
Median (range)	24 (7–60)
Initial symptoms, n (%)	
Seizures	28 (57.1)
Headache	14 (28.6)
Motor weakness	1 (2.0)
Dizziness	3 (6.1)
Diplopia	1 (2.0)
Nausea/vomiting	1 (2.0)
Incidental findings	1 (2.0)
Tumor location, n (%)	
Temporal lobe	18 (36.7)
Non-temporal lobe	31 (63.3)
WHO grade, n (%)	
Grade 2	40 (81.6)
Grade 3	9 (18.4)
Malignant transformation, n (%)	5 (10.2)
Tumor volume (cm^3^)	
Median (range)	19.0 (0.4–164.6)
Tumor margin, n (%)	
Circumscribed	27 (55.1)
Infiltrative	22 (44.9)
Component, n (%)	
Solid	19 (38.8)
Solid + Cystic	23 (46.9)
Cystic	7 (14.3)
T1 enhancement, n (%)	
Strong	30 (61.2)
Weak	19 (38.8)
Peritumoral edema, n (%)	
Minimal	23 (46.9)
Evident	26 (53.1)

WHO, World Health Organization.

The most common initial symptoms at diagnosis were seizures; these occurred in 28 patients (57.1%). Among the patients with an initial symptom of seizures, 16 patients (57.1%) had tumors located in the temporal lobe, while the remaining 12 (42.9%) were in other parts of the cerebral hemisphere. Among patients with preoperative seizures, five individuals (17.8%) continued antiepileptic drug usage after surgery. Of these, one experienced residual tumor progression after STR, another had residual tumor but remained stable after STR, and the remaining three maintained stable after GTR. Out of the total patients, 42 (85.7%) showed either improvement or had symptoms similar to preoperative conditions post-surgery. However, seven patients (14.3%) experienced worsened neurologic deficits after surgery. Among these, five had tumors adjacent to eloquent areas, and despite STR, they experienced persistent neurologic deficits. Of these seven patients, two had Grade 2 tumors, and five had Grade 3 tumors, all of whom experienced tumor recurrence later. Additionally, malignant transformation was observed in five patients among total cohort, with four transitioning from Grade 2 to Grade 3 PXAs and one transforming from Grade 2 PXA to glioblastoma.

We categorized the radiological features of PXA based on previous literature. The varied radiological characteristics of PXAs often make their preoperative differentiation from high-grade gliomas challenging. We noted predominantly cystic lesions with small nodules in seven patients (14.3%), mixed-type lesions in 23 patients (46.9%), and solid-type lesions in 19 patients (38.8%). The tumors were strongly enhanced in a major proportion of the patients (61.2%) and weakly enhanced in a smaller proportion of the patients (38.8%). Furthermore, peritumoral edema was minimal in 23 patients (46.9%) and evident in 26 patients (53.1%). Tumor margins were circumscribed in 27 patients (55.1%) and infiltrative in 22 patients (44.9%). The average tumor volume was 19.0 cm^3^ (range, 0.4–164.6 cm^3^).

### Overall outcomes and prognostic factors

Overall, 18 patients experienced a recurrence; the recurrence rates in patients with grade 2 PXAs and in those with grade 3 PXAs were 27.5% (11/40) and 77.8% (7/9), respectively. During the follow-up period, death events occurred in 20% (8/40) in patients with grade 2 PXAs and 55.6% (5/9) of those with grade 3 PXAs, respectively.

Kaplan–Meier analysis revealed a markedly poorer OS in patients with grade 3 PXAs than in those with grade 2 PXAs (median: 64 months vs. not reached, *p* = 0.014); the PFS also differed significantly between the two patient groups (median: 48 months vs. not reached, *p* = 0.005). The 5-year PFS rates in patients with grade 2 PXAs and in those with grade 3 PXAs were 75.8% and 37.0%, respectively; the corresponding 5-year OS rates were 91.4% and 64.8%, respectively.

Univariate analysis revealed that strong T1 enhancement (*p* = 0.036), infiltrative tumor margins (*p* < 0.001), and peritumoral edema (*p* = 0.003) were associated with a significantly poor PFS. The PFS was prolonged in patients with grade 2 PXAs as compared to in those with grade 3 PXAs (*p* = 0.005). Furthermore, the PFS was notably prolonged in patients who underwent GTR as compared to those who underwent STR (*p* = 0.005). Conversely, sex, age, temporal location, presence of cystic components, and tumor volume were not significantly associated with the PFS. Subsequent multivariate analysis revealed the following significant prognostic indicators of a shorter PFS: infiltrative tumor margins (hazards ratio [HR]: 5.306; 95% Confidence Interval [CI]: 1.536–16.515; *p* = 0.008) and a high WHO grade (HR: 4.097; 95% CI: 1.407–11.928; *p* = 0.010).

Univariate analysis revealed that female sex (*p* = 0.028), older age (*p* = 0.033), strong T1 enhancement (*p* = 0.004), infiltrative tumor margins (*p* = 0.001), evident peritumoral edema (*p* = 0.001), WHO grade 3 (*p* = 0.014), and STR versus GTR (*p* = 0.004) were significantly associated with worse OS outcomes. Subsequent multivariate analysis revealed the following significant prognostic indicators of a poor OS (Tables 2 and 3): infiltrative tumor margins (HR: 6.444; 95% CI: 1.232–33.705; *p* = 0.027) and a high WHO grade (HR: 5.291; 95% CI: 1.207–23.187; *p* = 0.027).

**Table 2:** Univariate and multivariate analyses of the PFS.

	No. of recurrence	Univariate		Multivariate		
	/No. of patients (%)	at 5 years	Log-rank	HR	95% CI	*p* value
Overall	18/49 (36.7)	69.2 ± 6.9	–			
Sex						
Male	5/21 (23.8)	80.4 ± 8.8	0.111			
Female	13/28 (46.4)	60.3 ± 9.9				
Age (years)						
≥ 30	10/20 (50.0)	62.4 ± 11.5	0.151			
< 30	8/29 (27.6)	74.1 ± 8.5				
Location						
Temporal	4/18 (22.2)	75.2 ± 10.9	0.270			
Non-temporal	14/31 (45.2)	66.3 ± 8.8				
Cystic component						
Solid	9/19 (47.4)	61.2 ± 11.6	0.264			
Solid + Cystic	8/23 (34.8)	65.0 ± 10.9				
Cystic	1/7 (14.3)	100.0				
Tumor volume (cm^3^)						
≥ 50	5/10 (50.0)	43.8 ± 17.5	0.543			
< 50	13/39 (33.3)	59.3 ± 10.2				
T1 enhancement						
Strong	14/30 (46.6)	53.4 ± 9.7	0.036*	1.167	0.279–4.877	0.832
Weak	4/19 (21.1)	93.8 ± 6.1				
Tumor margin						
Infiltrative	13/22 (59.1)	47.5 ± 11.0	< 0.001*	5.036	1.536–16.515	0.008*
Circumscribed	5/27 (18.5)	86.1 ± 7.5				
Peritumoral edema						
Minimal	4/23 (17.4)	90.4 ± 6.5	0.003*	1.984	0.446–8.825	0.368
Evident	14/26 (53.8)	50.2 ± 10.5				
WHO grade						
2	11/40 (27.5)	75.8 ± 7.1	0.005*	4.097	1.407–11.928	0.010*
3	7/9 (77.8)	37.0 ± 18.7				
EOR						
GTR	9/35 (25.7)	77.3 ± 7.6	0.005*	2.288	0.778–6.725	0.132
STR	9/14 (64.3)	49.0 ± 13.6				

CI, confidence interval; EOR, extent of resection; GTR, gross total resection; HR, hazards ratio; PFS, progression-free survival; SE, standard error; STR, subtotal resection; WHO, World Health Organization.

*p < 0.05.

**Table 3:** Univariate and multivariate analyses of the OS.

	No. of deaths	Univariate		Multivariate		
	/No. of patients (%)	at 5 years ([%] ± SE)	Log-rank	HR	95% CI	*p* value
Overall	13/49 (36.5)	83.7 ± 5.7	–			
Sex						
Male	2/21 (9.5)	90.2 ± 6.6	0.028*	1.998	0.278–14.367	0.492
Female	11/28 (39.3)	77.9 ± 9.0				
Age (years)						
≥ 30	9/20 (45.0)	84.2 ± 7.3	0.033*	2.093	0.569–7.699	0.266
< 30	4/29 (13.8)	82.5 ± 9.5				
Location						
Temporal	2/18 (11.1)	93.8 ± 6.1	0.129			
Non-temporal	11/31 (35.5)	78.0 ± 8.1				
Cystic component						
Solid	8/19 (42.1)	77.4 ± 10.0	0.141			
Solid + Cystic	5/23 (21.7)	89.7 ± 7.0				
Cystic	0/7 (0.0)	100.0				
Tumor volume (cm^3^)						
≥ 50	3/10 (30.0)	62.2 ± 17.8	0.387			
< 50	10/39 (33.3)	61.5 ± 10.2				
T1 enhancement						
Strong	12/30 (40.0)	77.9 ± 8.1	0.004*	3.889	0.363–41.703	0.262
Weak	1/19 (5.3)	100.0				
Tumor margin						
Infiltrative	11/22 (50.0)	76.2 ± 9.3	0.001*	6.444	1.232–33.705	0.027*
Circumscribed	2/27 (7.4)	94.7 ± 5.1				
Peritumoral edema						
Minimal	1/23 (4.3)	95.5 ± 4.4	0.001*	3.422	0.240–48.794	0.364
Evident	12/26 (46.2)	72.8 ± 9.8				
WHO grade						
2	18/40 (20.0)	91.4 ± 4.8	0.014*	5.291	1.207–23.187	0.027*
3	5/9 (55.6)	64.8 ± 16.5				
EOR						
GTR	5/35 (14.3)	93.0 ± 4.8	0.004*	3.405	0.898–12.913	0.072
STR	8/14 (57.1)	62.5 ± 13.5				

CI, confidence interval; EOR, extent of resection; GTR, gross total resection; HR, hazards ratio; OS, overall survival; SE, standard error; STR, subtotal resection; WHO, World Health Organization.

*p < 0.05.

In our study, a subgroup analysis was conducted focusing on 40 Grade 2 patients. In the univariate analysis, factors such as infiltrative margin (*p* = 0.007), evident peritumoral edema (*p* = 0.003), and STR (*p* = 0.021) emerged as significant contributors to poorer PFS. However, in the multivariate analysis, no factors reached statistical significance. Notably, although infiltrative tumor margin did not achieve statistical significance (*p* = 0.088), it exhibited a noteworthy tendency, prompting the need for future large-scale studies. As for factors associated with poorer OS, the univariate analysis highlighted female gender (*p* = 0.023), age over 30 at diagnosis (*p* = 0.007), strong enhancement in T1 (*p* = 0.021), infiltrative tumor margin (*p* = 0.003), evident peritumoral edema (*p* = 0.001), and STR (*p* = 0.026). However, none of these factors demonstrated statistical significance in the multivariate analysis. In the univariate analysis involving 9 Grade 3 patients, male gender (*p* = 0.005) and infiltrative tumor margin (*p* = 0.050) were associated with poorer PFS, while no factors showed statistical significance in relation to poorer OS. GTR demonstrated marginal significance in both PFS (*p* = 0.061) and OS (*p* = 0.066). The results of the subgroup analysis related to PFS and OS for Grade 2 are presented in supplementary tables S1 and S2, respectively, and the results for Grade 3 are described in supplementary table S3.

### GTR versus STR: the role of adjuvant RTx

Among the 40 patients with grade 2 PXAs, 30 and 10 patients underwent GTR and STR, respectively. Patients who underwent GTR did not receive adjuvant RTx; among these, five patients (16.7%) experienced a local recurrence at the tumor surgical site. Six of the 10 patients who underwent STR received adjuvant RTx; two of these patients as well as the remaining four patients who did not receive adjuvant RTx experienced a local recurrence. Thus, the recurrence rate was 60%. Among the nine patients with grade 3 PXAs, six and three patients underwent GTR and STR, respectively. Five patients who underwent GTR and all patients who underwent STR received adjuvant RTx; the remaining one patient who underwent GTR did not receive adjuvant RTx due to dermatological concerns.

### Treatment after recurrence

Treatment courses undertaken in recurrent cases are detailed in Table 4. Reoperation was prioritized whenever feasible, regardless of the recurrences. In cases of recurrent grade 2 PXAs, GTR was performed; adjuvant RTx was administered in some cases, whereas the patients were observed without RTx administration in other cases. In cases with a history of RTx, adjuvant CTx was considered a salvage treatment following surgery. Particularly in grade 3 PXA cases (n = 4), adjuvant RTx was administered irrespective of the EOR after primary surgery; thus, adjuvant CTx was administered postoperatively after the initial recurrence: among these patients, three received temozolomide and one received the ICE therapy.

**Table 4:** Salvage treatment strategies and prognosis of patients with a recurrence.

#	Sex/Age (years)	At diagnosis						At the first recurrence			At the second recurrence			OS (mo)	Outcome
		Location	Tumor size (cm)	WHO grade	Tumor margin	EOR (first op)	Adjuvant Tx	WHO grade	First PFS (mo)	First salvage Tx	WHO grade	Second PFS (mo)	Second salvage Tx		
1	M/7	Rt. O	6.0	2	Circum	GTR	No	2	51	Treatment refusal	2	Stable	Stable	135	Alive
2	F/32	Lt. O	7.0	2	Circum	GTR	No	2	28	RTx	2	19	CKRS	58	Deceased
3	F/11	Lt. T	3.2	2	Infiltrative	GTR	No	2	37	OP	2	18	OP	259	Alive
4	F/40	Rt. F	2.6	2	Infiltrative	GTR	No	3	21	OP	3	21	OP + CTx (bevacizumab)	63	Deceased
5	F/35	Rt. Cbll	3.5	2	Infiltrative	GTR	No	2	26	OP + adjuvant RTx	2	48	CTx (TMZ)	96	Deceased
6	F/39	Rt. F	1.8	2	Infiltrative	STR	No	2	71	OP + adjuvant RTx	3	13	OP + CTx (TMZ)	96	Deceased
7	F/23	Lt. Cbll	3.3	2	Infiltrative	STR	No	3	10	OP + adjuvant RTx	3	24	CKRS + CTx (bevacizumab)	60	Deceased
8	M/7	Rt. T	5.0	2	Infiltrative	STR	RTx	2	5	OP	2	25	OP	49	Alive
9	M/30	Lt. T	8.6	2	Infiltrative	STR	No	2	3	OP	2	8	OP + CKRS	20	Deceased
10	F/17	Midbrain	3.5	2	Infiltrative	STR	RTx	2	4	OP	2	6	Treatment refusal	19	Deceased
11	M/10	Rt. P	4.5	2	Circum	STR	No	2	104	SRS	2	Stable	Stable	104	Alive
12	F/60	Lt. F	4.8	3	Infiltrative	GTR	RTx	3	8	Treatment refusal	3	Deceased	Deceased	16	Deceased
13	M/22	Lt. T	4.7	3	Infiltrative	STR	RTx	3	14	Treatment refusal	3	Deceased	Deceased	17	Deceased
14	F/37	Rt. P	5.5	3	Circum	GTR	RTx	3	178	Treatment refusal	3	Stable	Stable	179	Alive
15	F/38	Lt. F	4.7	3	Infiltrative	GTR	RTx	3	15	OP + CTx (TMZ)	3	25	OP + CTx (metronomic TMZ)	74	Deceased
16	F/38	Lt. F	2.9	3	Infiltrative	GTR	RTx	3	91	OP + CTx (TMZ)	3	Stable	Stable	114	Alive
17	F/20	Lt. T	4.9	3	Infiltrative	STR	RTx	3	12	OP + CTx (TMZ)	3	3	CTx (bevacizumab)	21	Deceased
18	F/57	Rt. T	3.6	3	Circum	STR	RTx	3	48	OP + CTx (ICE)	3	Deceased	Deceased	64	Deceased

Cbll, cerebellum; Circum, circumscribed; CKRS, Cyberknife radiosurgery; CTx, chemotherapy; EOR, extent of resection; F, frontal lobe; GTR, gross total resection; ICE, ifosfamide, carboplatin, etoposide; Lt., left; mo, months; O, occipital lobe; OP, operation; OS, overall survival; P, parietal lobe; PFS, progression-free survival; Rt., right; RTx, radiotherapy; STR, subtotal resection; T, temporal lobe; TMZ, temozolomide.

## Discussion

In this study, we analyzed the correlations between various radiological profiles (including the degree of contrast enhancement, peritumoral edema, and cystic components) and prognosis. Multivariate analysis did not identify these factors as significant prognostic indicators of the PFS and OS. Limited literature exists on the associations between radiological features and PXA prognosis. A significant finding of our study was the impact of tumor margins on the prognosis of PXAs. While PXAs are classified as circumscribed gliomas, our findings revealed that an ill-defined, infiltrative margin significantly impacted both the PFS and OS in affected patients. PXAs typically present with a well-circumscribed margin and are accompanied by mural nodules with a cystic main portion^2^. However, tumors mainly presenting as solid masses may exhibit extensive peritumoral edema and vibrant enhancement with a poorly demarcated margin. These tumors are challenging to distinguish from high-grade gliomas radiologically^3,4^. In our study, 22 patients (44.9%) presented with infiltrative margins: 17 and 3 of these had grade 2 and 3 PXAs, respectively. Additionally, we found that the tumor margin and WHO grade were not significantly correlated (*p* = 0.487), confirming their independent roles as prognostic factors. These findings underscore the importance of tumor margins in PXAs and suggest that the tumor’s dissemination pattern and biological characteristics play a pivotal role in prognosis beyond pathological features. Studies that have pathologically elucidated the tumor margins of PXAs are lacking; however, instances of pathologically confirmed margin infiltration have been documented in other tumors classified as circumscribed gliomas (such as pilocytic astrocytomas)^7^. Given the significant peritumoral edema and pleomorphic features of PXAs, reconsidering the potential for margin infiltration is important. Our findings emphasize the need to consider more proactive treatment approaches in PXA cases with infiltrative tumor margins.

Some studies have indicated that evident peritumoral edema is associated with poor PFS^8,9^. Byun et al. examined the relationship between the PFS and cortical location, calcification, peritumoral edema, diffusion restriction, hyperperfusion, and hypermetabolism on nuclear images in 25 patients^8^. Their univariate analysis revealed that evident peritumoral edema was associated with a poor PFS (*p* = 0.01); however, they did not perform a multivariate analysis. In our study, univariate analysis revealed significant associations between peritumoral edema and both the PFS and OS (*p* = 0.0006 and 0.010, respectively); however, multivariate analysis did not find these associations significant (*p* = 0.256 and *p* = 0.274, respectively).

The mechanism underlying peritumoral edema in PXAs remains largely unexplored, with most studies focusing on diffuse glial tumors^10–12^. In such cases, the occurrence of peritumoral edema in these tumors is explained by potential tumor cell infiltration and the establishment of a tumor-supportive microenvironment involving immunosuppressive effects^11^. Our study found no significant correlations between peritumoral edema and infiltrative tumor margins (*p* = 0.103). Only the tumor margin was a significant prognostic predictor, and edema did not seem to influence the prognosis. Tumor invasiveness itself may not be directly proportional to the surrounding tumor environment. Further pathological investigations in larger patient cohorts, particularly focusing on the roles of peritumoral edema in both circumscribed and diffuse gliomas, are warranted.

In previous studies, GTR has been regarded as the most effective prognostic factor for survival^13–15^. However, even these findings have not always shown concordance; some studies have indicated that while GTR improves the PFS, it does not impact the OS^16^. Moreover, reports have suggested that GTR does not improve either the PFS or the OS^8,9,17^. Our study also found no significant relationship between GTR and the PFS and OS. This observation held consistent for analyses considering the entire study cohort and when performing subgroup analyses specifically targeting grade 2 and 3 patients. The diverse outcomes observed in various studies might be attributed to the low incidence rate of PXAs, resulting in predominantly smaller study populations; this underscores the need for caution while interpreting the findings. In our study, none of the patients with grade 2 PXAs who underwent GTR received adjuvant therapy. Among the 10 patients who underwent STR, six received adjuvant RTx, and four were placed under observation. Interestingly, six of these patients experienced a recurrence. Notably, adjuvant RTx administration after STR was not associated with recurrence or the OS (*p* = 0.715). The correlation between adjuvant RTx and prognosis has shown limited relevance in previous research; most studies have indicated no beneficial impacts of RTx^18–22^. A recent systematic review and meta-analysis of 167 patients with grade 2 PXAs revealed that adjuvant therapy did not significantly influence the PFS or OS; this suggested the limited impact of routine adjuvant RTx in this context^18^. Khalafallaha et al. have even reported higher mortality rates in 546 adult patients with PXAs who received adjuvant RTx^20^. The association between adjuvant RTx and poor prognosis may be attributed to factors such as the EOR or a higher Ki-67 index. Assessing the effects of RTx on prognosis is complicated by multiple confounding factors in clinical decisions and is exacerbated by the diverse outcomes in small-scale studies. In our study, for grade 2 PXAs, observation alone following GTR was considered effective for tumor control; this is consistent with the latest European Association of Neuro-Oncology/Society for Neuro-Oncology guidelines^23^. For grade 3 PXAs, adjuvant RTx was administered regardless of the EOR. However, four experienced a recurrence among the five patients who underwent GTR, and all patients who underwent STR experienced a recurrence. This highlights the aggressiveness of grade 3 PXAs and the need for aggressive management.

In the context of PXAs, the role of adjuvant CTx as a systemic therapy is not prominent; furthermore, studies on its therapeutic effects are scarce. Conventional chemotherapeutic agents, such as temozolomide or bevacizumab, are typically chosen when surgical intervention is not feasible or radiotherapy is not viable. However, the efficacy of these agents appears to be limited^18,19^. Recent molecular studies have extensively investigated PXAs. Notably, approximately 70% of patients with PXA exhibit the *BRAF V600E* mutation^24,25^. Moreover, reports of radiological and clinical responses when *BRAF* inhibitors, such as vemurafenib and dabrafenib, are used as monotherapy or in combination with mitogen-activated protein kinase inhibitors (such as trametinib) have been documented^26–29^. Our study included 9 cases subjected to genetic analysis, with 7 individuals (77.8%) exhibiting the *BRAF V600E* mutation, and 4 (44.4%) presenting with *CDKN2A/B* homozygous deletion. Regrettably, we could not include cases in the analysis due to the absence of instances involving the use of BRAF inhibitors for disease treatment. However, recent research findings on the role of BRAF inhibitors underscore the potential of alternative therapeutic modalities, prompting anticipation for large-scale future investigations to unveil their efficacies.

## Limitations

This study had some limitations. First, the study design was retrospective, and pathological examinations confirming margin infiltration were not performed. The radiologic evaluation of tumor margins involved a comprehensive evaluation of diverse MR sequences, acknowledging the inherent subjectivity entailed in this evaluation. Second, molecular profiling of tumors has only recently become widespread; thus, genetic profiling data were available for only nine patients from the entire cohort. Therefore, we could not analyze molecular factors such as the *BRAF V600E* mutation, which are reported to be potentially relevant to PXA prognosis^13,16,30,31^.

## Conclusions

Our findings emphasize the significant prognostic influence of infiltrative tumor margins in PXA cases. Infiltrative tumor margins are correlated with an unfavorable PFS and OS; thus, more aggressive therapeutic strategies are required. Our study further underscores the requirement for molecular investigations and broader multicenter prospective studies to contribute to the evolving PXA management guidelines.

### Supplementary Information

Supplementary Figure 1.

Supplementary Table S1.

Supplementary Table S2.

Supplementary Table S3.

## Data Availability

The datasets generated during the current study are available from the corresponding author on reasonable request.
