# Supplementary material for: Exploring prognostic factors and treatment strategies for long-term survival in pleomorphic xanthoastrocytoma patients
Source: Sci Rep. 2024 Feb 26;14:4615. doi: 10.1038/s41598-024-55202-6 (PMC10897451; doi:10.1038/s41598-024-55202-6)
Supplement: Supplementary file 1 — Supplementary Figure 1. [file 41598_2024_55202_MOESM1_ESM.pptx]

## Slide 1
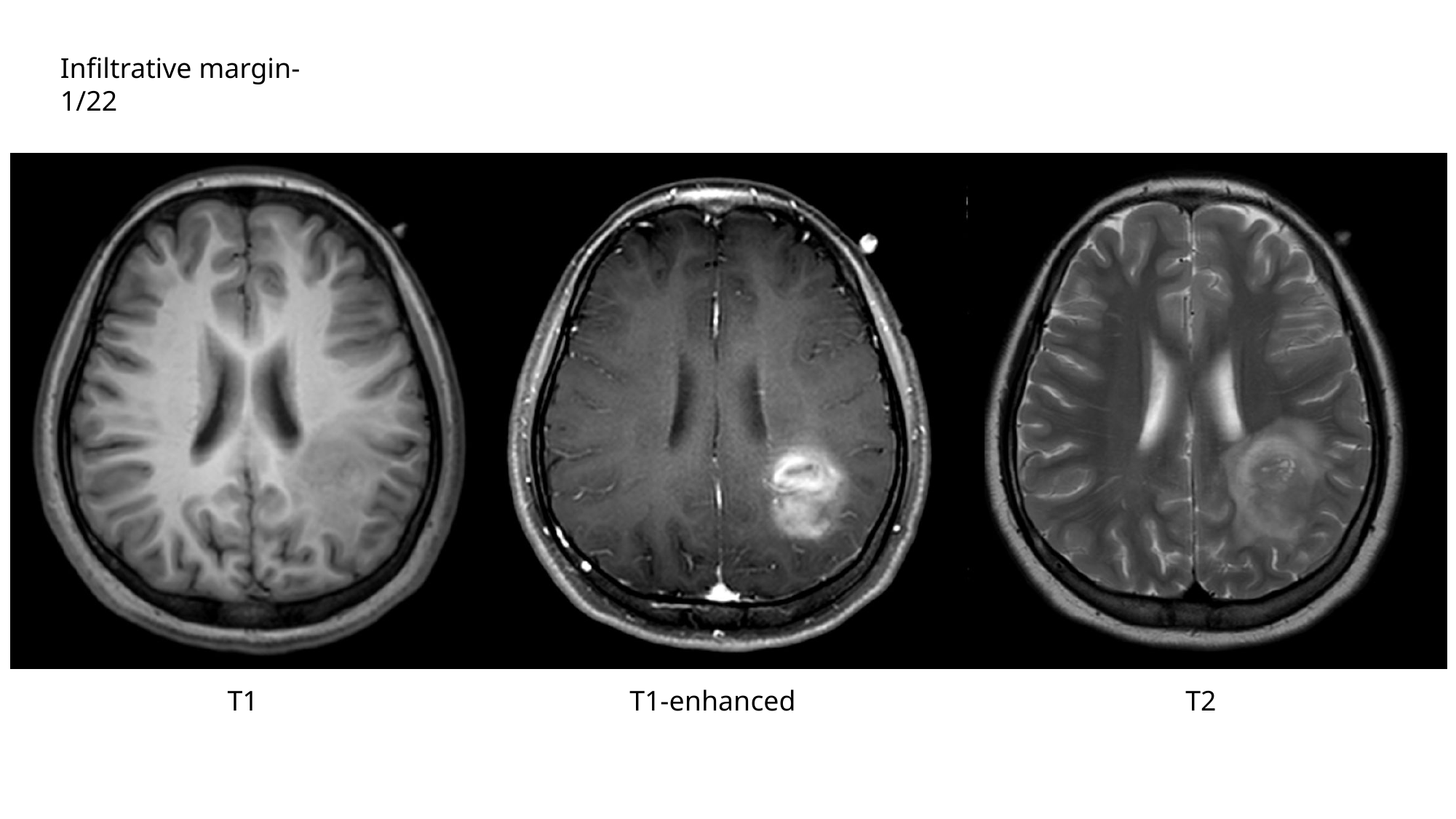

Infiltrative margin-1/22
T1
T1-enhanced
T2

## Slide 2
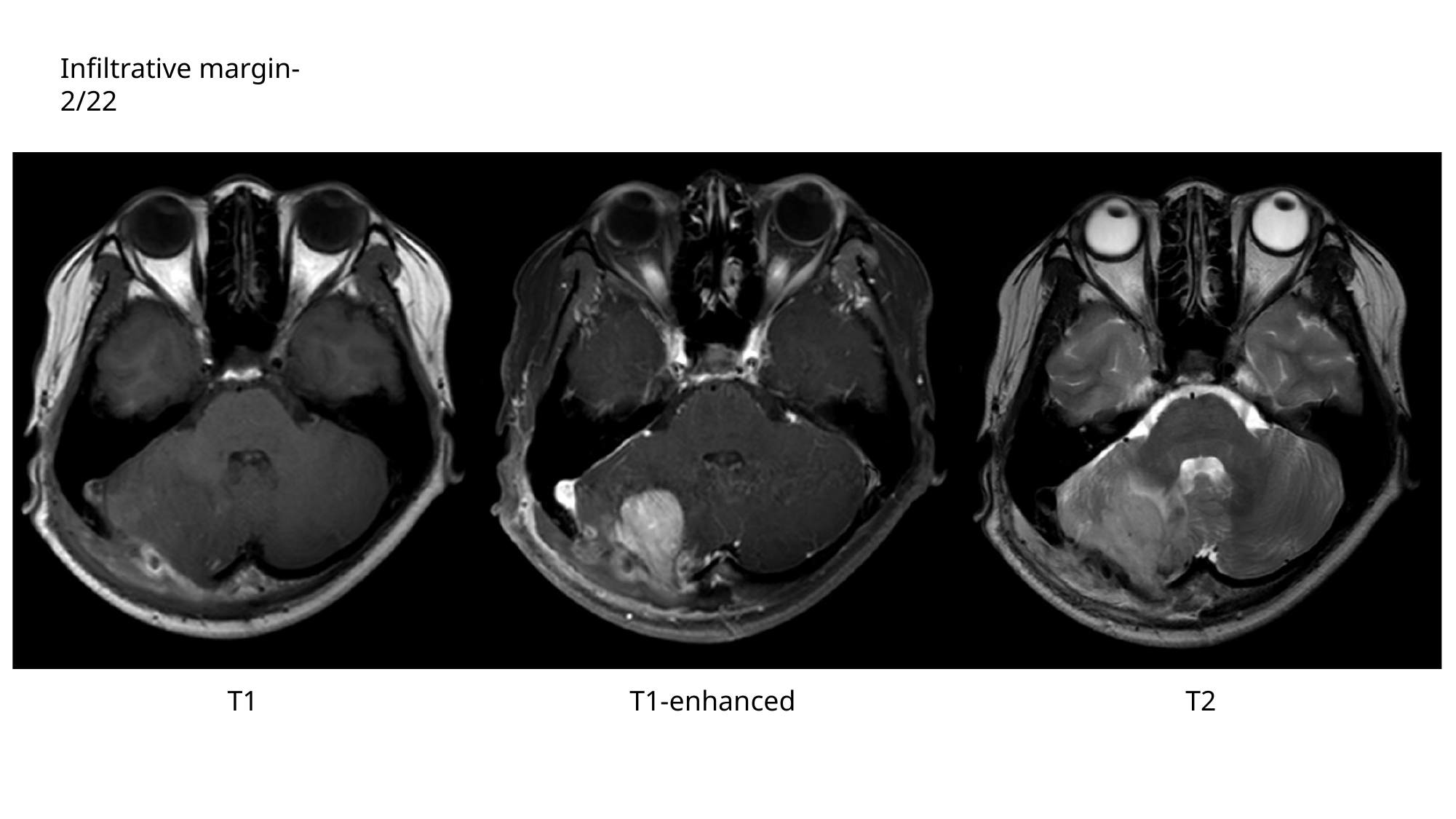

Infiltrative margin-2/22
T1
T1-enhanced
T2

## Slide 3
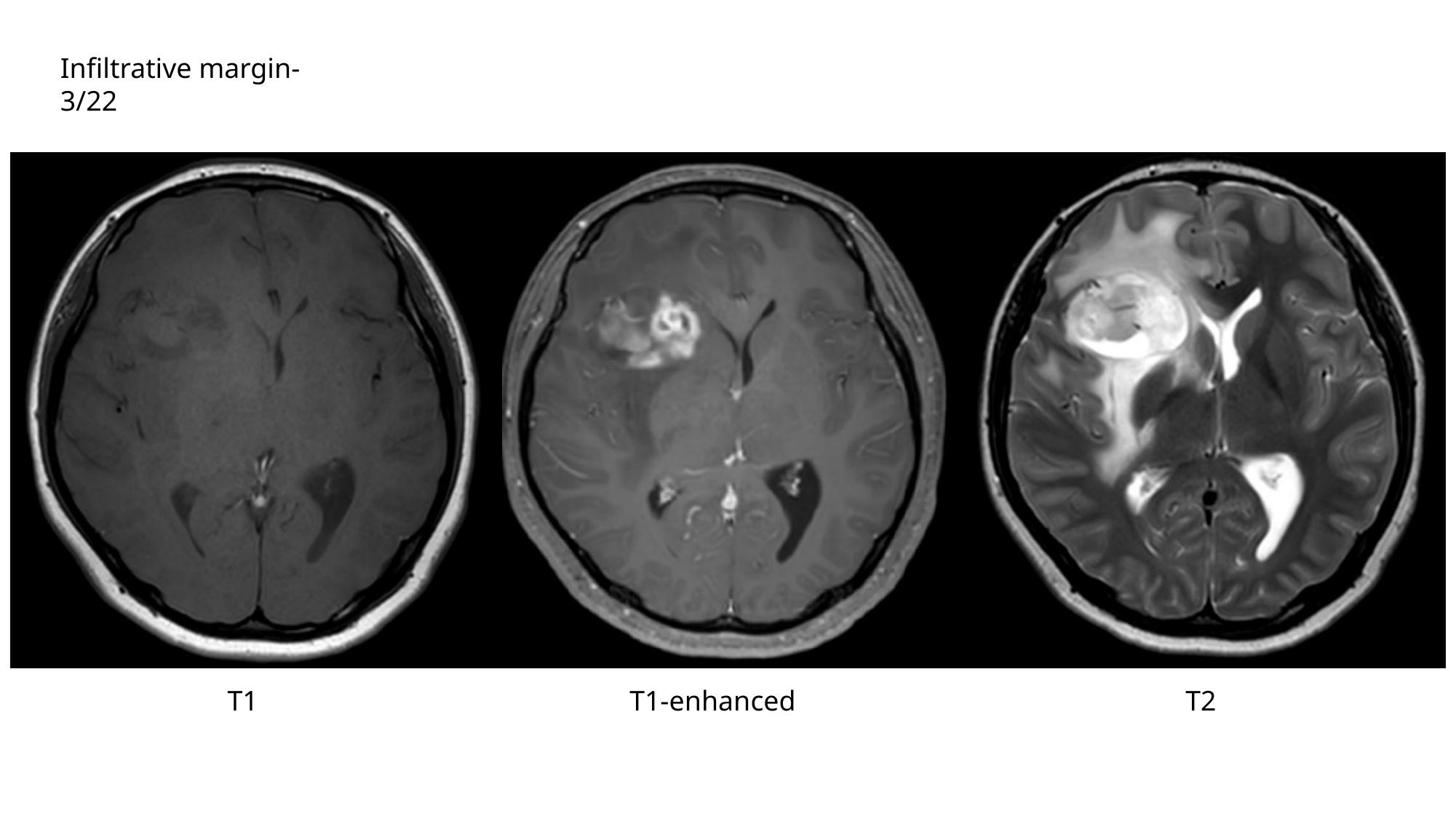

Infiltrative margin-3/22
T1
T1-enhanced
T2

## Slide 4
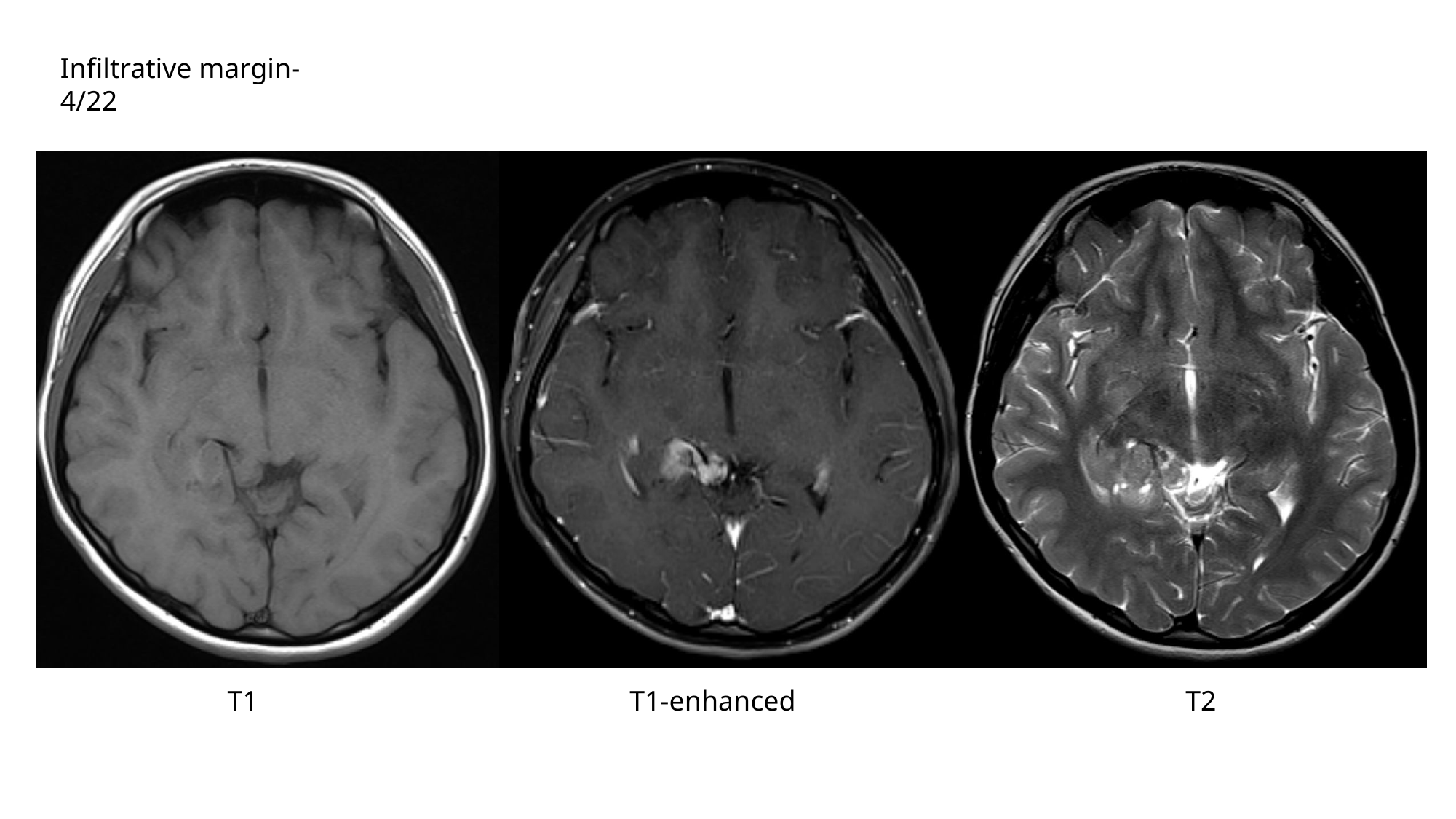

Infiltrative margin-4/22
T1
T1-enhanced
T2

## Slide 5
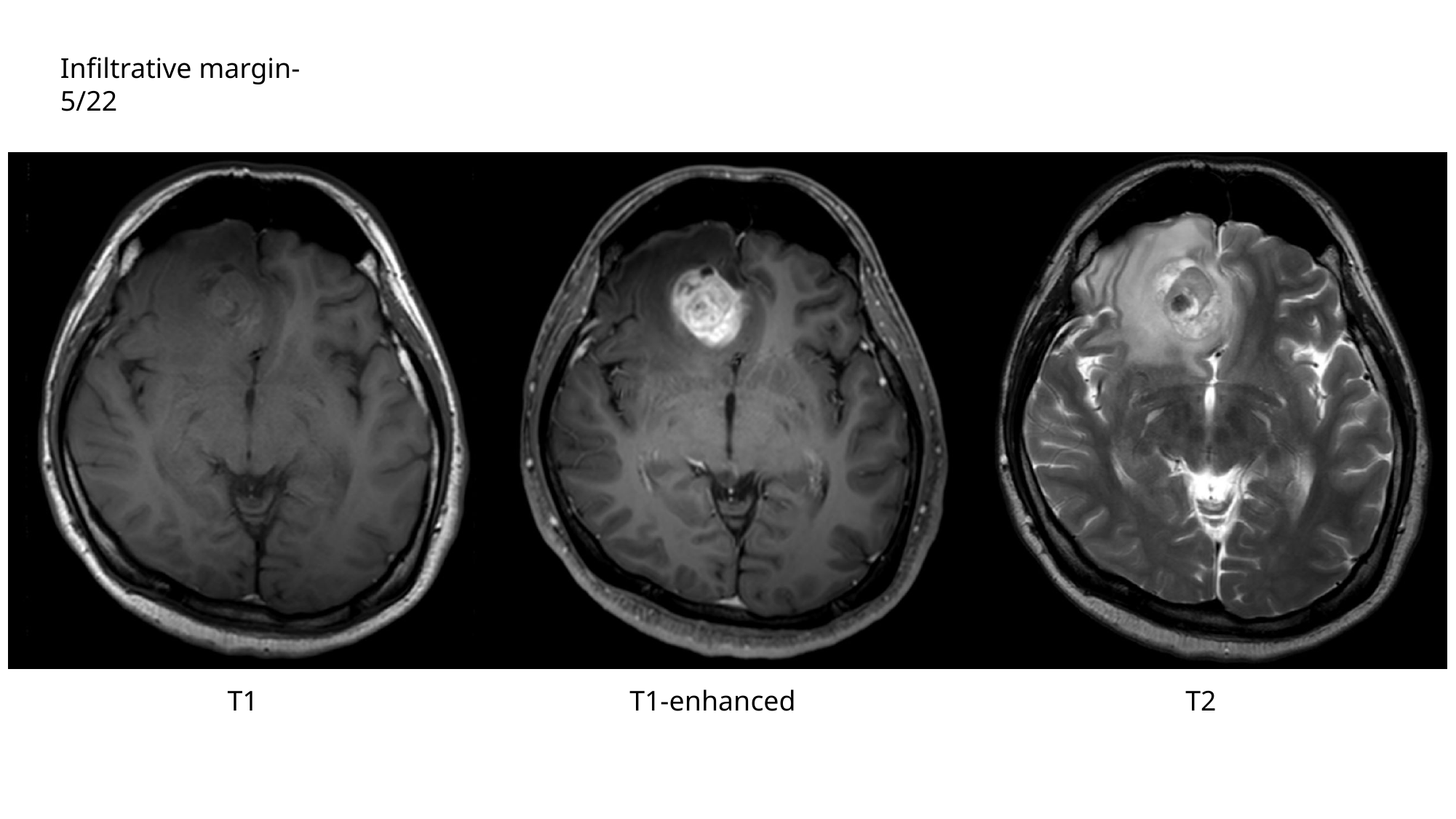

Infiltrative margin-5/22
T1
T1-enhanced
T2

## Slide 6
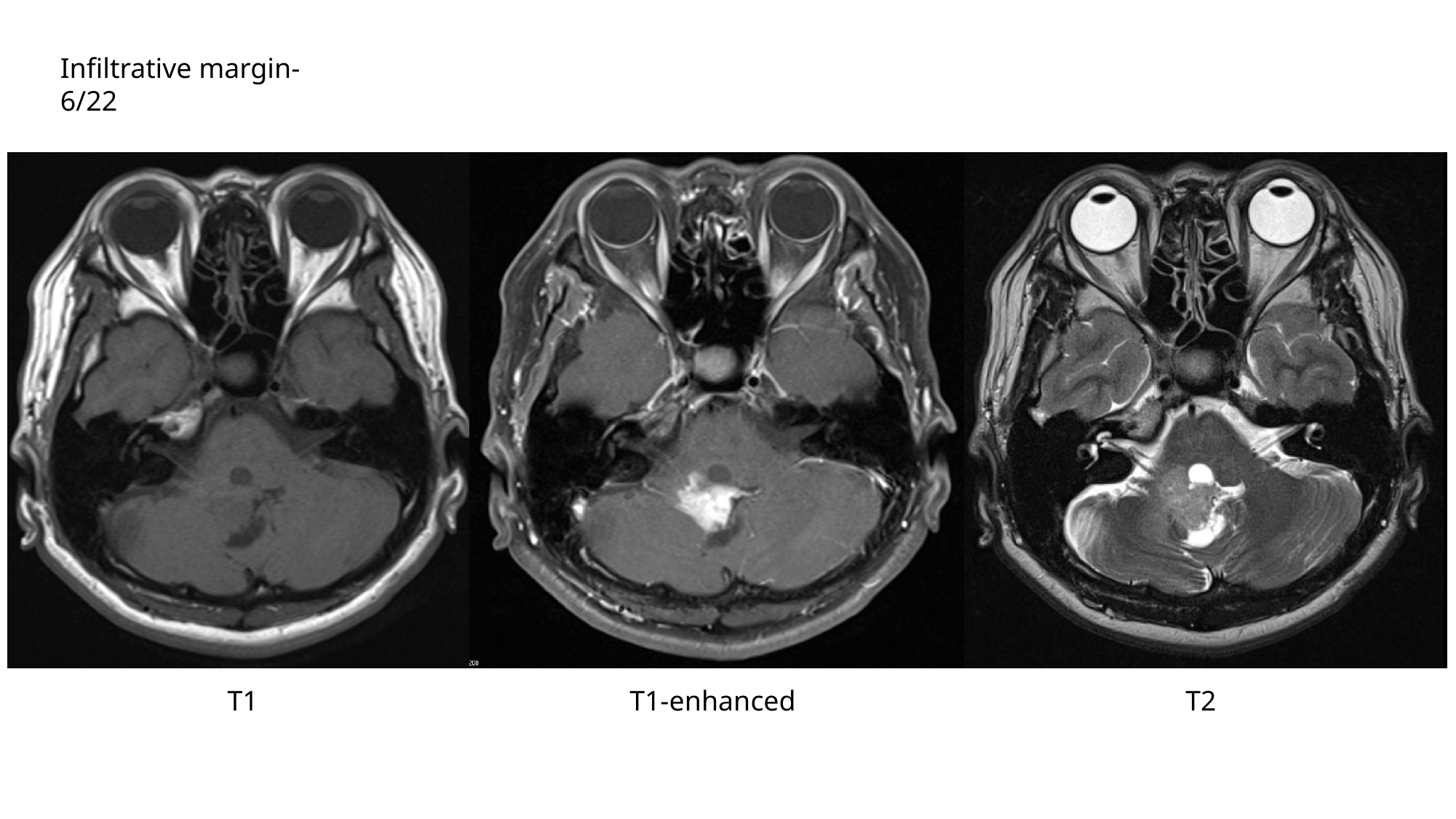

Infiltrative margin-6/22
T1
T1-enhanced
T2

## Slide 7
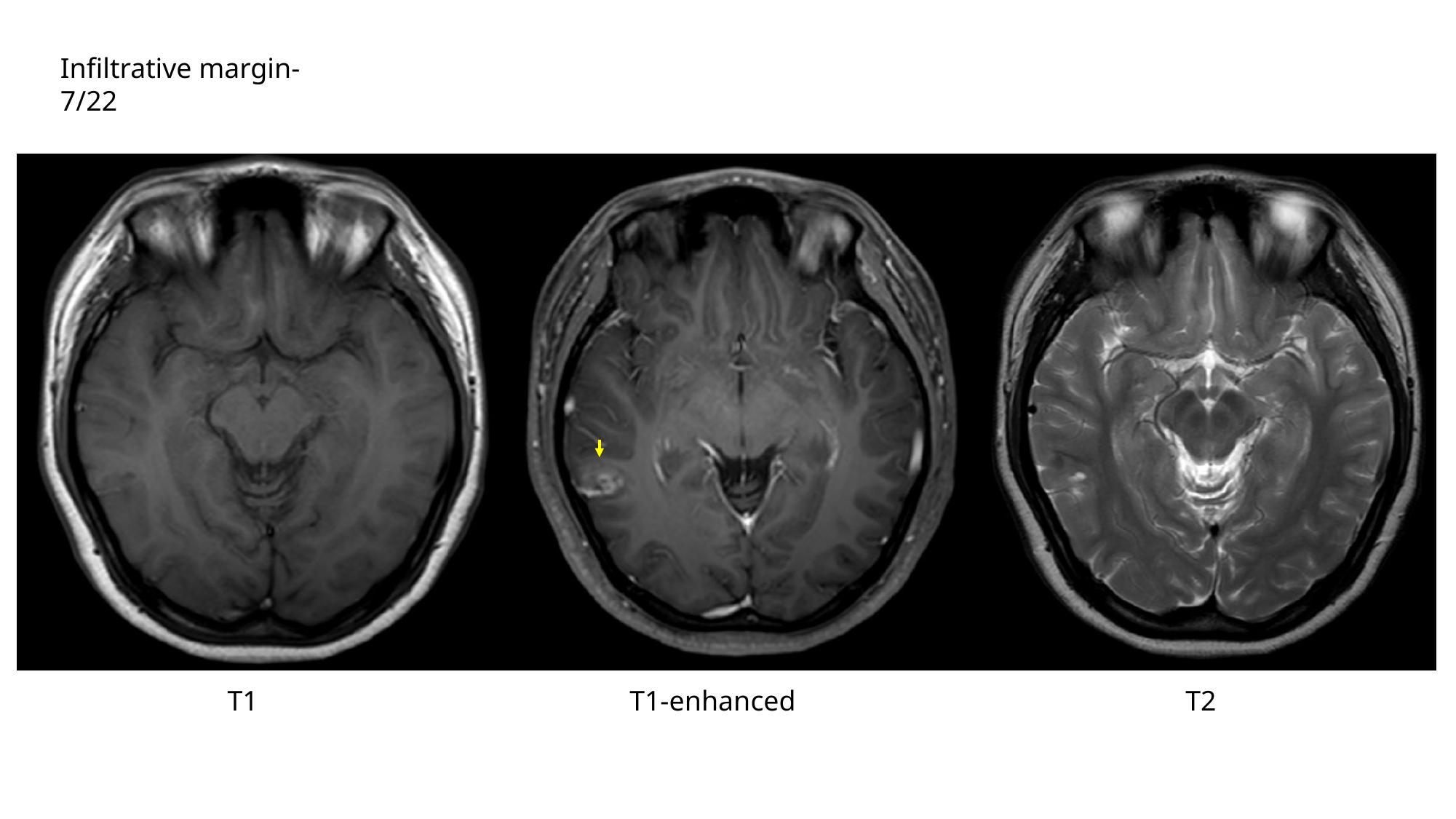

Infiltrative margin-7/22
T1
T1-enhanced
T2

## Slide 8
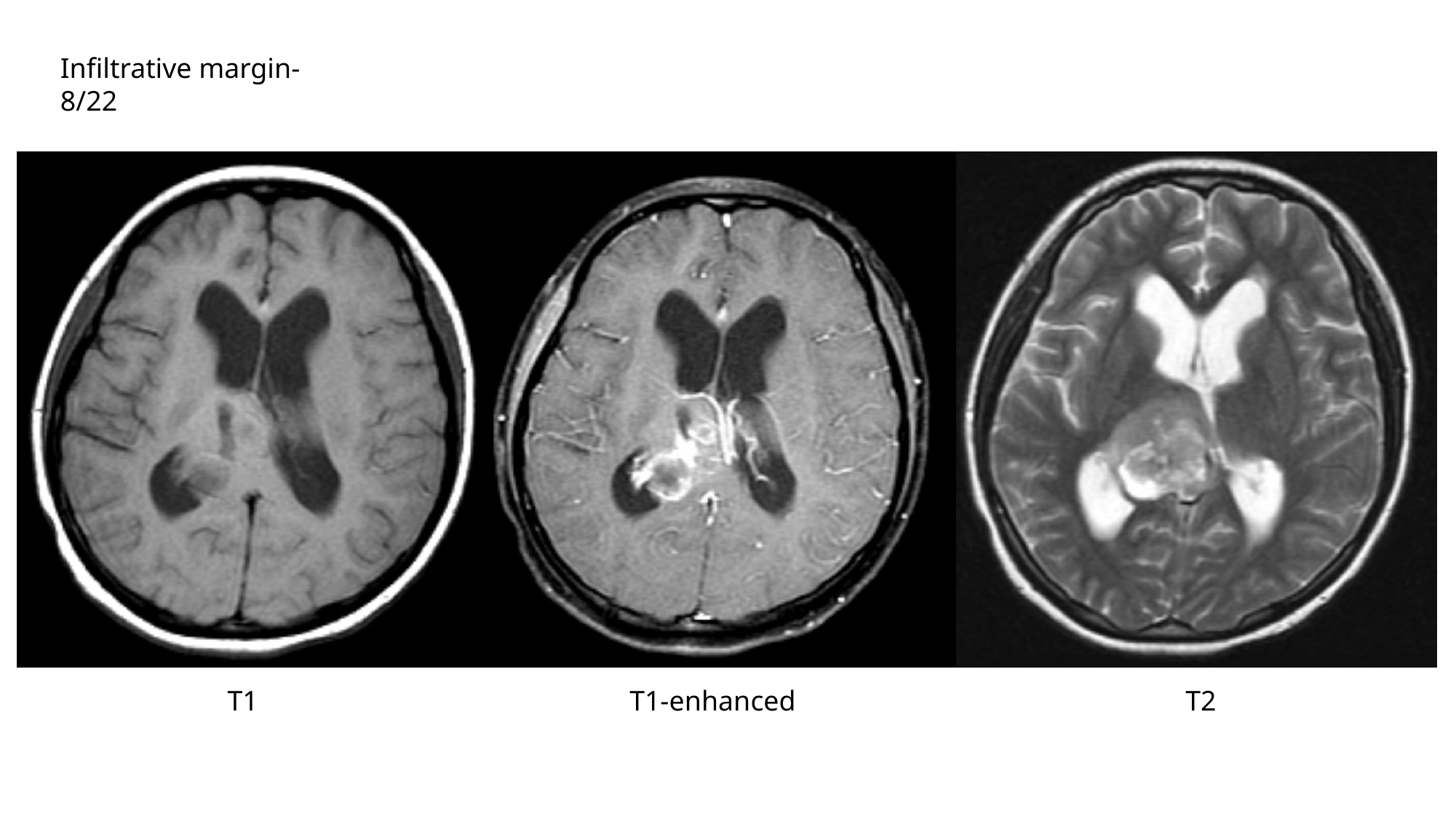

Infiltrative margin-8/22
T1
T1-enhanced
T2

## Slide 9
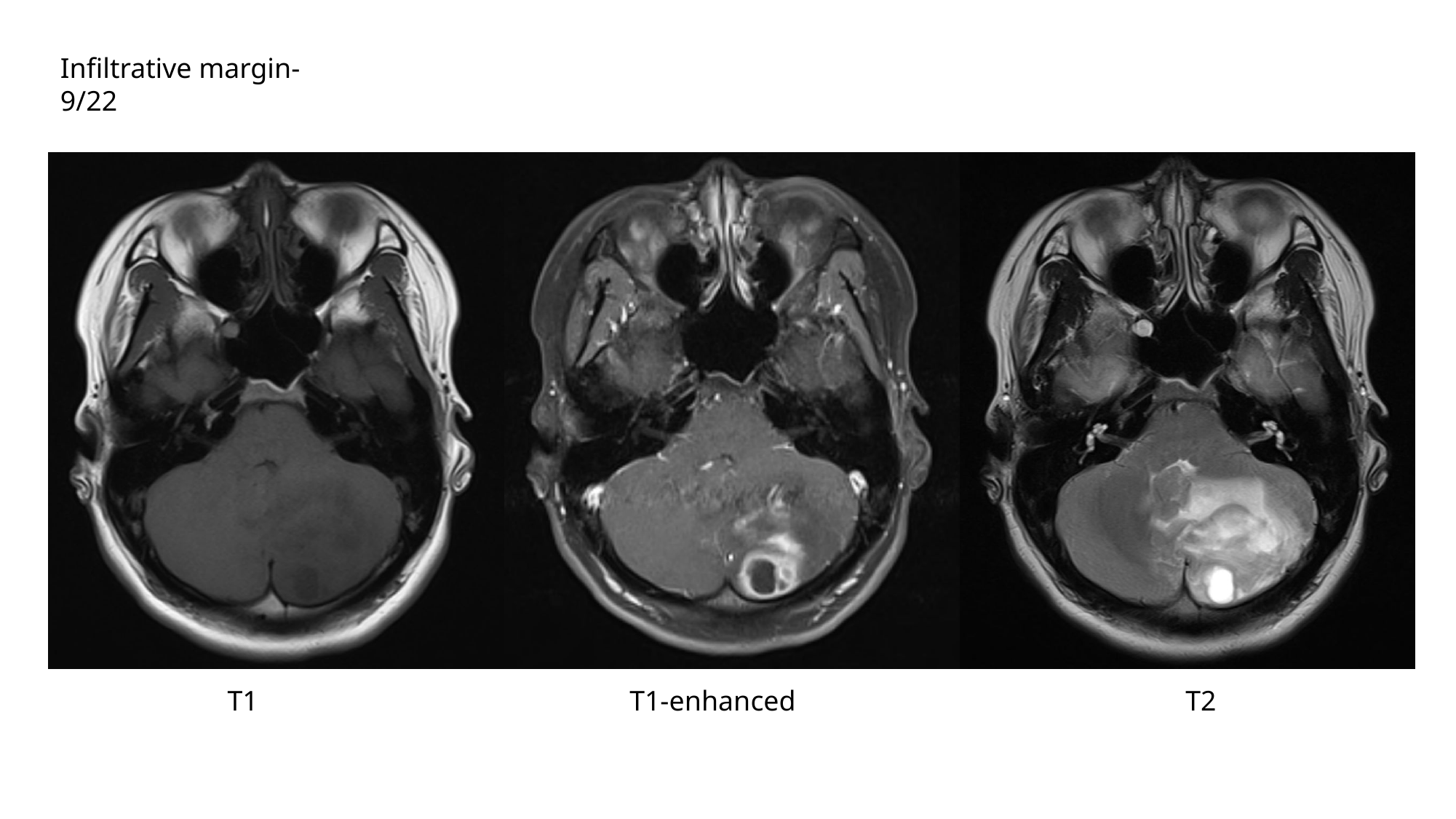

Infiltrative margin-9/22
T1
T1-enhanced
T2

## Slide 10
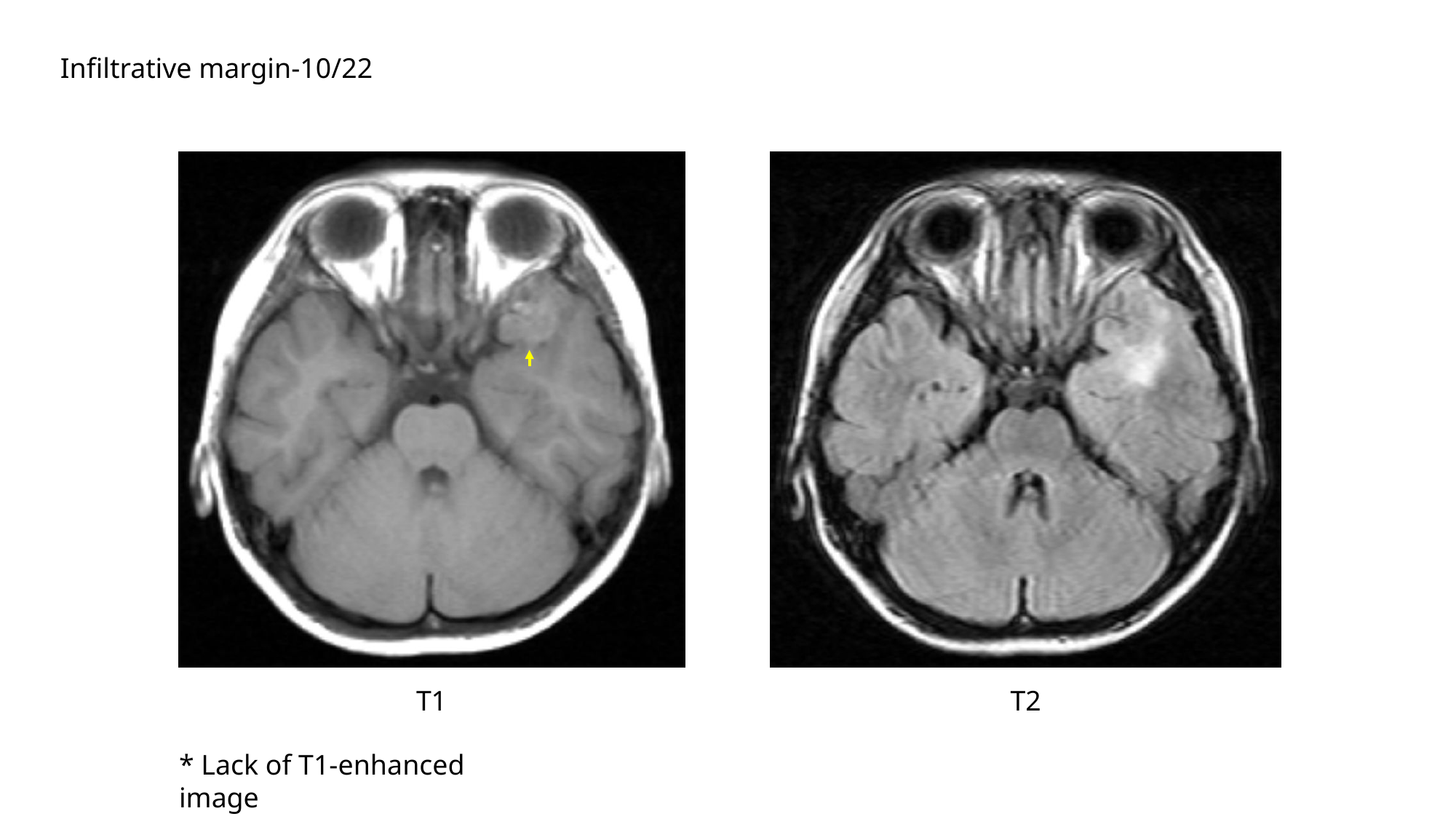

Infiltrative margin-10/22
T1
T2
* Lack of T1-enhanced image

## Slide 11
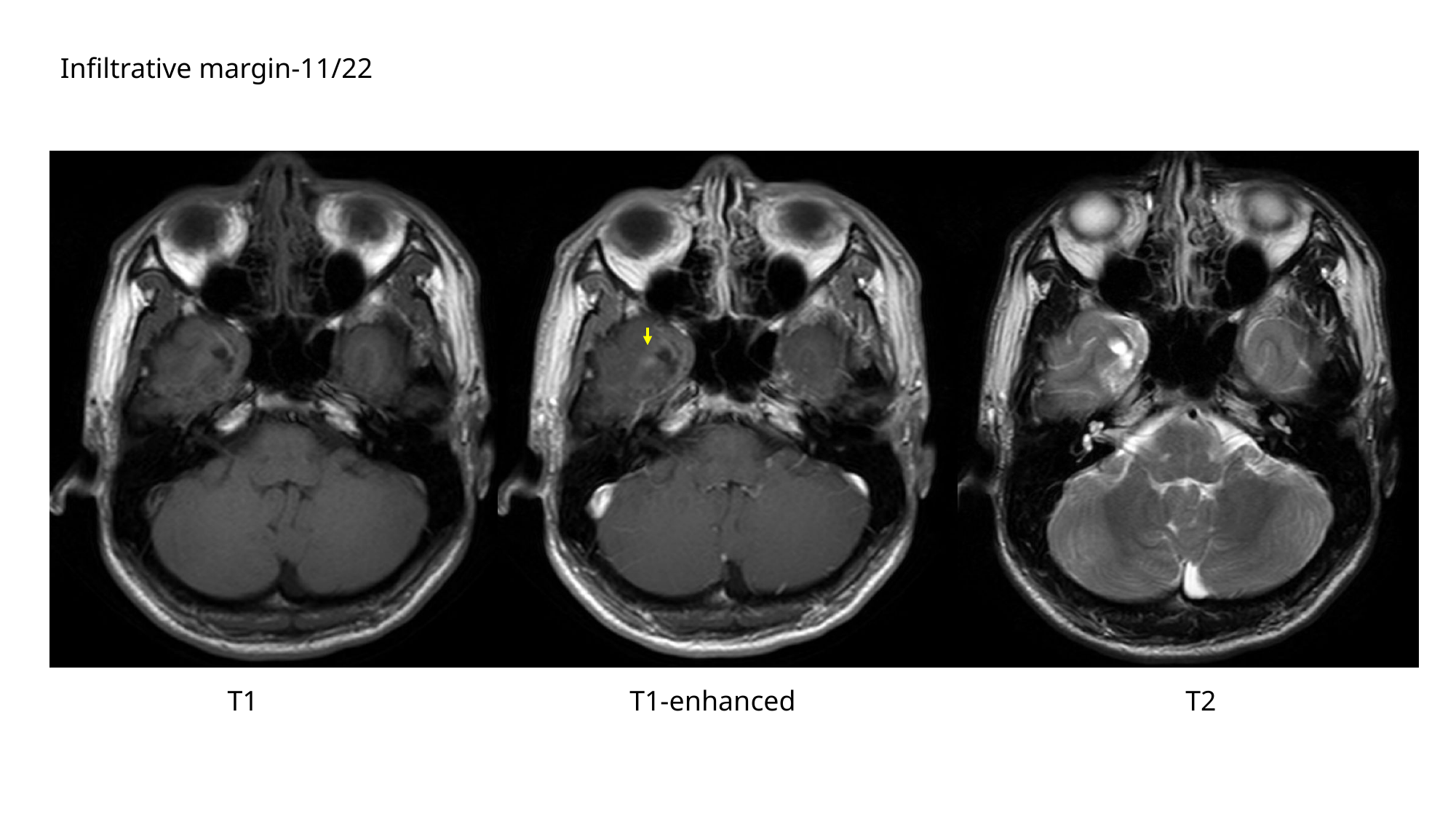

Infiltrative margin-11/22
T1
T1-enhanced
T2

## Slide 12
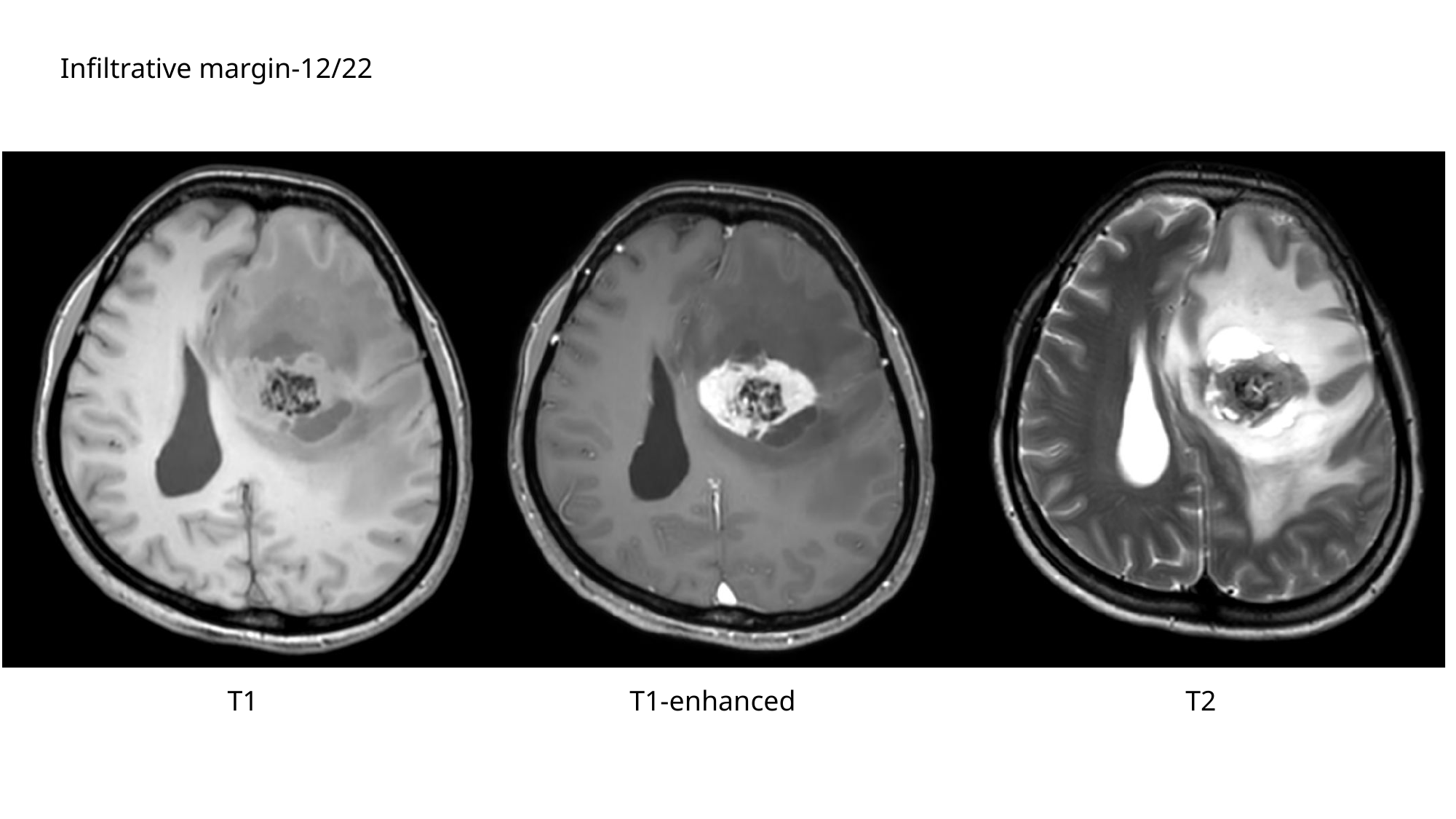

Infiltrative margin-12/22
T1
T1-enhanced
T2

## Slide 13
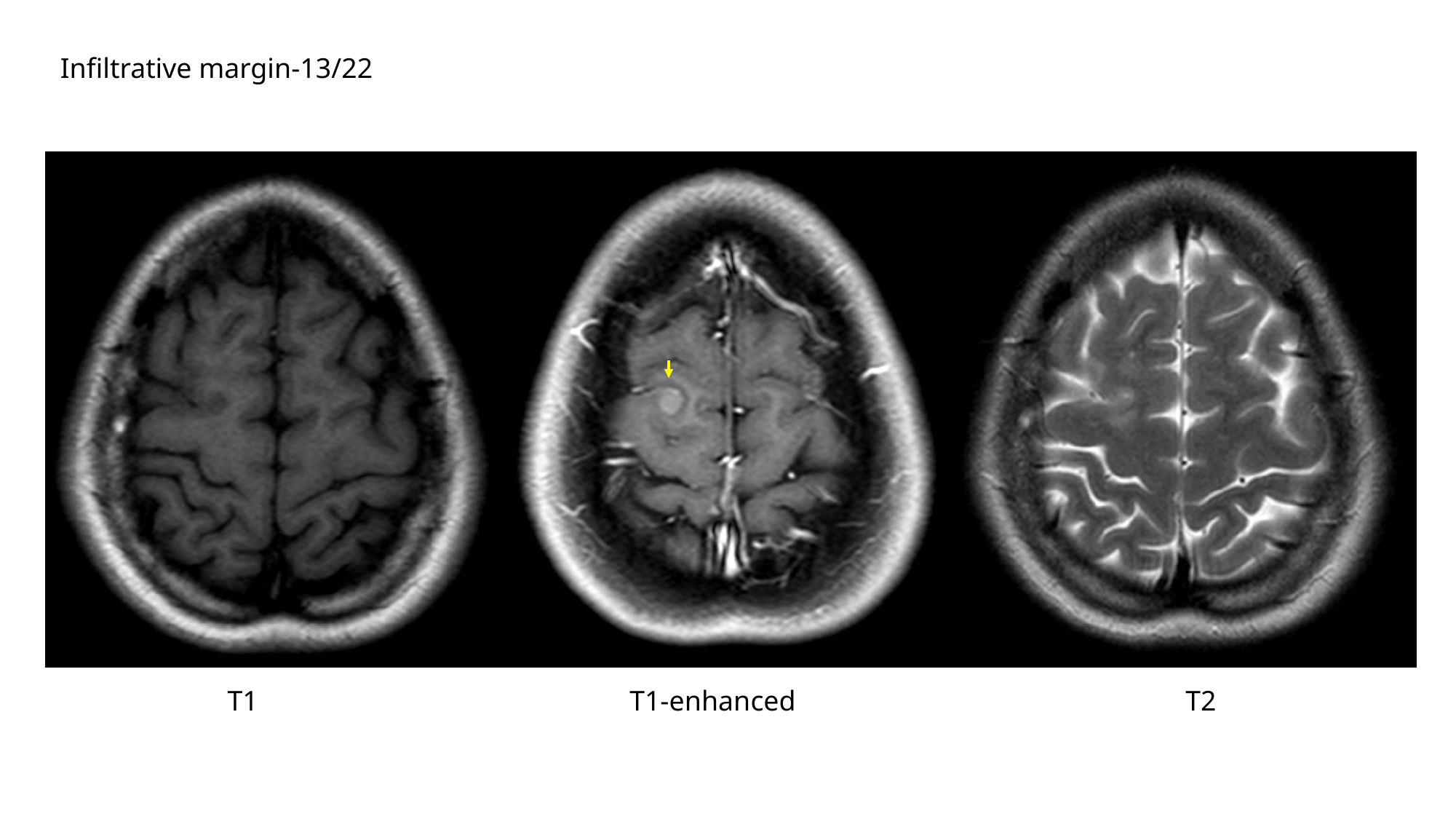

Infiltrative margin-13/22
20305646
T1
T1-enhanced
T2

## Slide 14
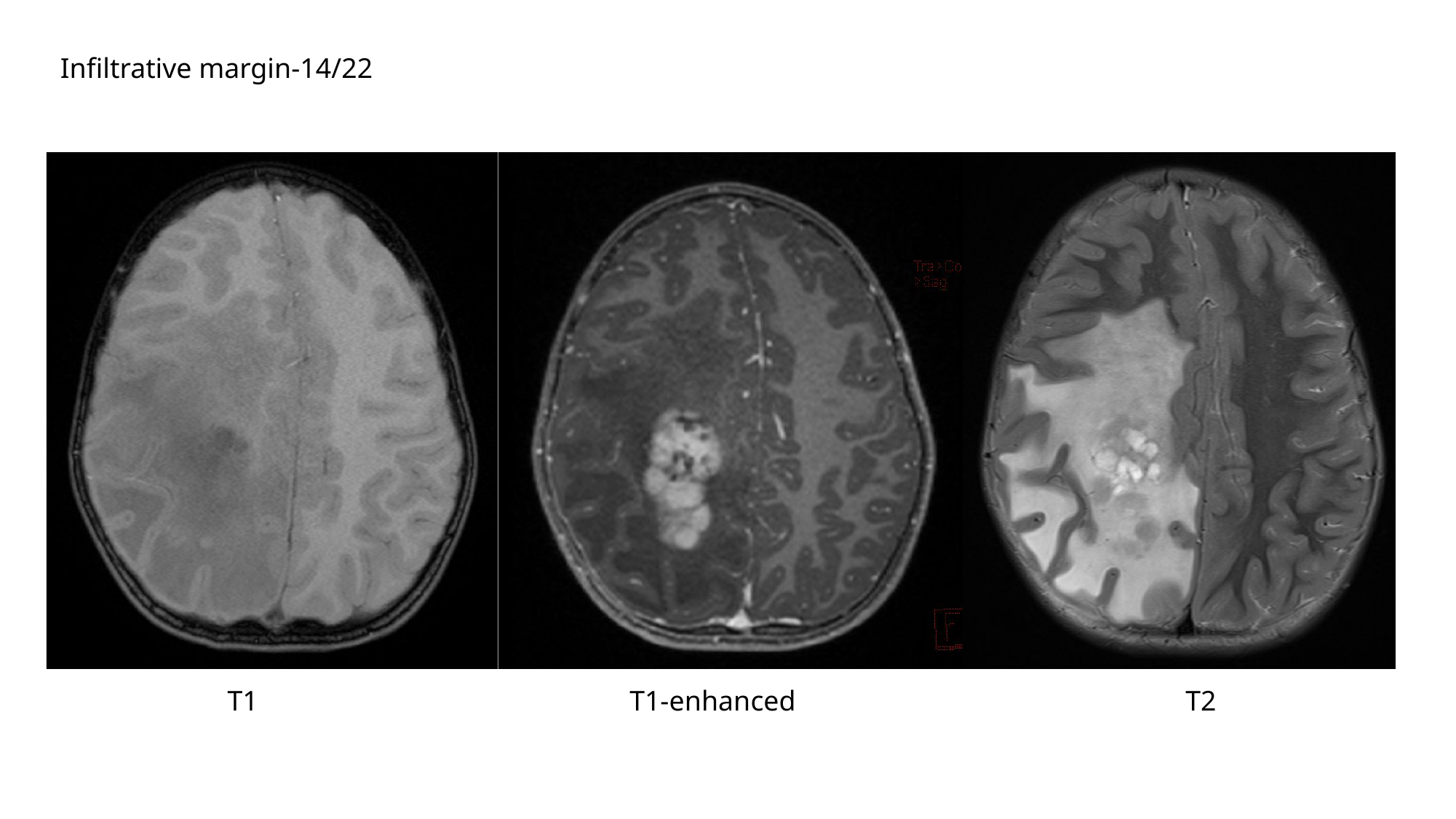

Infiltrative margin-14/22
T1
T1-enhanced
T2

## Slide 15
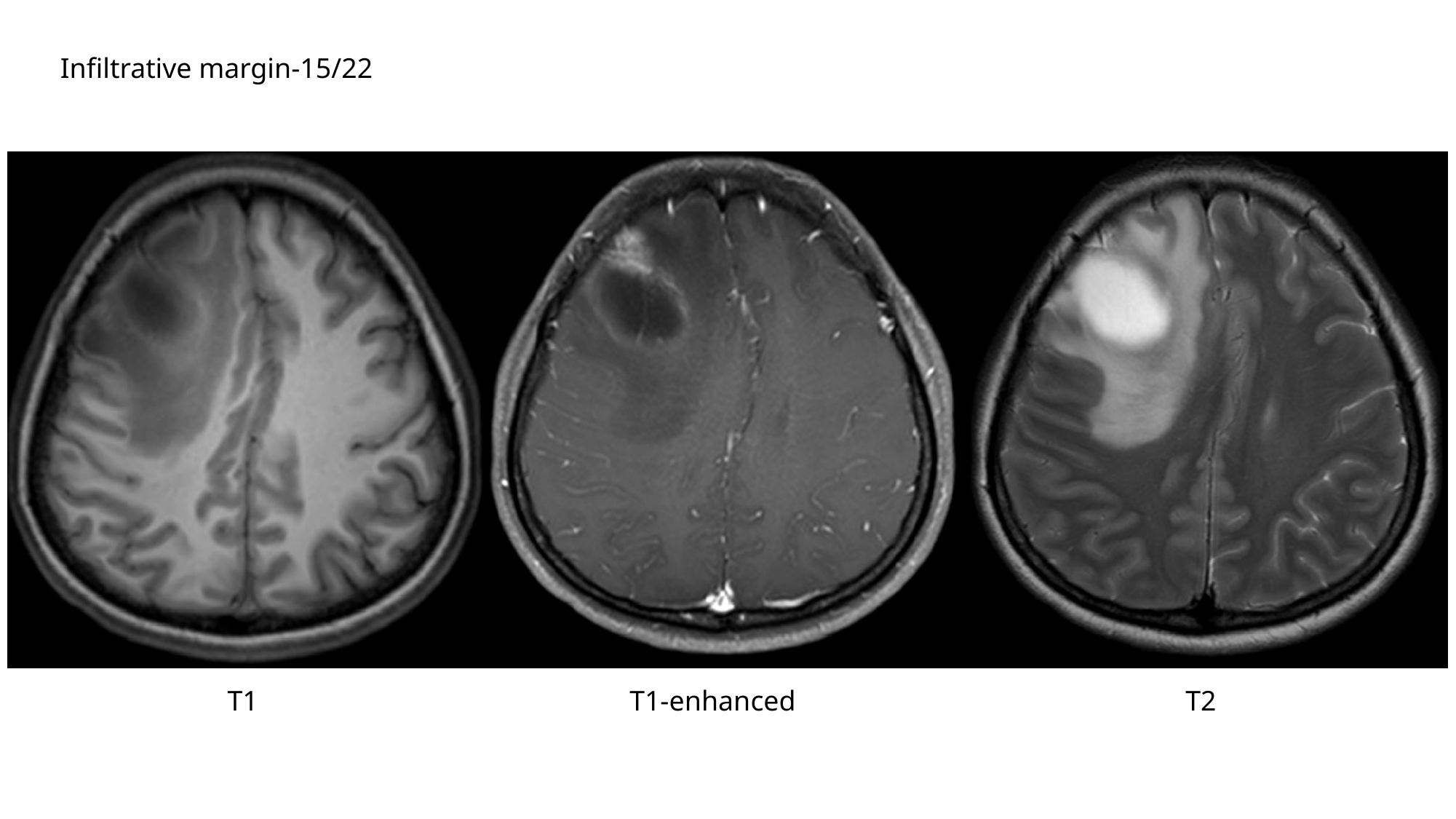

Infiltrative margin-15/22
T1
T1-enhanced
T2

## Slide 16
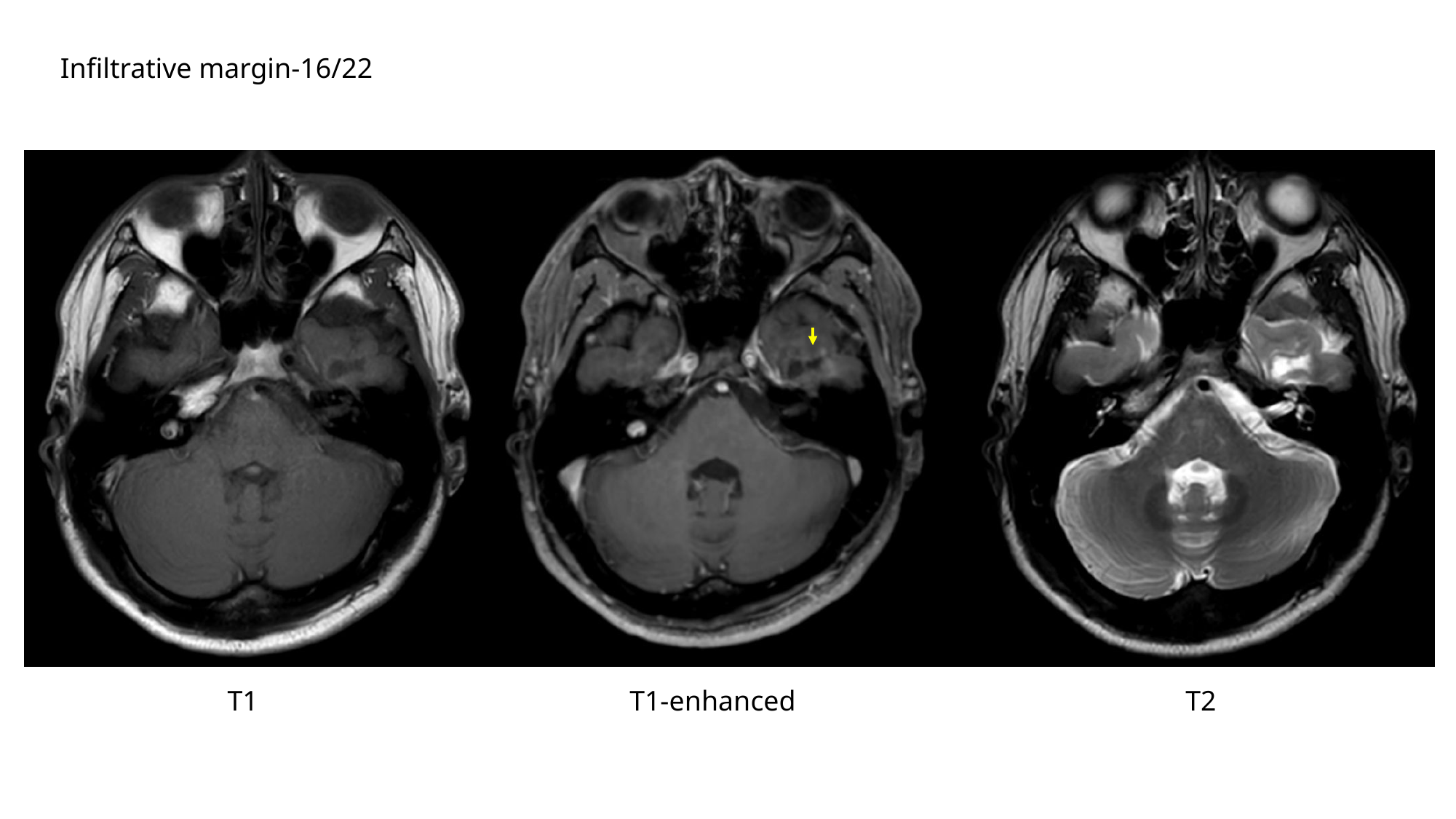

Infiltrative margin-16/22
T1
T1-enhanced
T2

## Slide 17
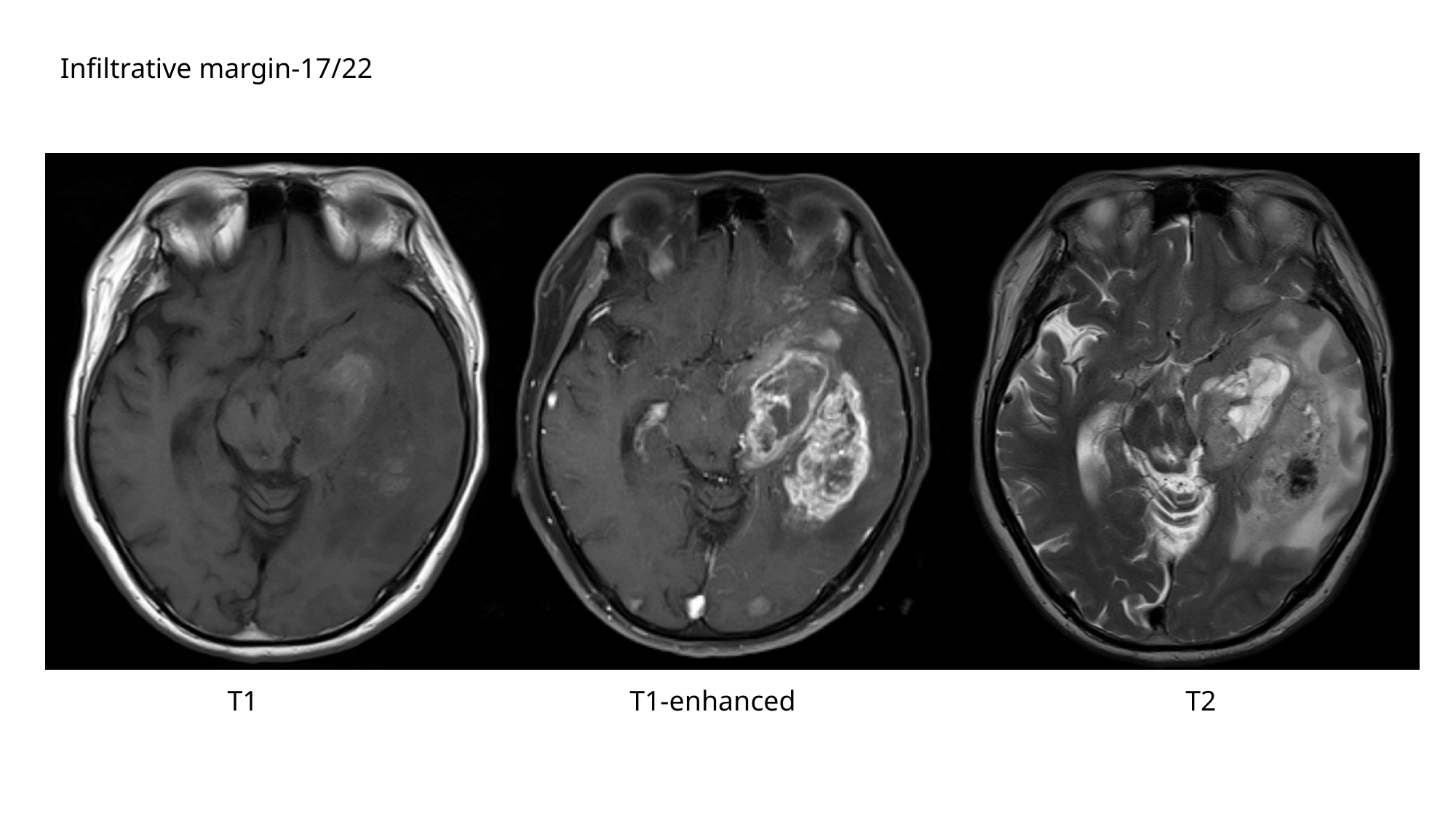

Infiltrative margin-17/22
T1
T1-enhanced
T2

## Slide 18
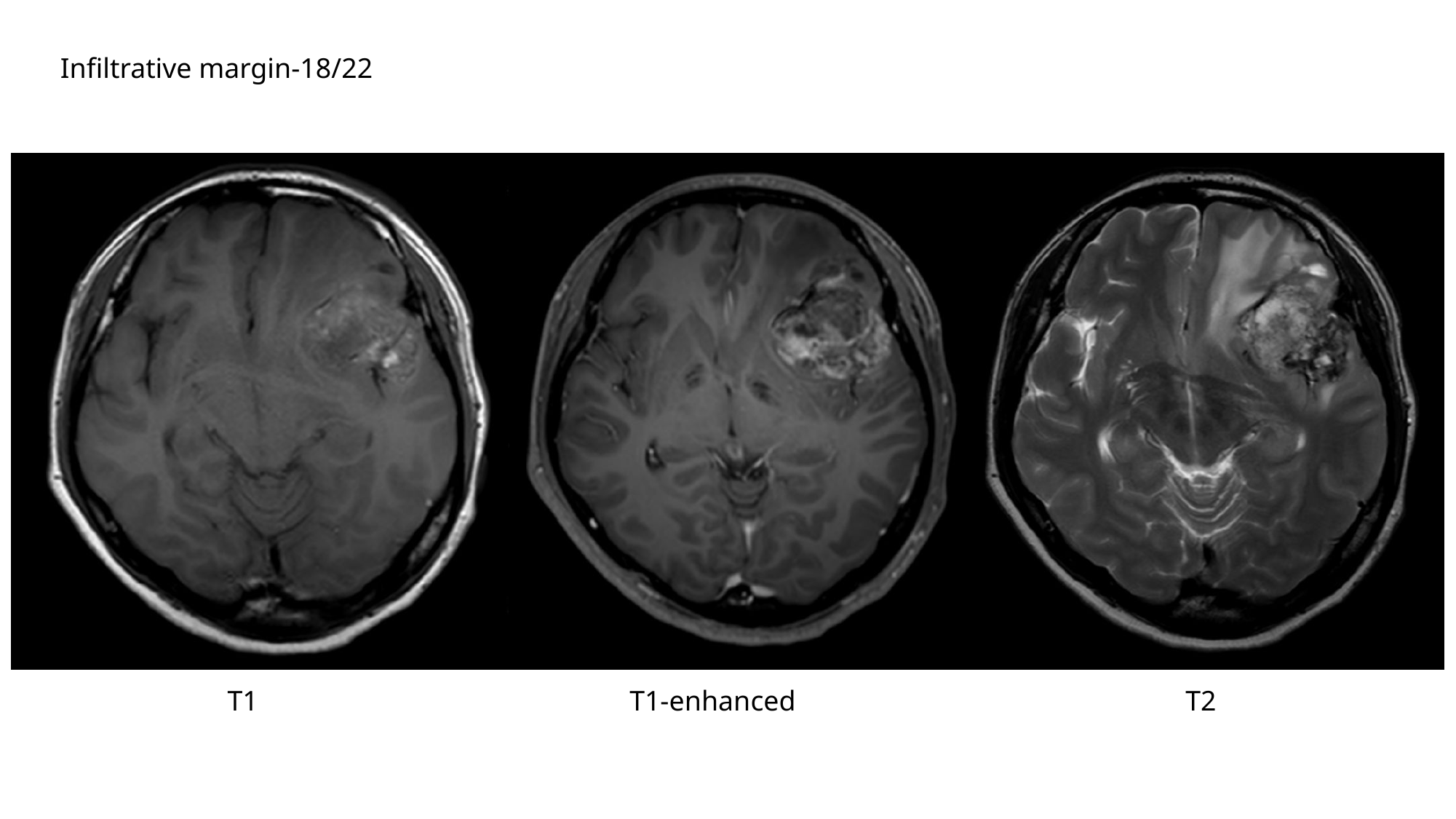

Infiltrative margin-18/22
T1
T1-enhanced
T2

## Slide 19
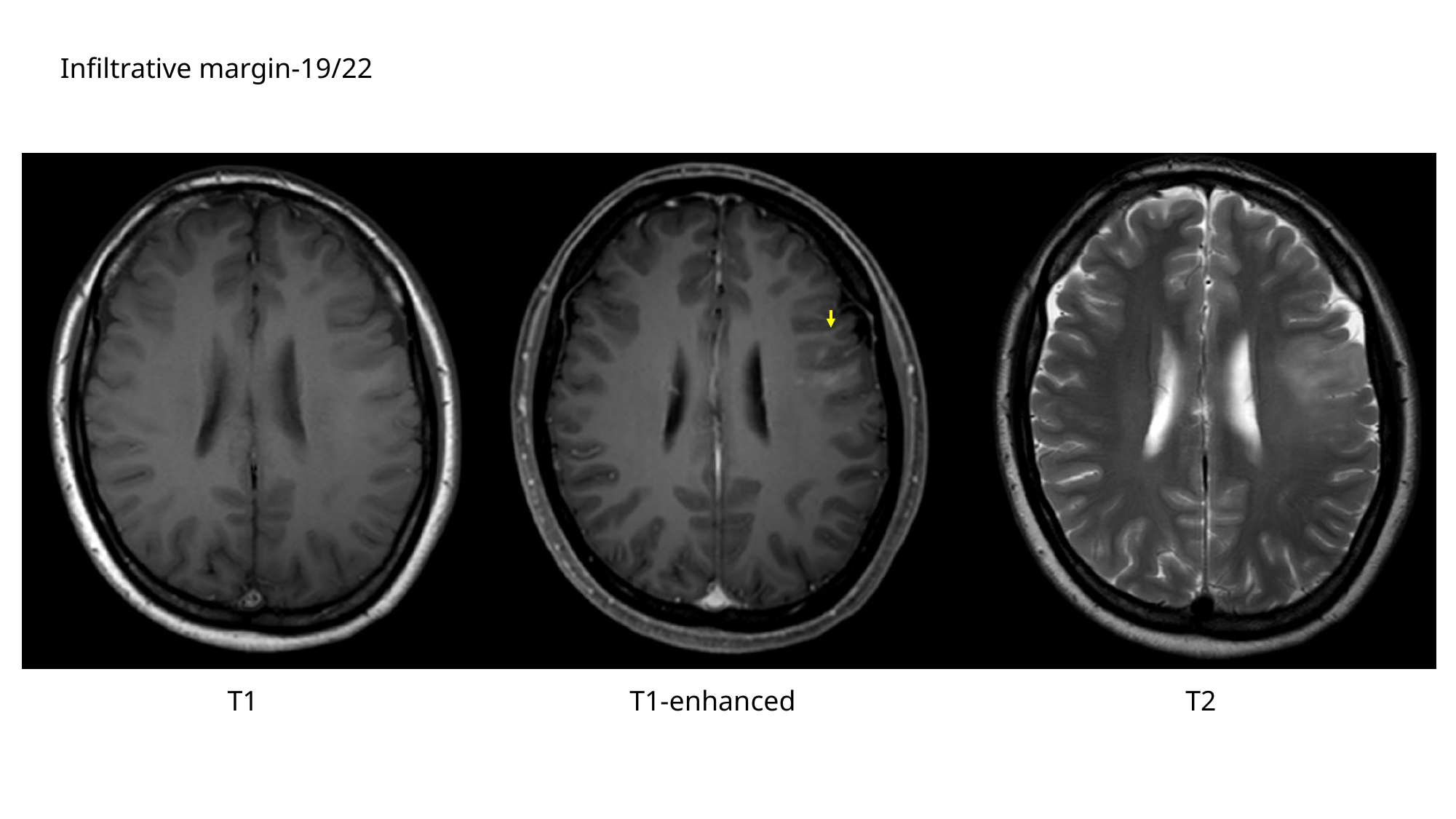

Infiltrative margin-19/22
T1
T1-enhanced
T2

## Slide 20
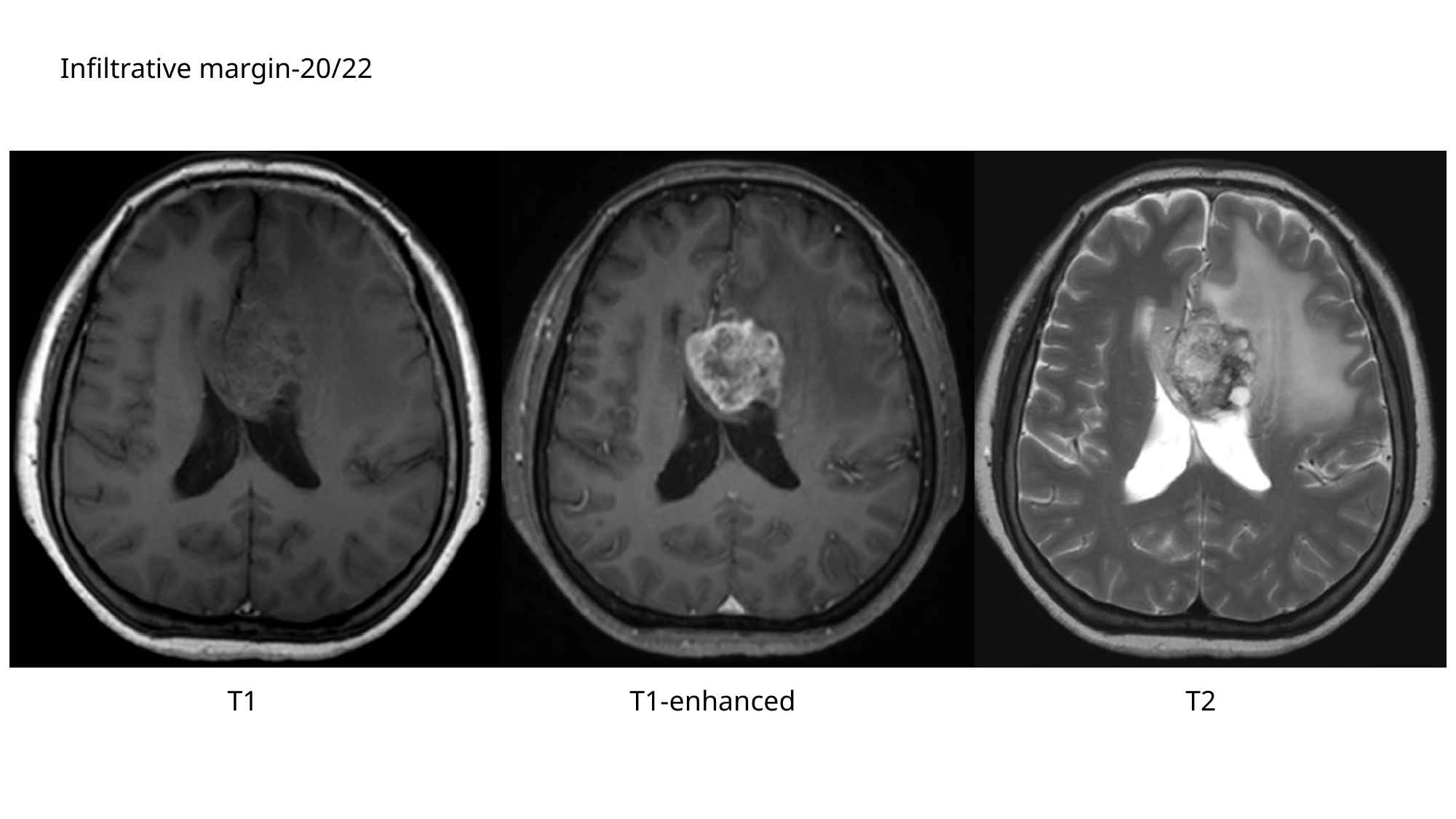

Infiltrative margin-20/22
T1
T1-enhanced
T2

## Slide 21
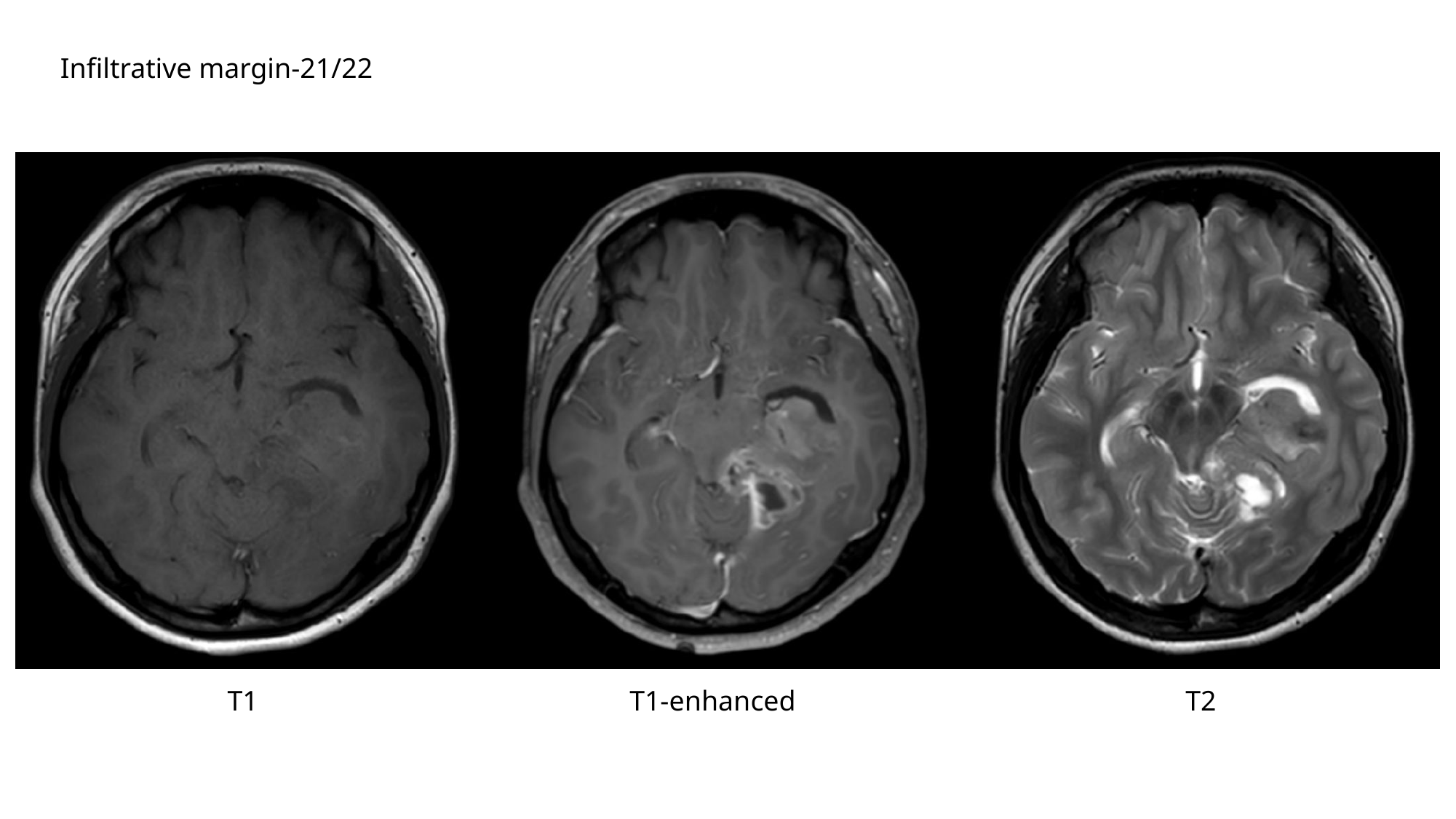

Infiltrative margin-21/22
T1
T1-enhanced
T2

## Slide 22
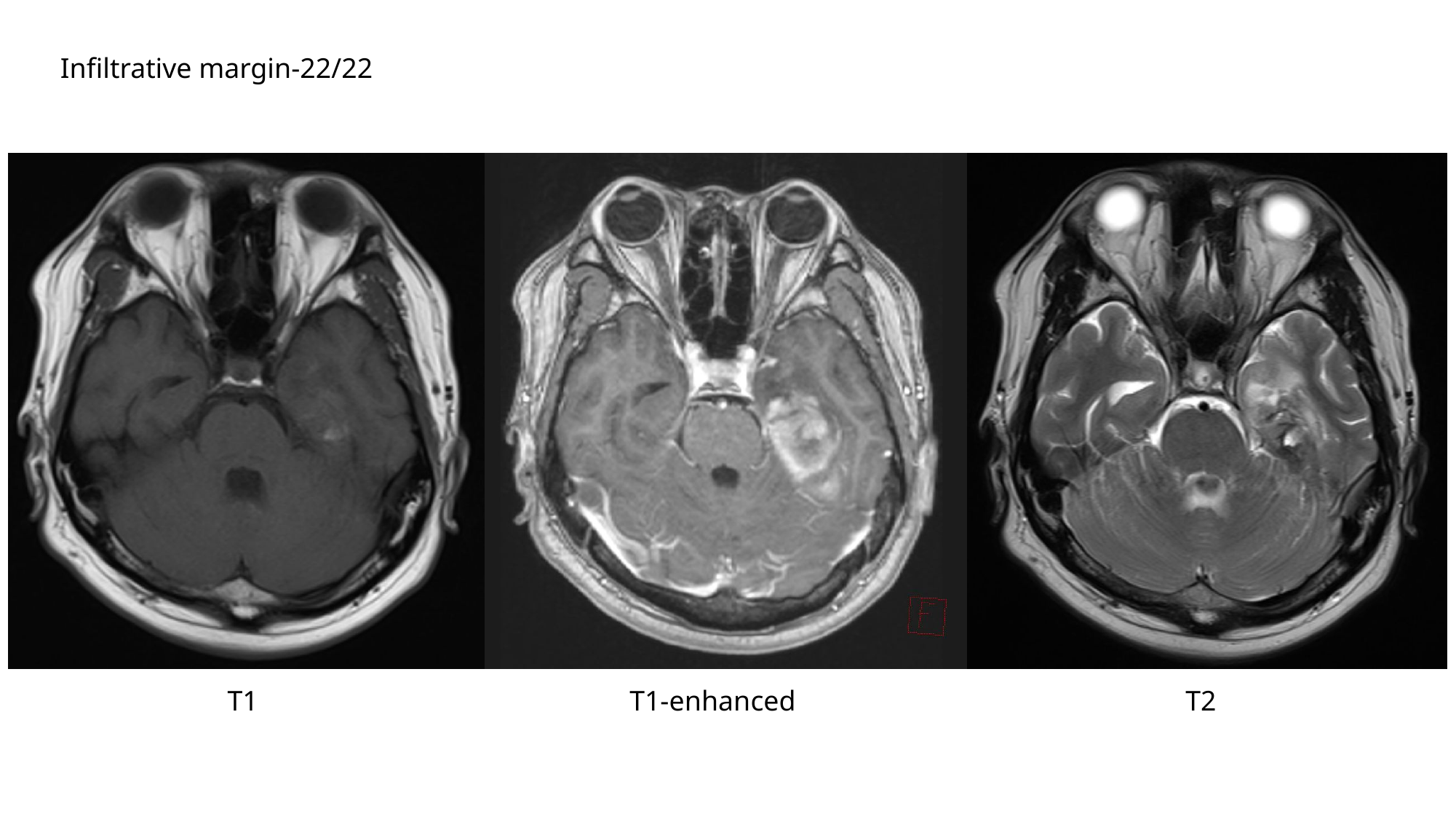

Infiltrative margin-22/22
T1
T1-enhanced
T2

## Slide 23
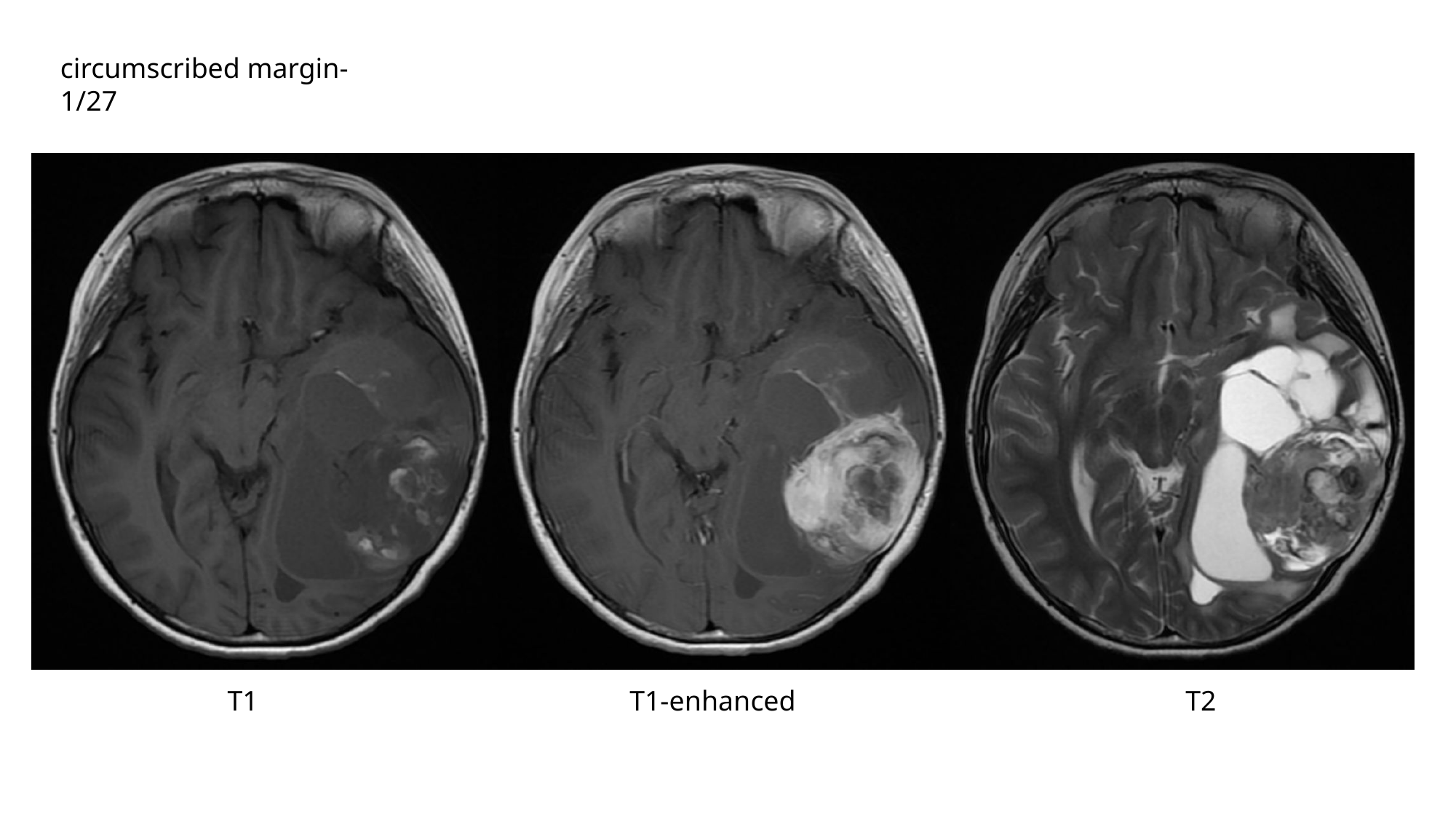

circumscribed margin-1/27
T1
T1-enhanced
T2

## Slide 24
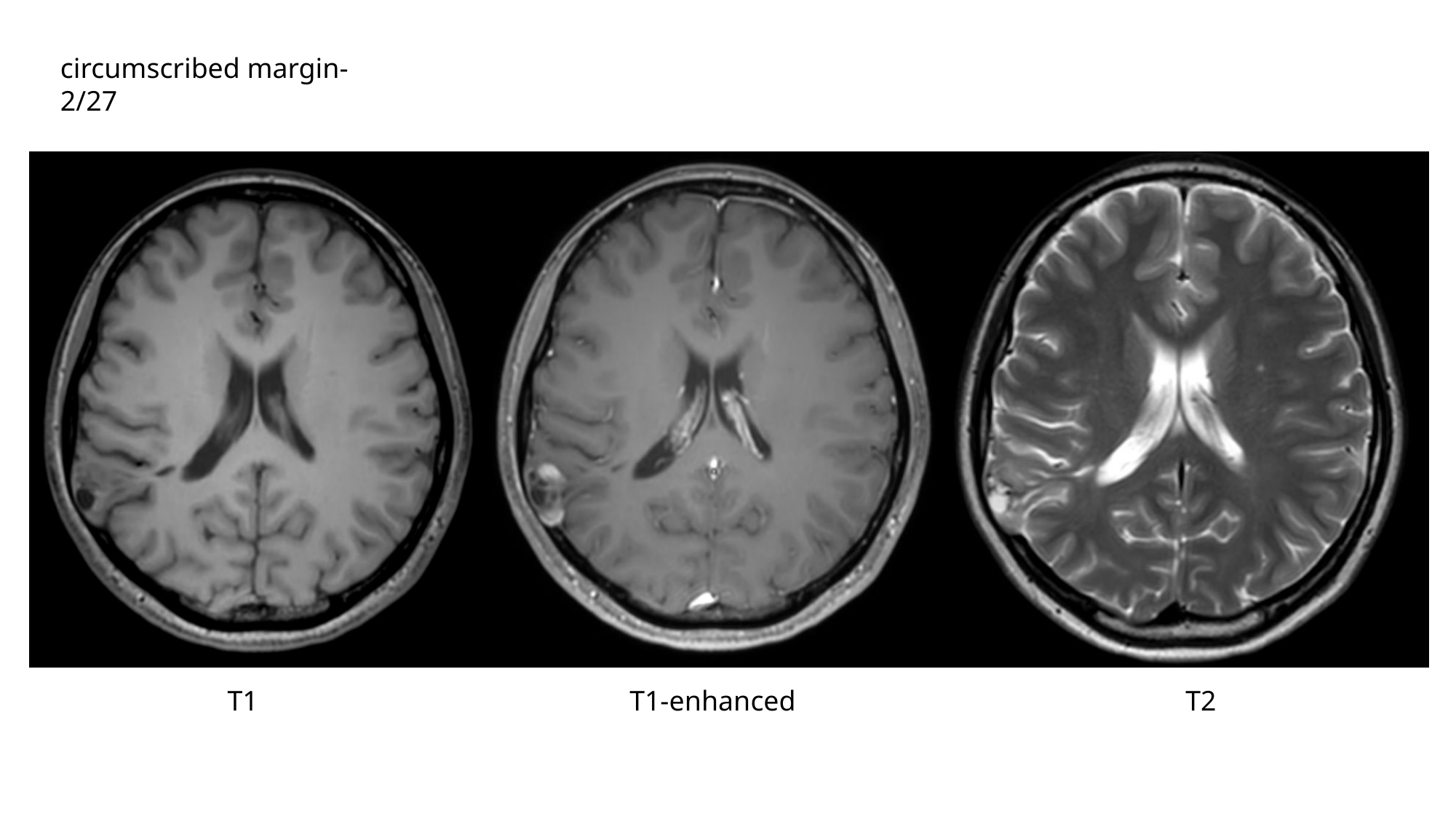

circumscribed margin-2/27
T1
T1-enhanced
T2

## Slide 25
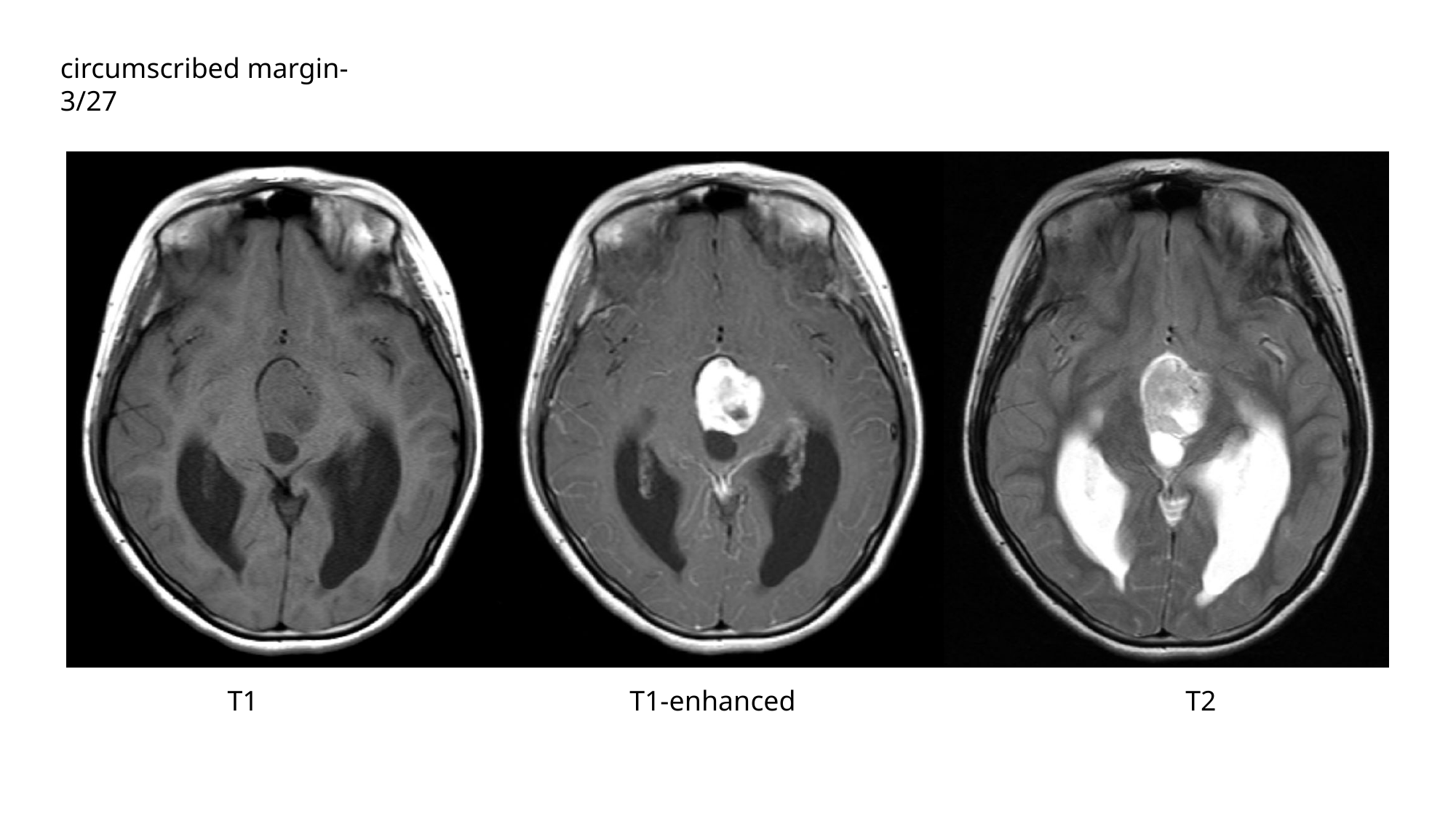

circumscribed margin-3/27
T1
T1-enhanced
T2

## Slide 26
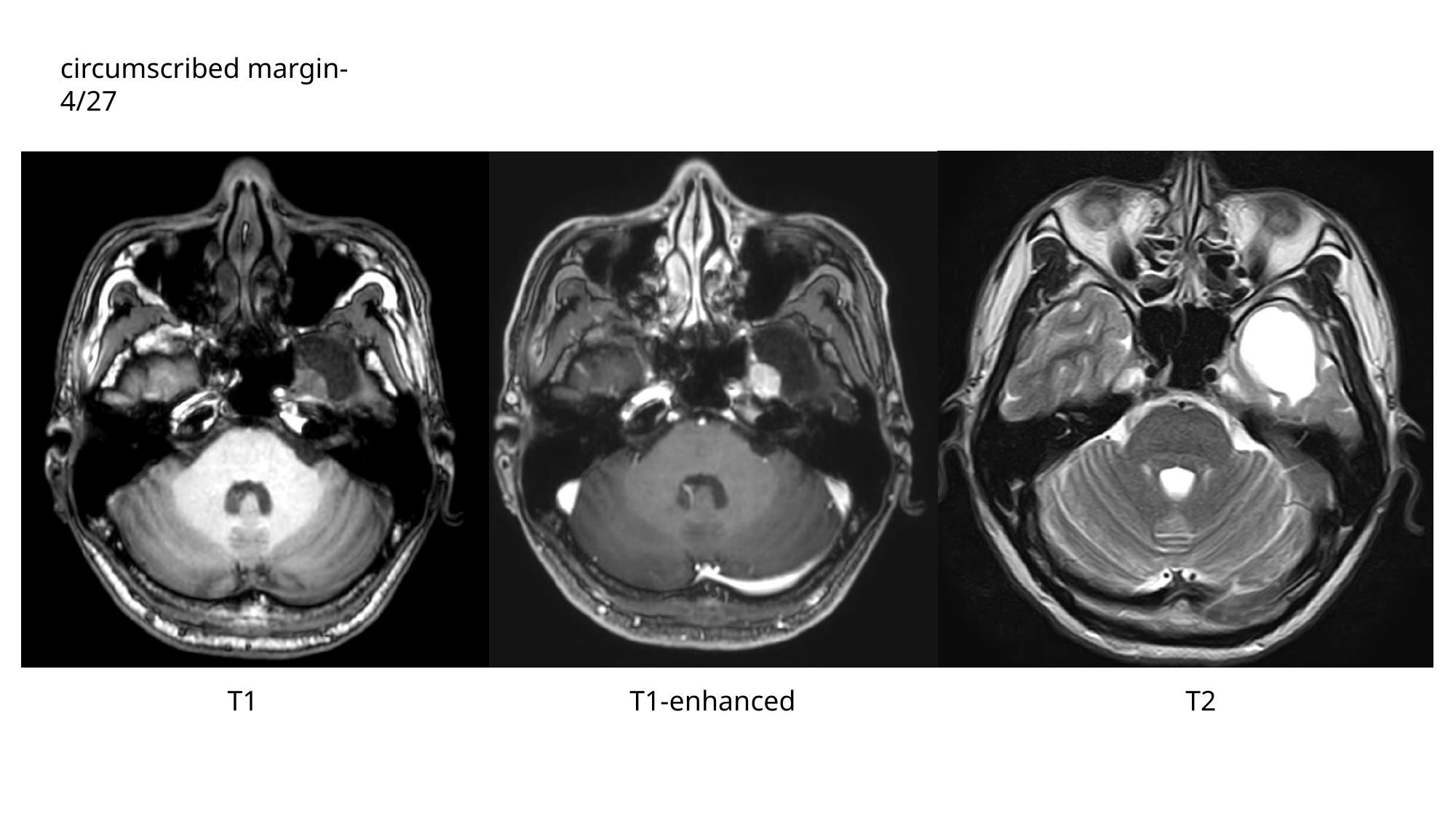

circumscribed margin-4/27
T1
T1-enhanced
T2

## Slide 27
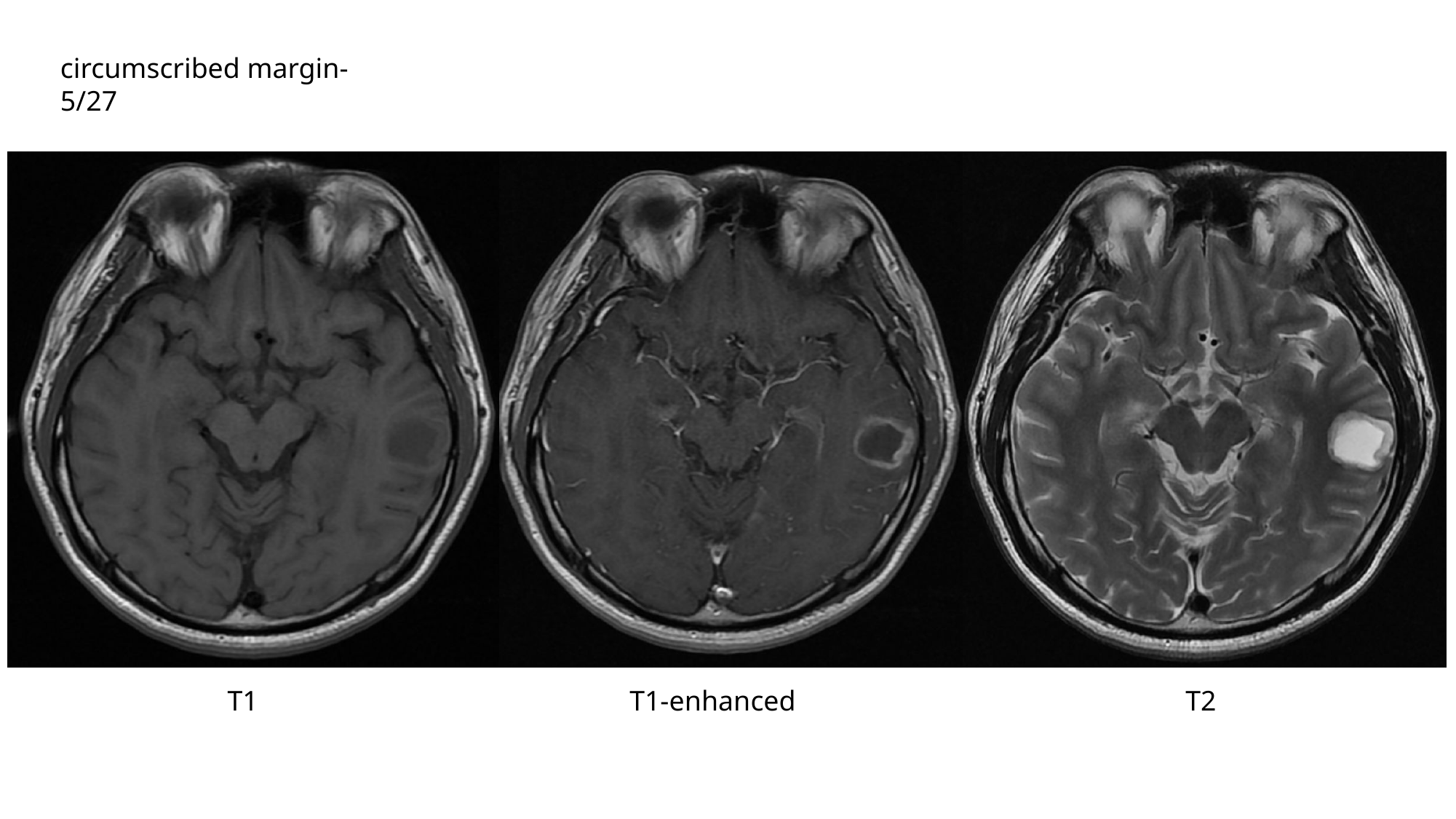

circumscribed margin-5/27
T1
T1-enhanced
T2

## Slide 28
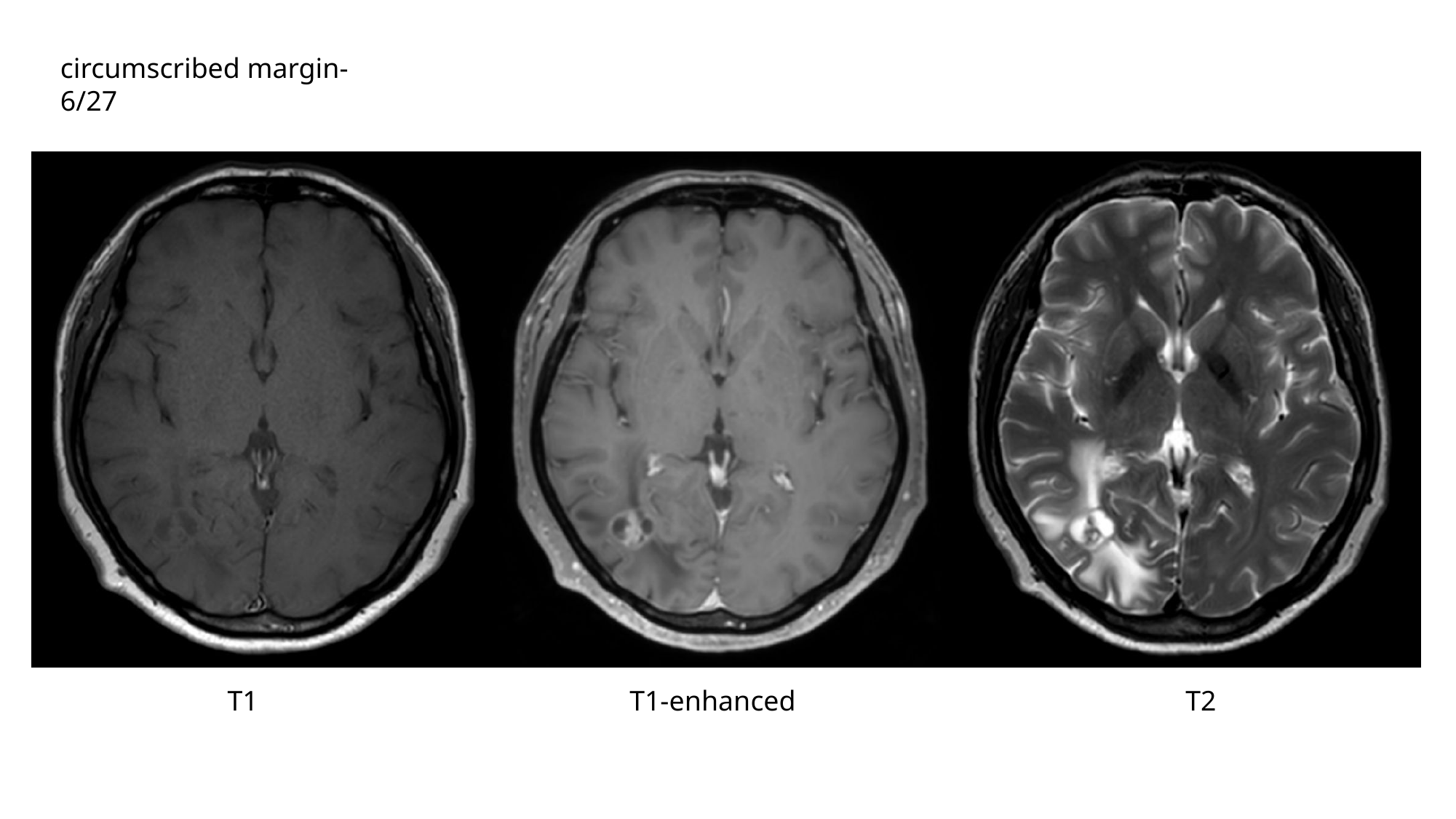

circumscribed margin-6/27
T1
T1-enhanced
T2

## Slide 29
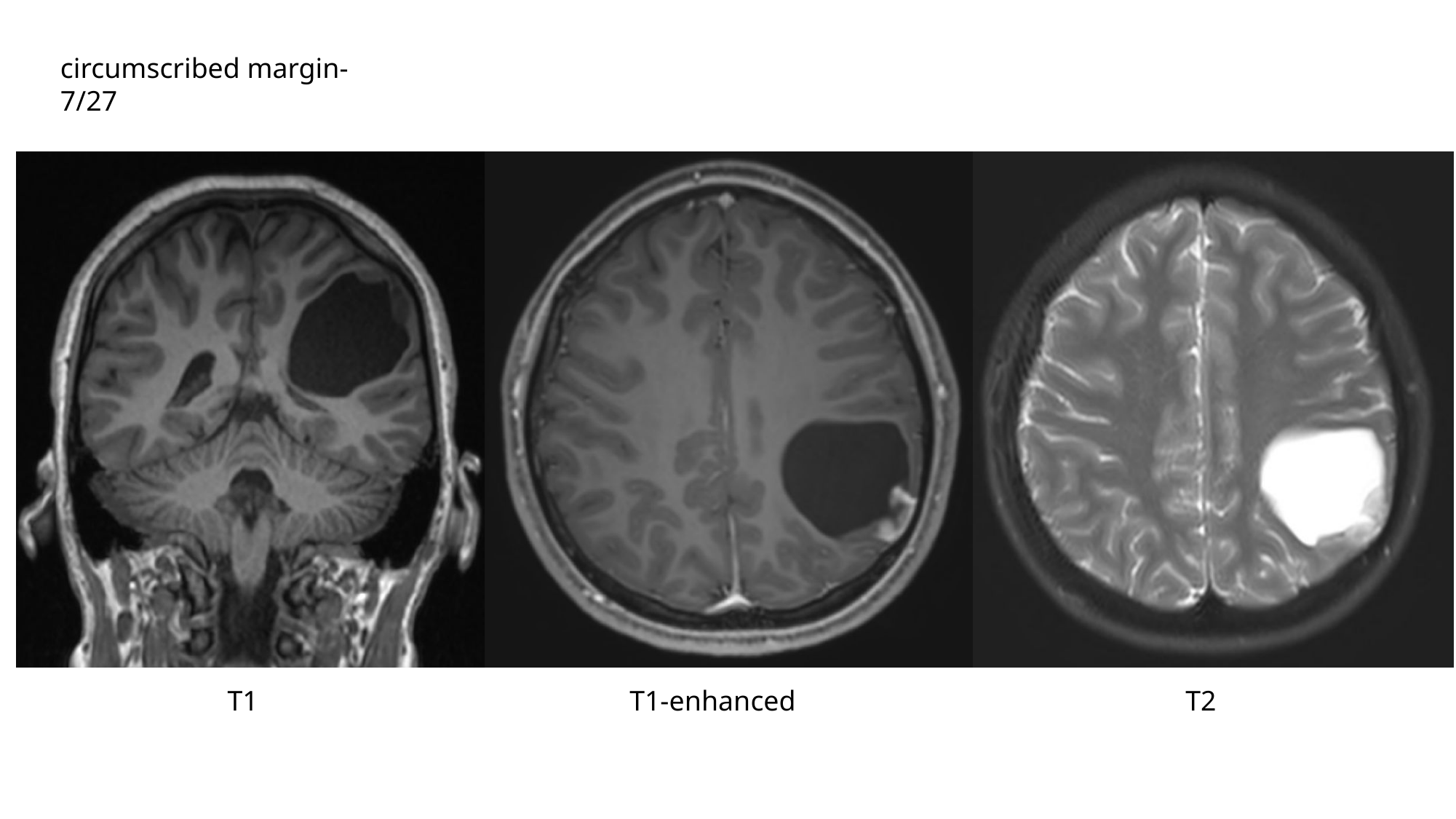

circumscribed margin-7/27
T1
T1-enhanced
T2

## Slide 30
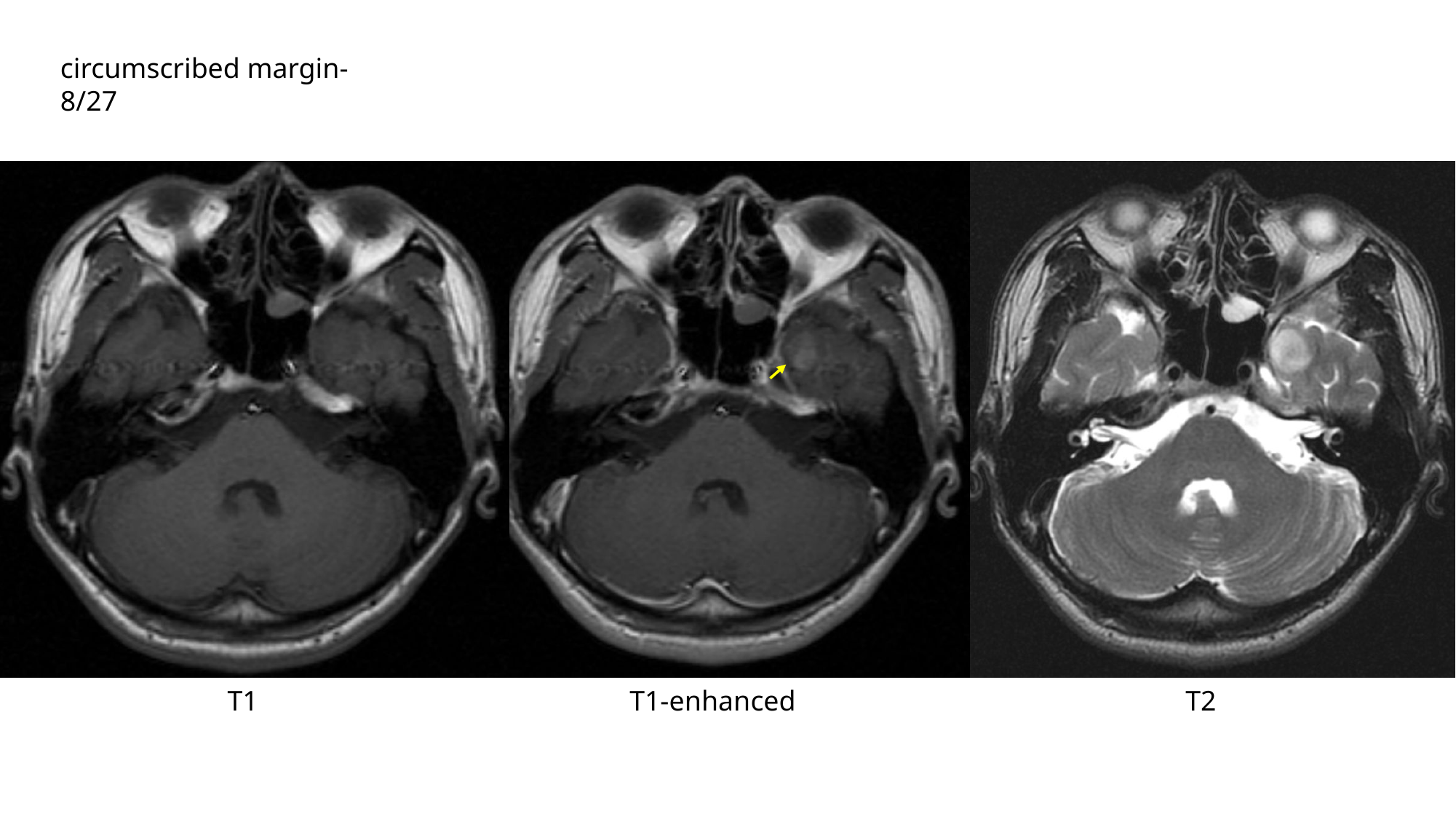

circumscribed margin-8/27
T1
T1-enhanced
T2

## Slide 31
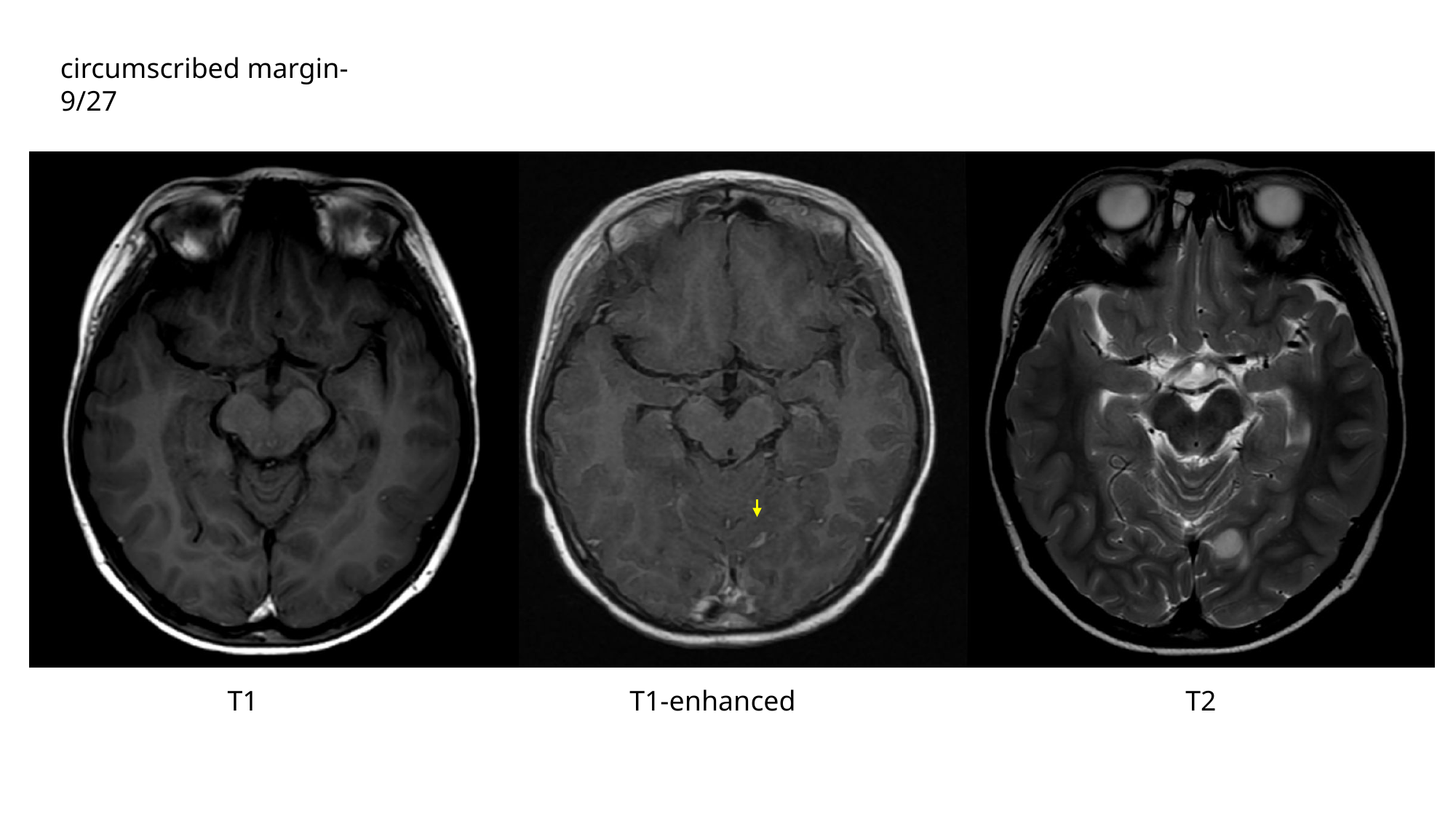

circumscribed margin-9/27
T1
T1-enhanced
T2

## Slide 32
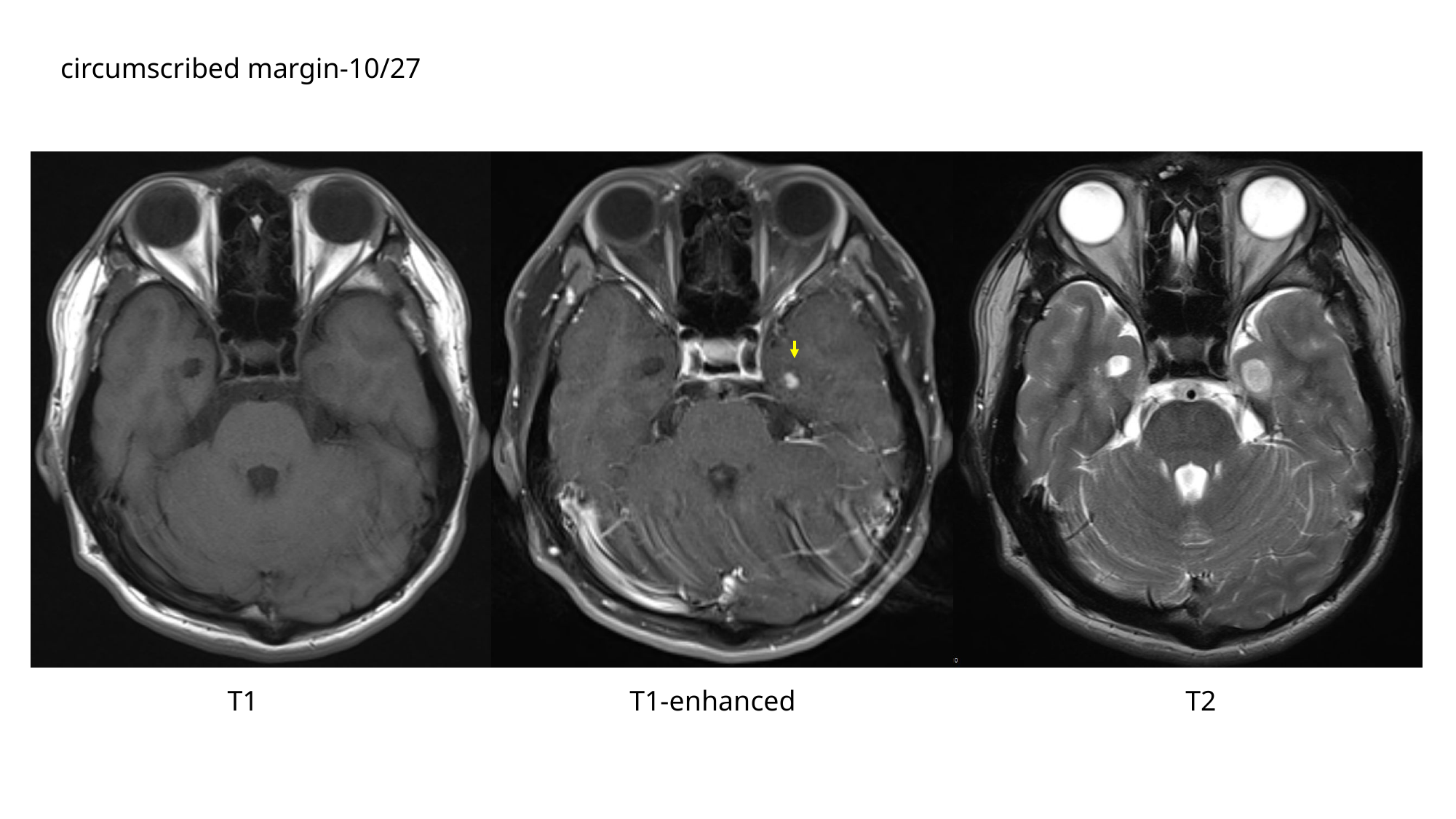

circumscribed margin-10/27
T1
T1-enhanced
T2

## Slide 33
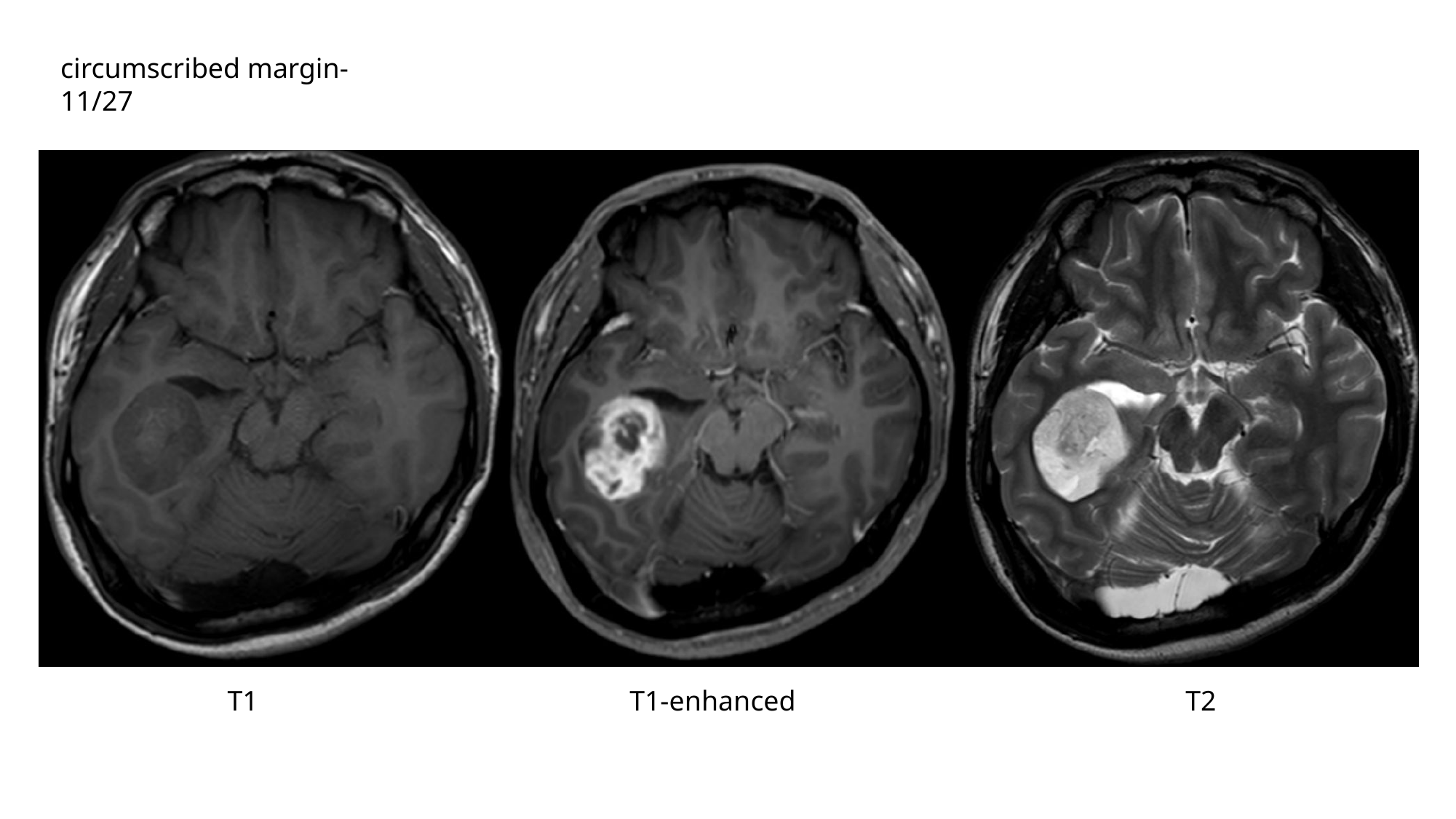

circumscribed margin-11/27
T1
T1-enhanced
T2

## Slide 34
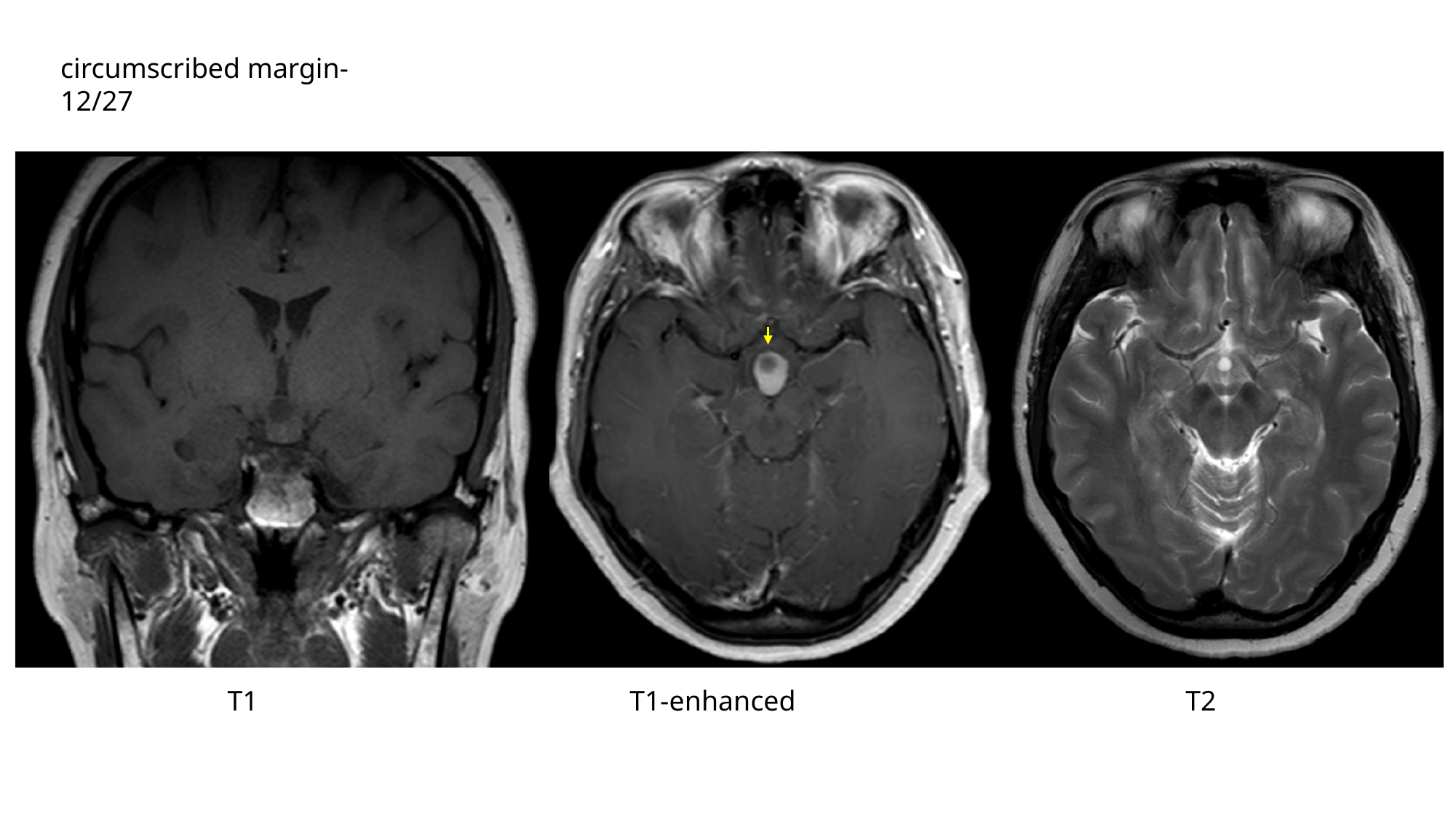

circumscribed margin-12/27
T1
T1-enhanced
T2

## Slide 35
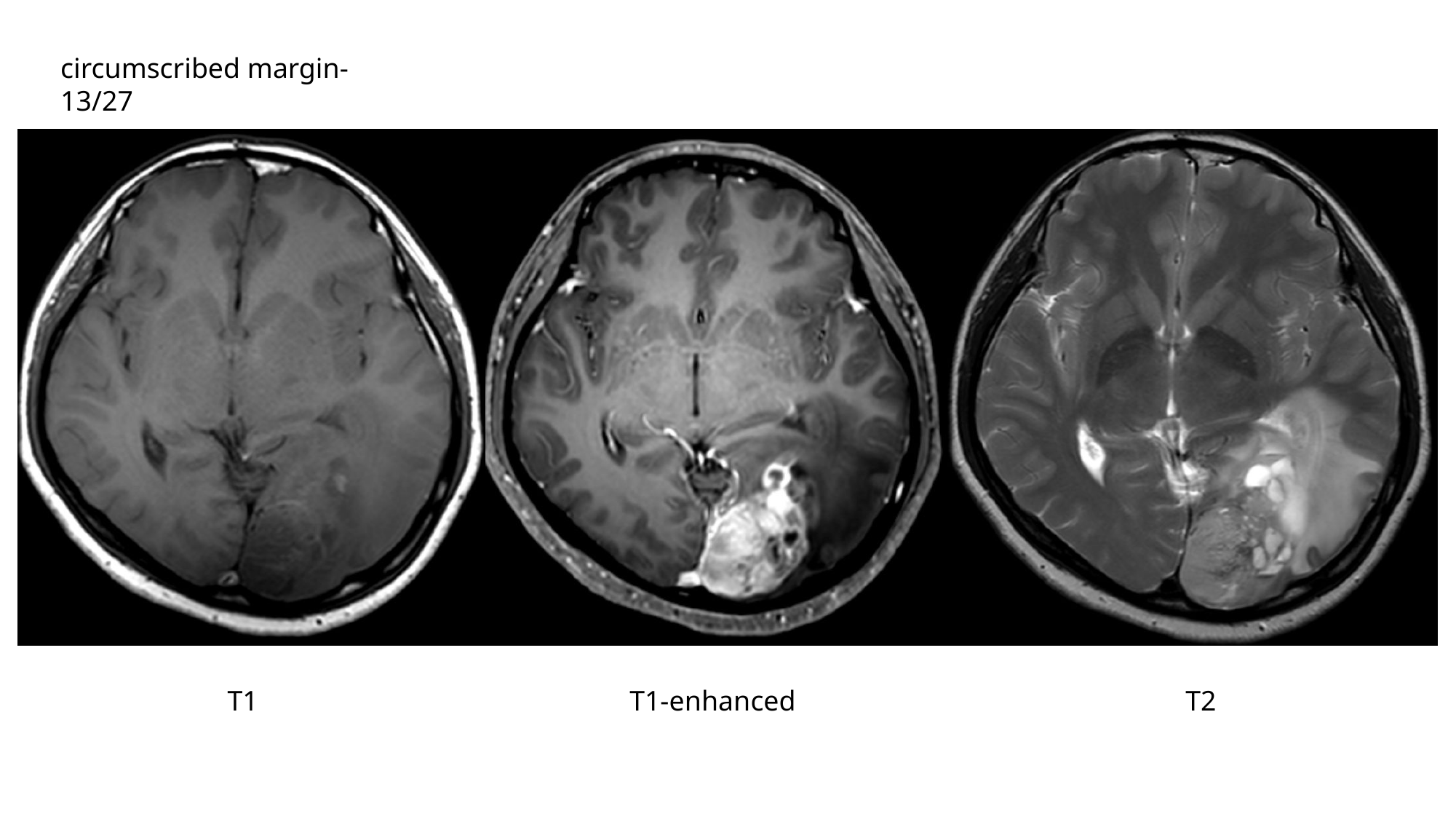

circumscribed margin-13/27
T1
T1-enhanced
T2

## Slide 36
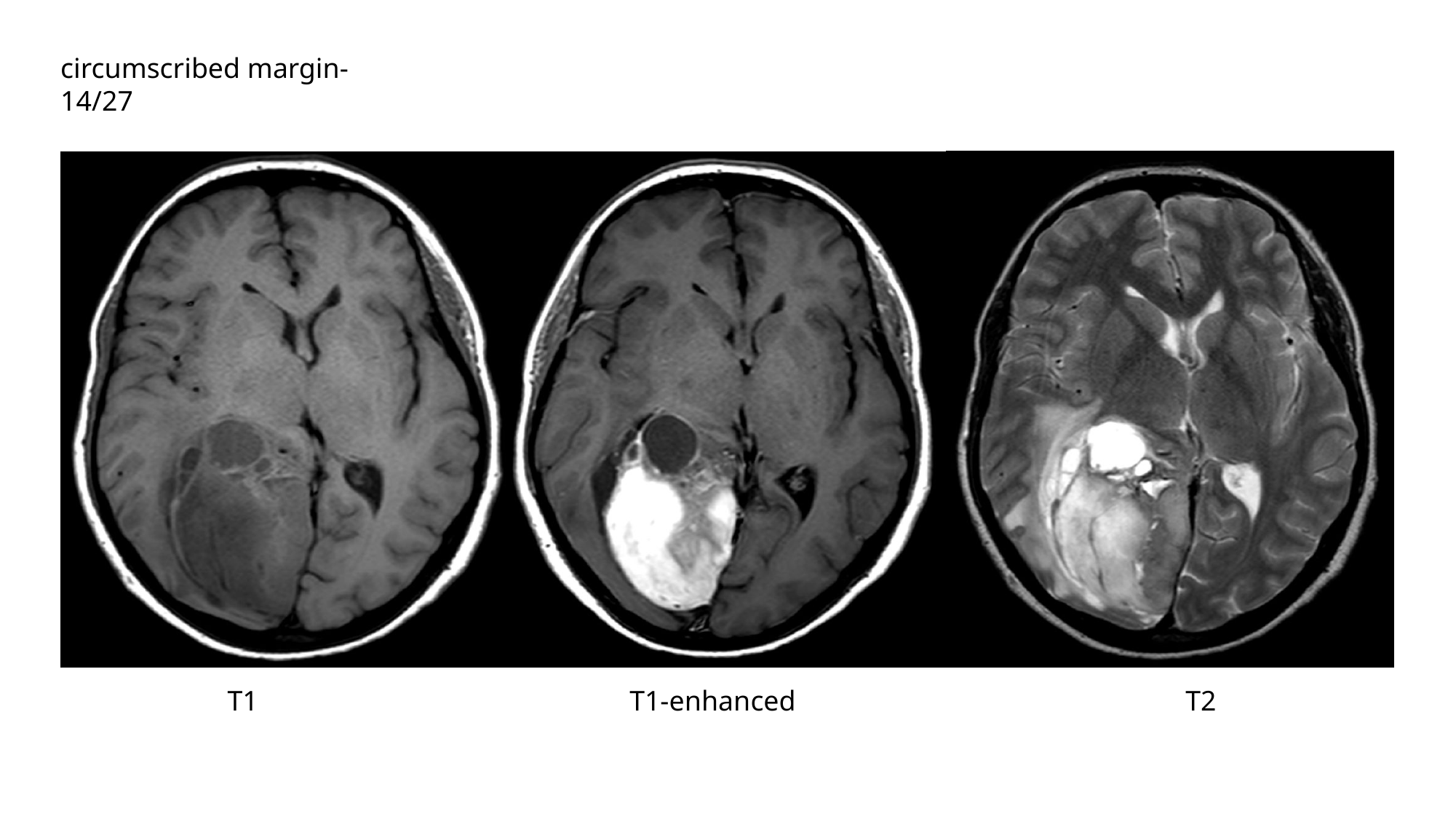

circumscribed margin-14/27
T1
T1-enhanced
T2

## Slide 37
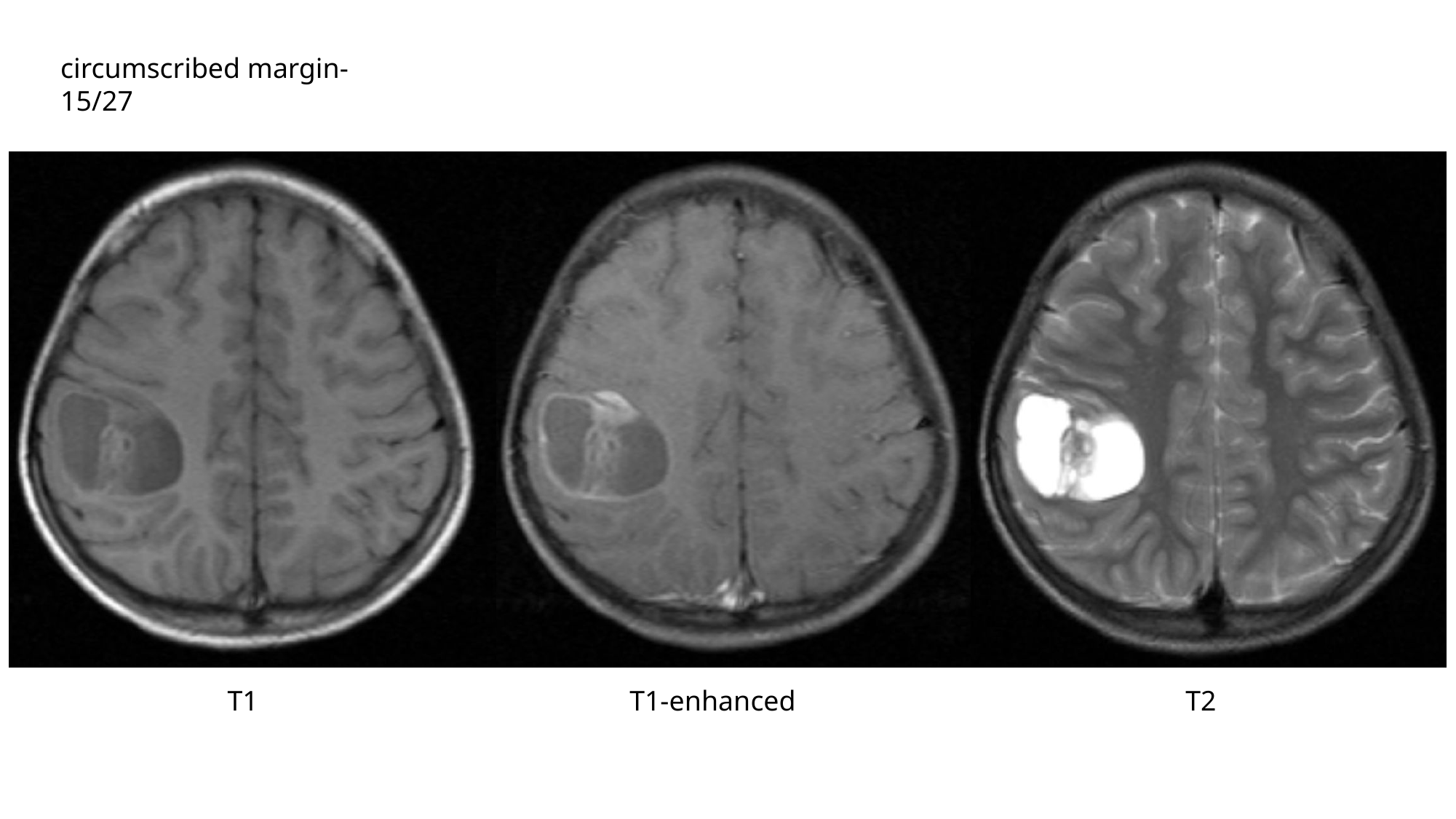

circumscribed margin-15/27
T1
T1-enhanced
T2

## Slide 38
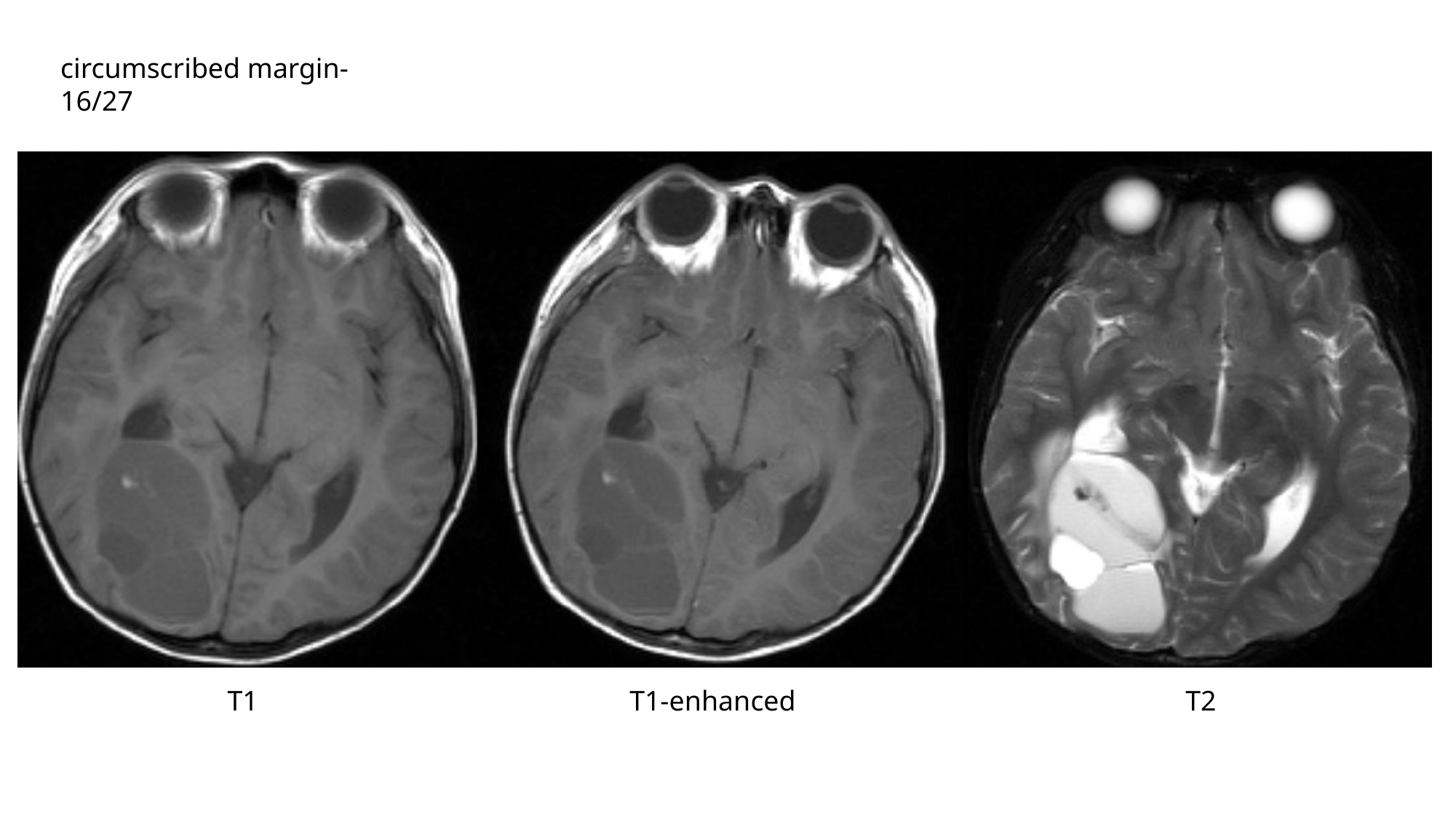

circumscribed margin-16/27
T1
T1-enhanced
T2

## Slide 39
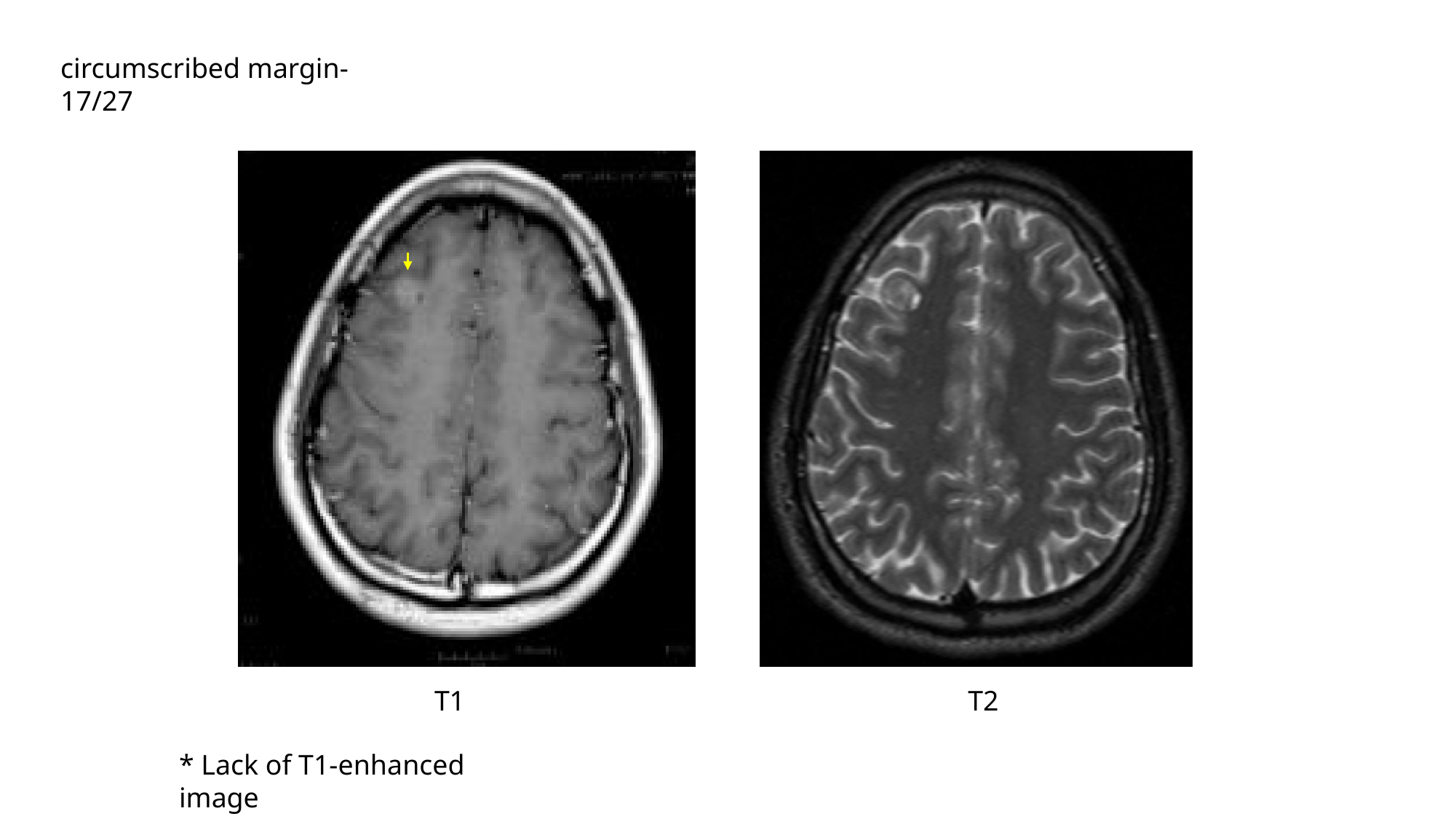

circumscribed margin-17/27
T1
T2
* Lack of T1-enhanced image

## Slide 40
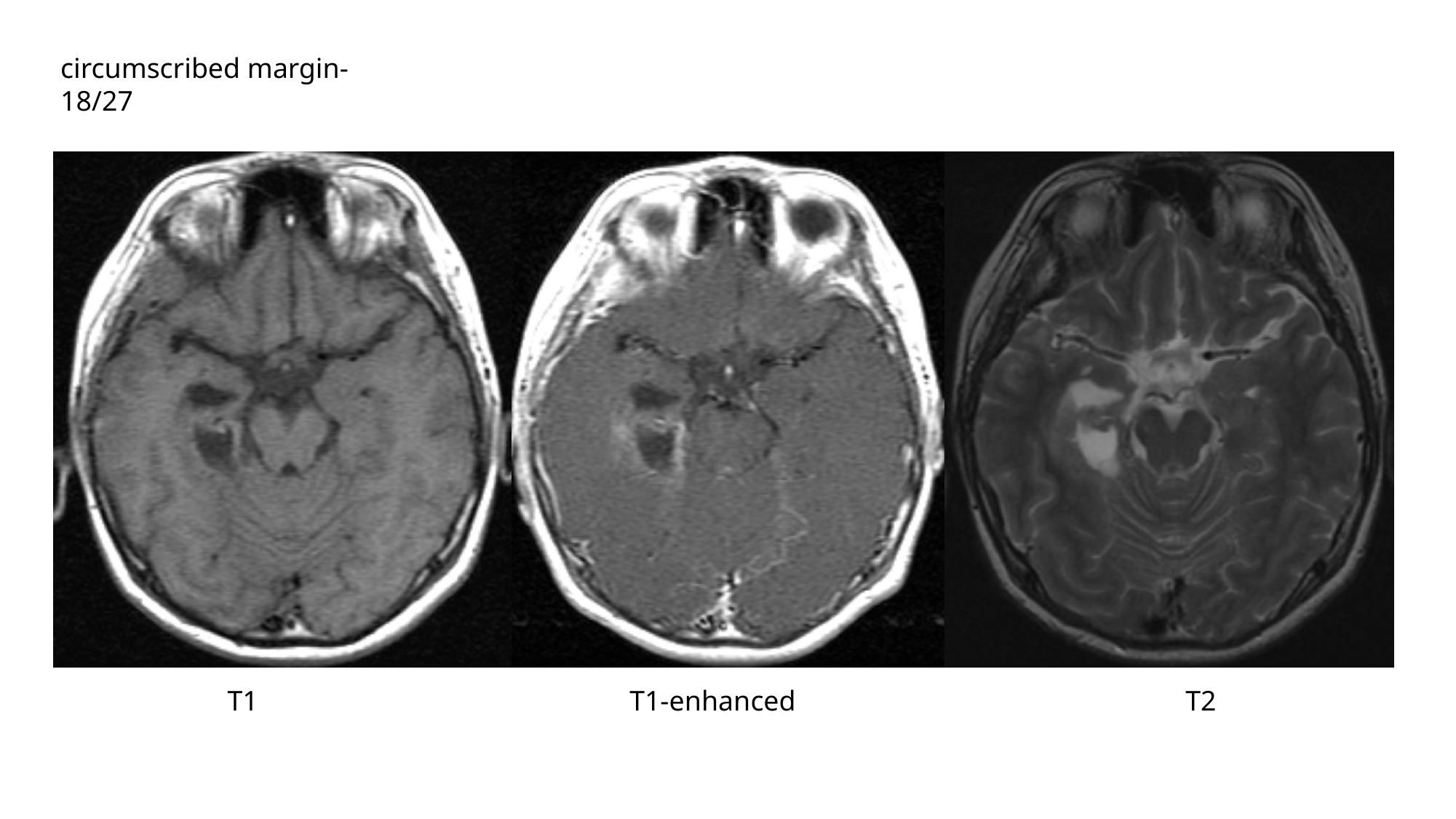

circumscribed margin-18/27
T1
T1-enhanced
T2

## Slide 41
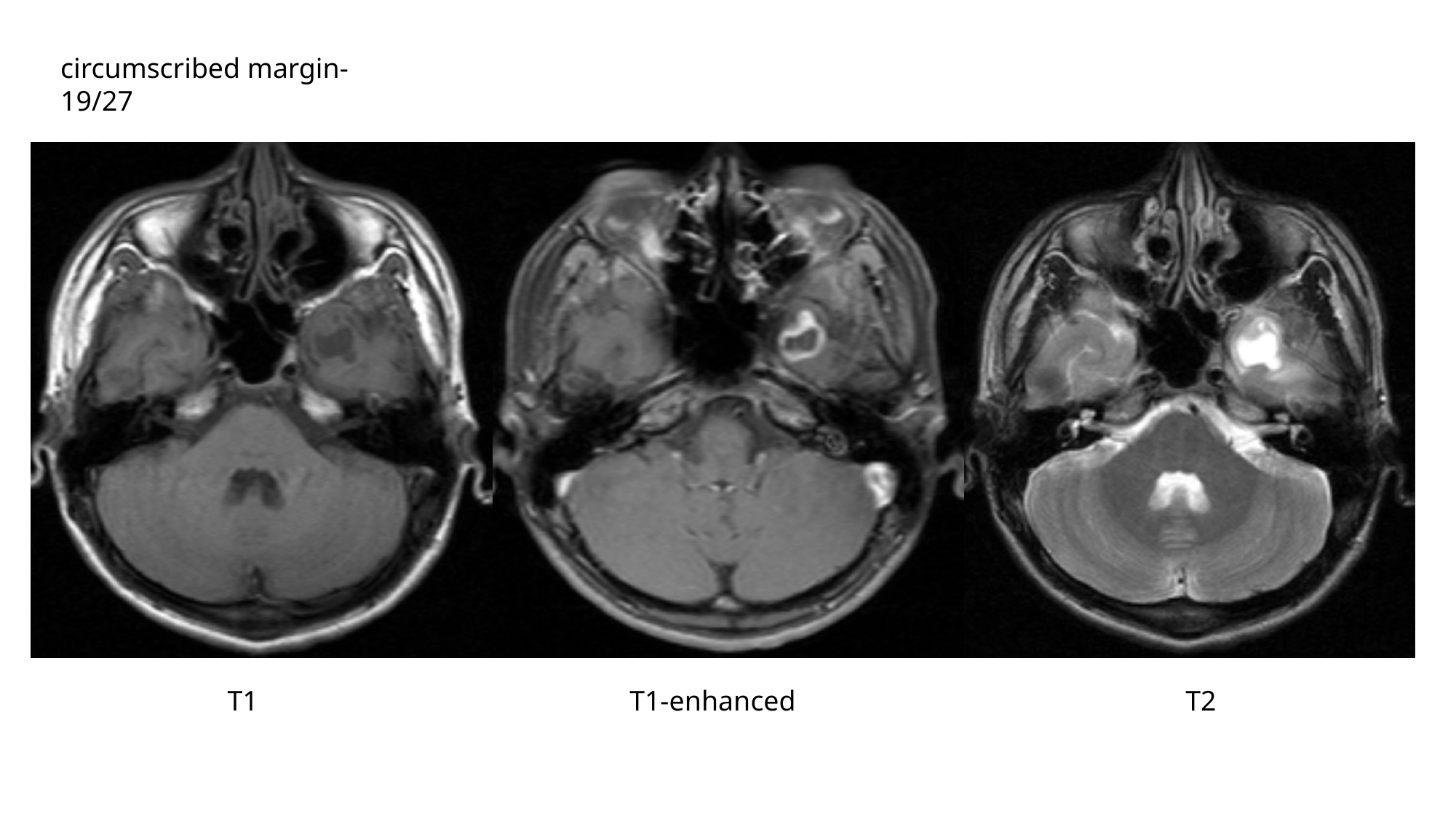

circumscribed margin-19/27
T1
T1-enhanced
T2

## Slide 42
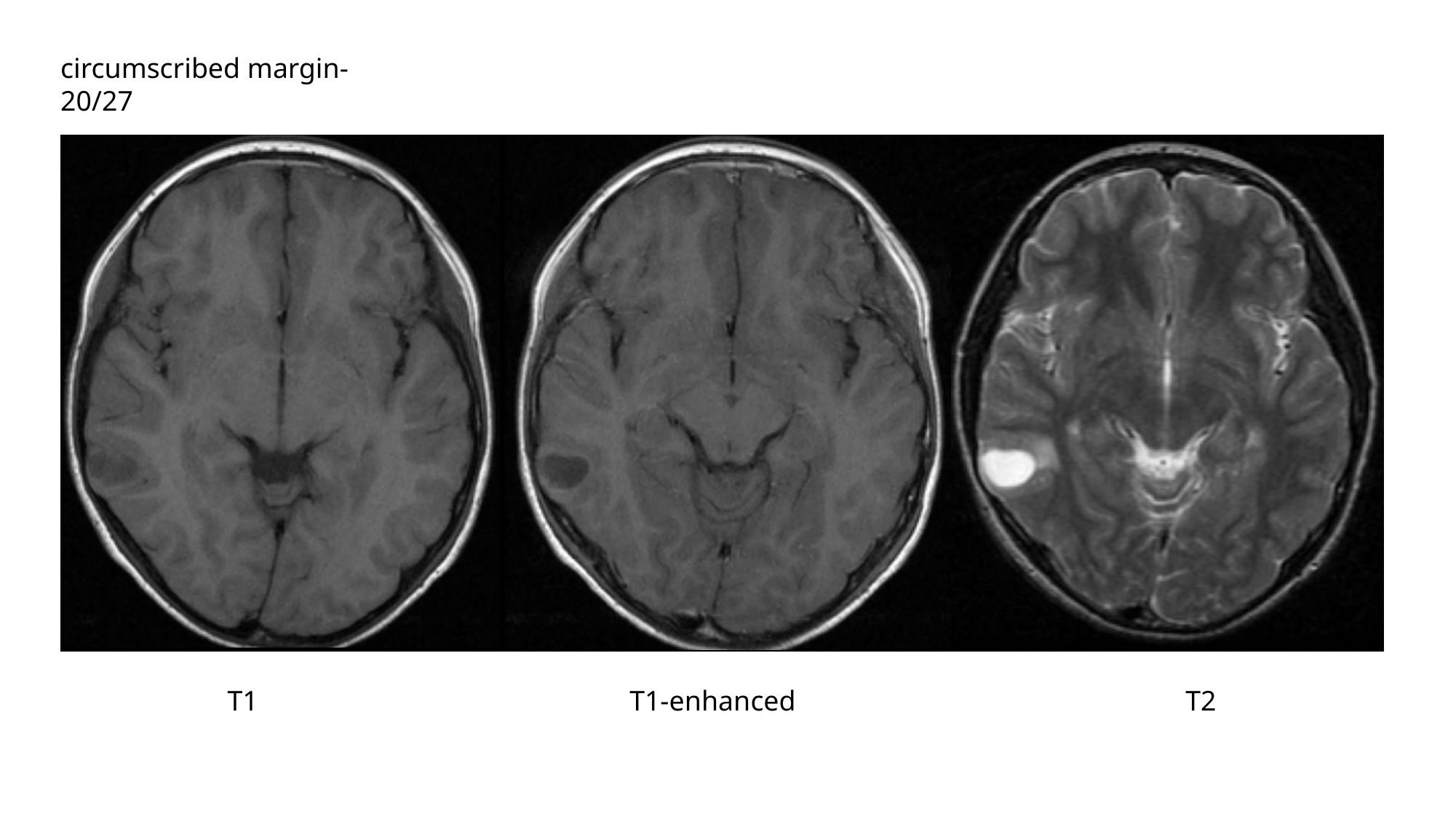

circumscribed margin-20/27
T1
T1-enhanced
T2

## Slide 43
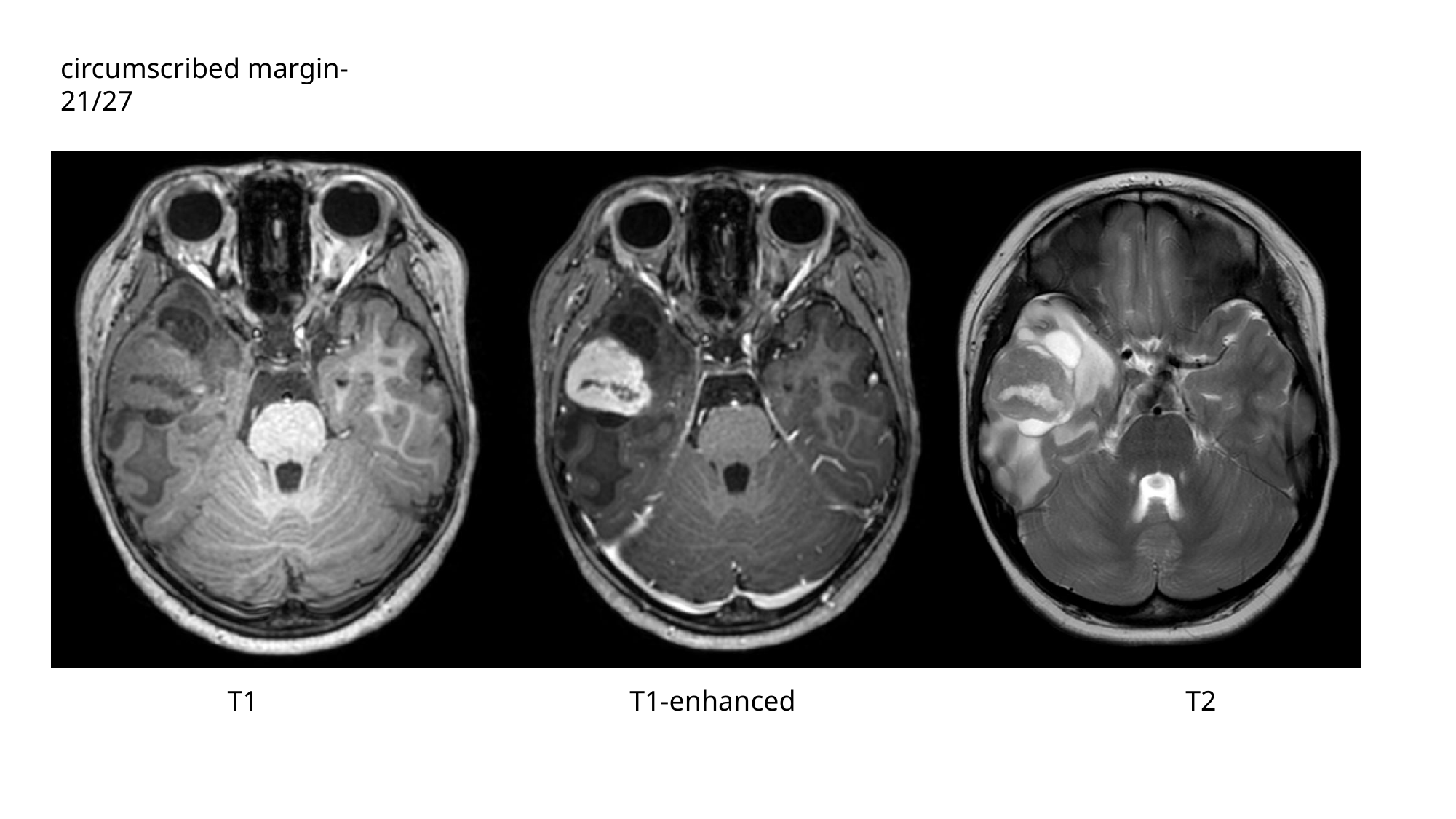

circumscribed margin-21/27
T1
T1-enhanced
T2

## Slide 44
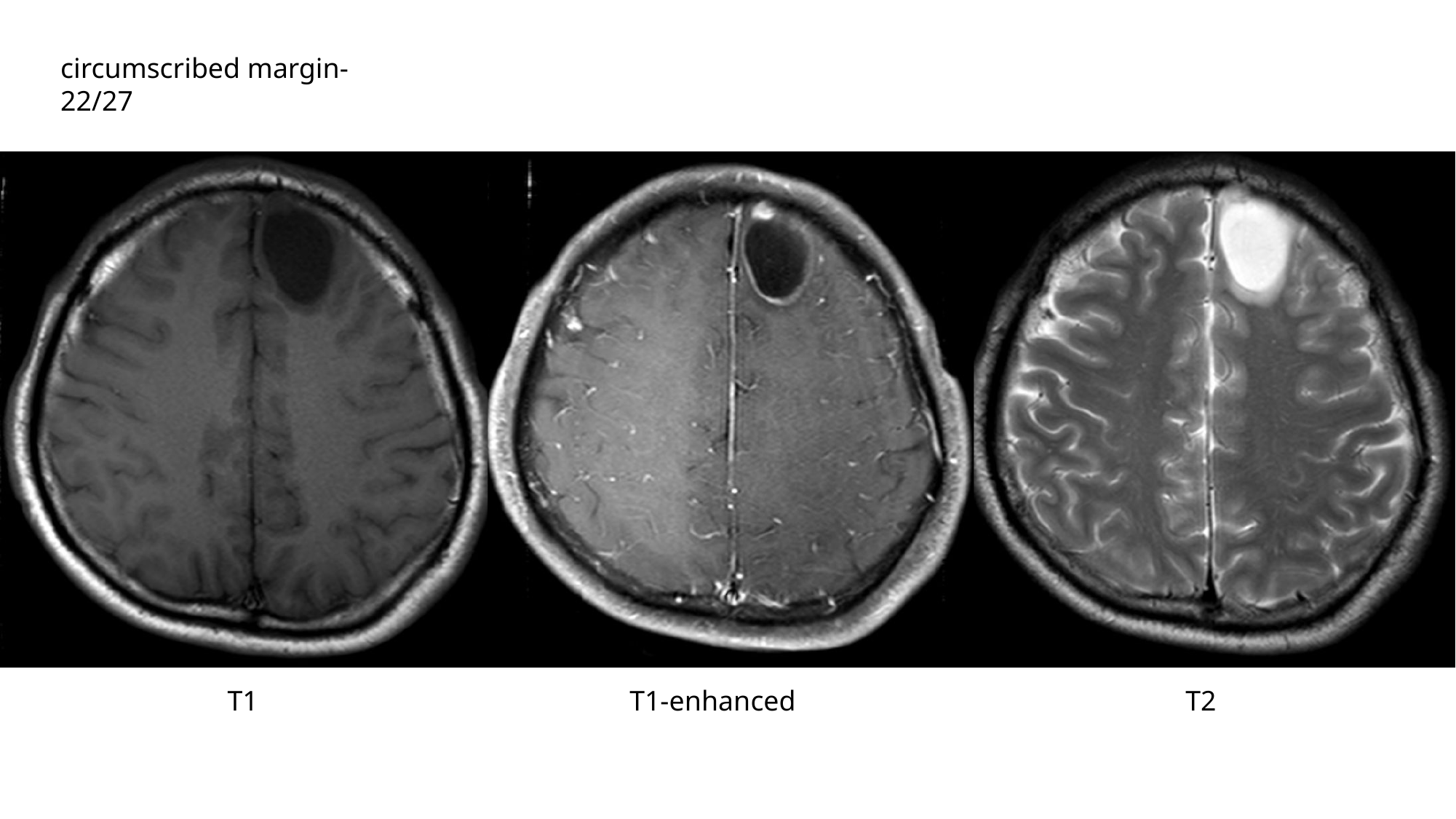

circumscribed margin-22/27
T1
T1-enhanced
T2

## Slide 45
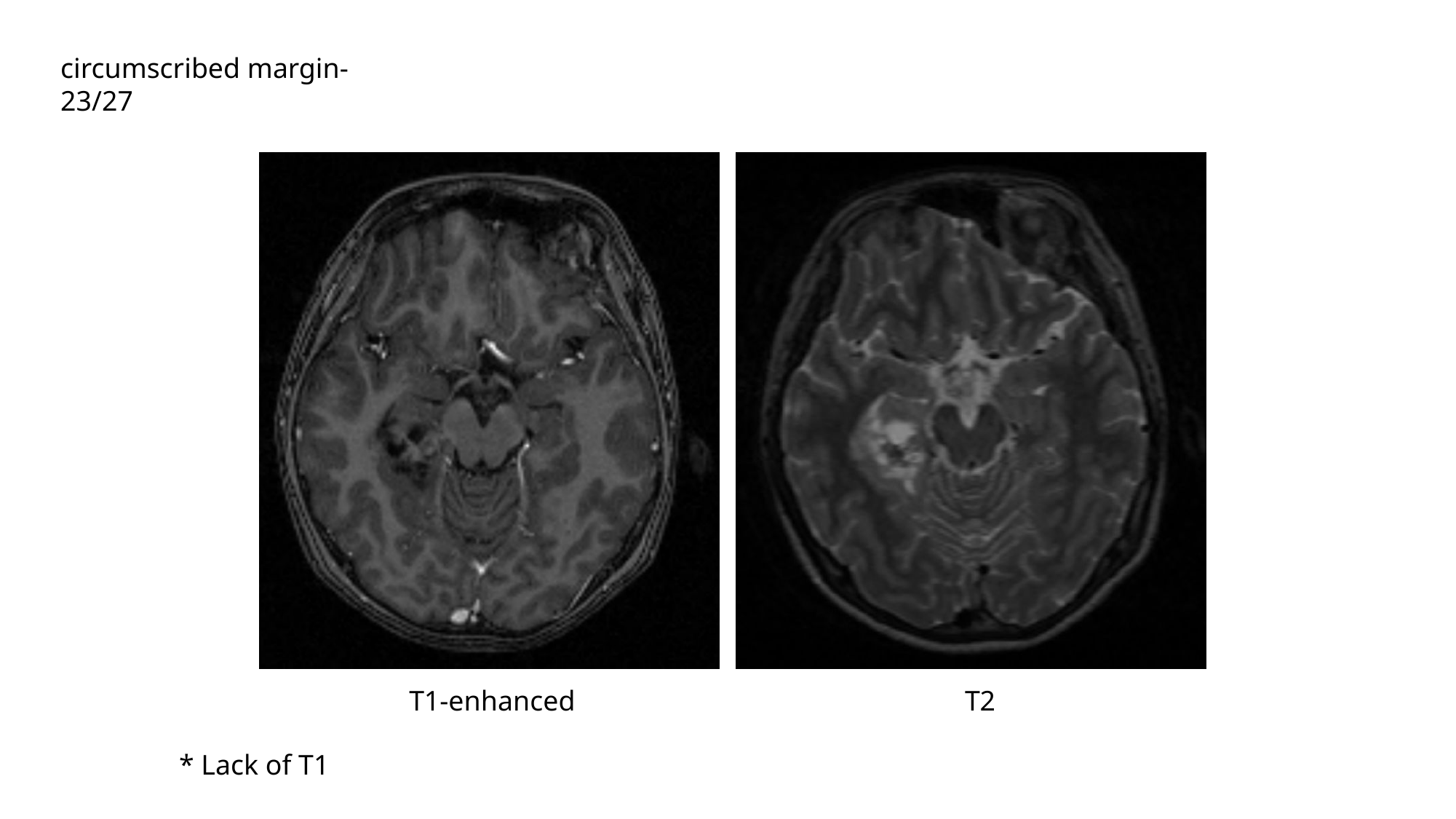

circumscribed margin-23/27
T1-enhanced
T2
* Lack of T1

## Slide 46
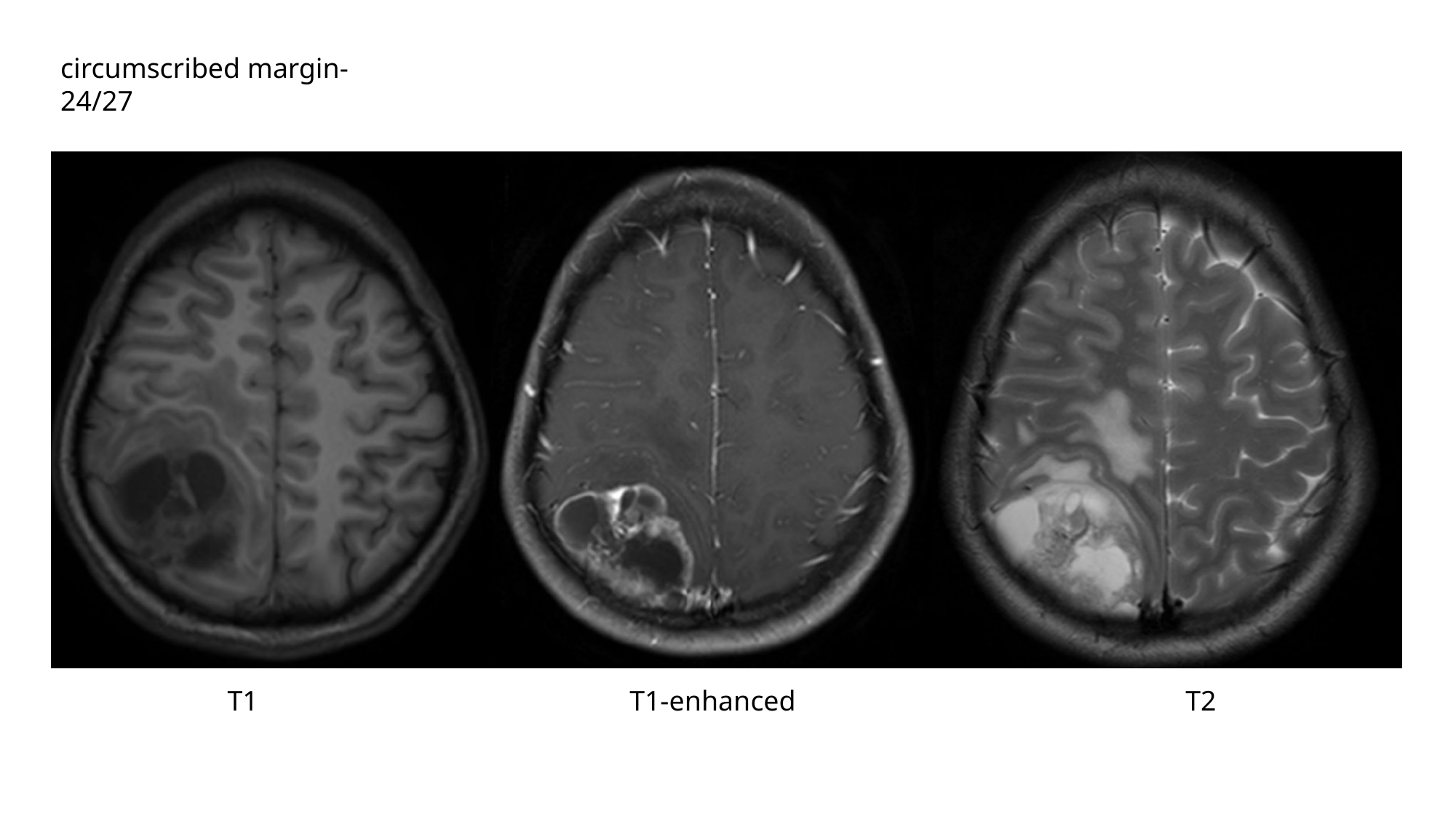

circumscribed margin-24/27
T1
T1-enhanced
T2

## Slide 47
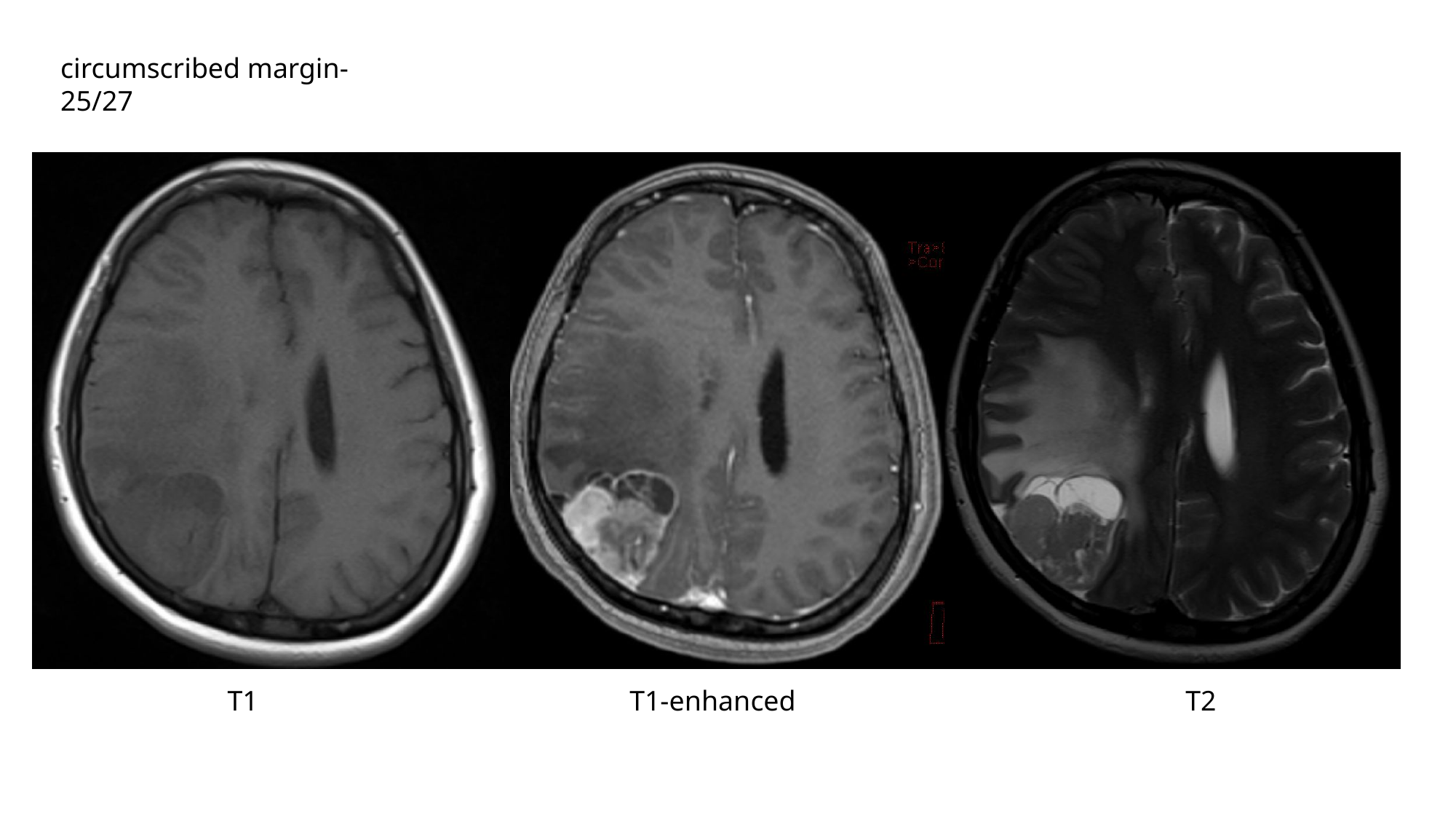

circumscribed margin-25/27
T1
T1-enhanced
T2

## Slide 48
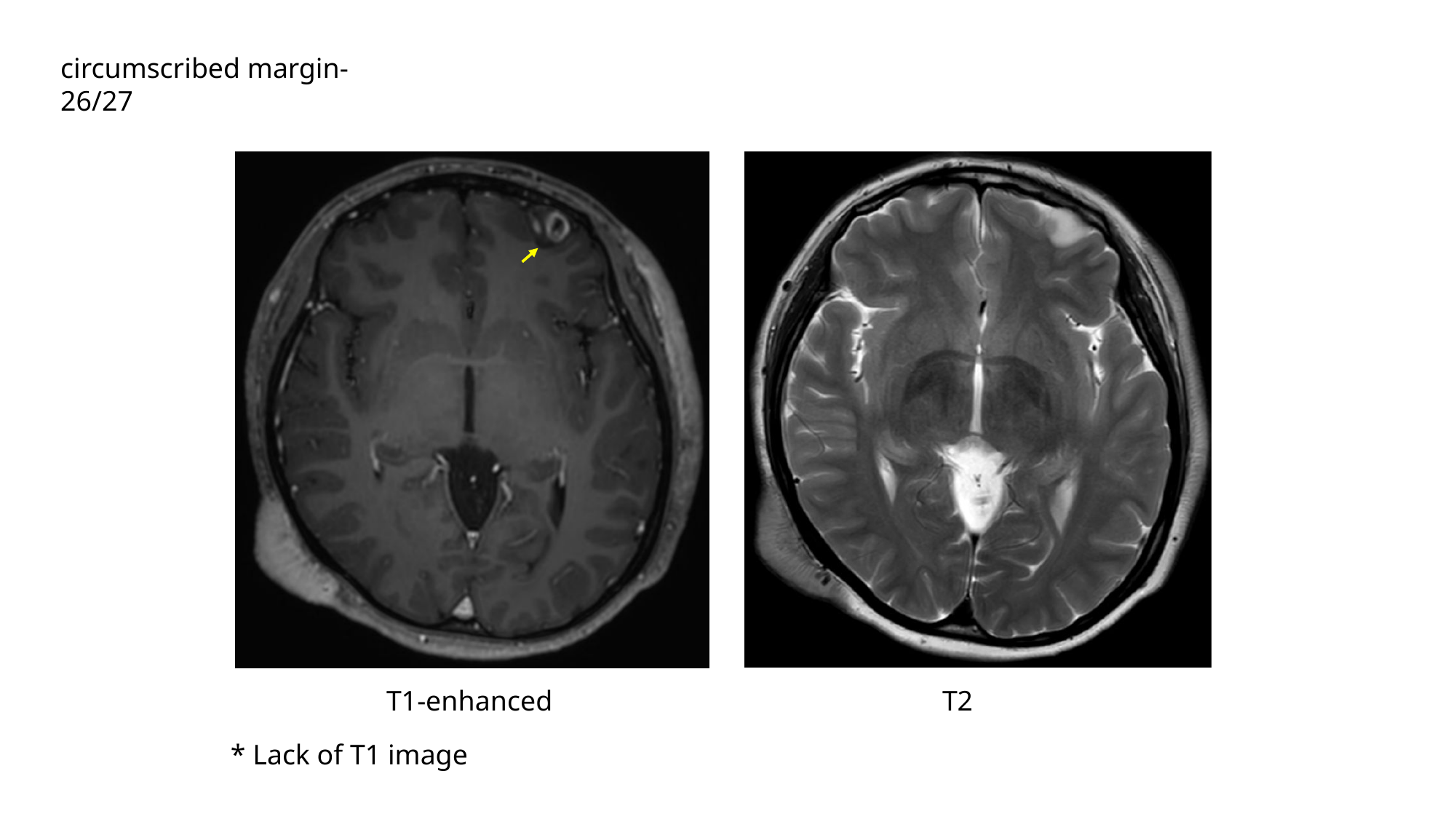

circumscribed margin-26/27
T1-enhanced
T2
* Lack of T1 image

## Slide 49
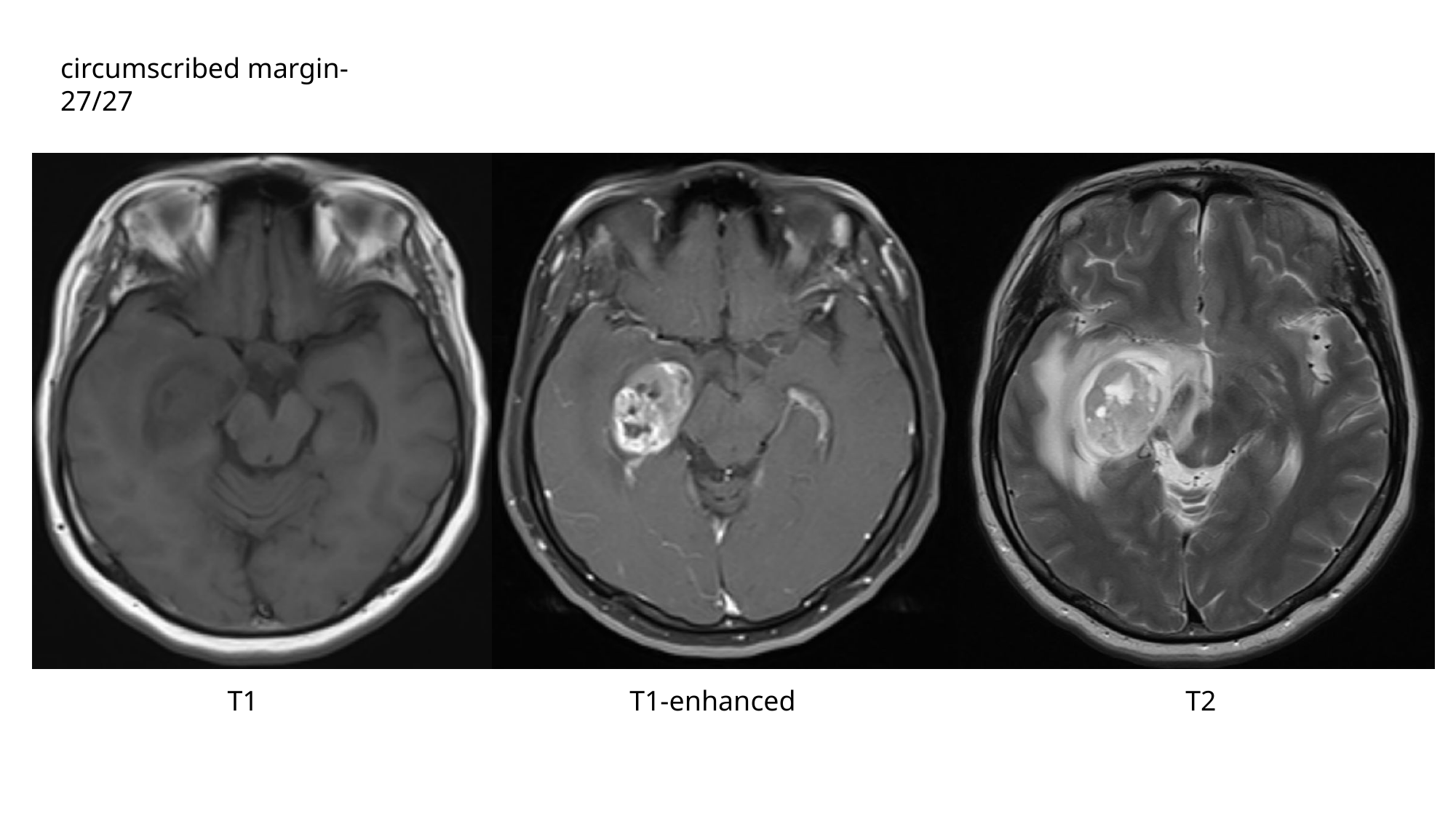

circumscribed margin-27/27
T1
T1-enhanced
T2
