# Supplementary material for: Exploring prognostic factors and treatment strategies for long-term survival in pleomorphic xanthoastrocytoma patients
Source: Sci Rep. 2024 Feb 26;14:4615. doi: 10.1038/s41598-024-55202-6 (PMC10897451; doi:10.1038/s41598-024-55202-6)
Supplement: Supplementary file 2 — Supplementary Table S1. [file 41598_2024_55202_MOESM2_ESM.docx]

Supplementary table 1. Univariate and multivariate analyses of the PFS in grade 2 PXA

|  | No. of recurrence | Univariate |  | Multivariate |  |  |
| --- | --- | --- | --- | --- | --- | --- |
|  | /No. of patients (%) | at 5 years | Log-rank | HR | 95% CI | *p* value |
| Overall | 11/40 (27.5) | 75.8 ± 7.1 | - |  |  |  |
| Sex |  |  |  |  |  |  |
| Male | 4/20 (23.8) | 84.4 ± 8.3 | 0.240 |  |  |  |
| Female | 7/20 (46.4) | 67.4 ± 11.0 |  |  |  |  |
| Age (years) |  |  |  |  |  |  |
| ≥30 | 5/14 (35.7) | 70.1 ± 12.6 | 0.334 |  |  |  |
| <30 | 6/26 (23.1) | 79.5 ± 8.2 |  |  |  |  |
| Location |  |  |  |  |  |  |
| Temporal | 3/17 (17.6) | 80.9 ± 10.0 | 0.379 |  |  |  |
| Non-temporal | 8/23 (34.8) | 72.8 ± 9.5 |  |  |  |  |
| Cystic component |  |  |  |  |  |  |
| Solid | 5/14 (35.7) | 70.7 ± 12.4 | 0.513 |  |  |  |
| Solid + Cystic | 5/19 (26.3) | 69.8 ± 11.4 |  |  |  |  |
| Cystic | 1/7 (14.3) | 100.0 |  |  |  |  |
| Tumor volume (cm^3^) |  |  |  |  |  |  |
| ≥50 | 3/9 (33.3) | 48.6 ± 18.7 | 0.082 | 3.463 | 0.763–15.723 | 0.108 |
| <50 | 8/31 (25.8) | 83.0 ± 6.9 |  |  |  |  |
| T1 enhancement |  |  |  |  |  |  |
| Strong | 8/22 (36.4) | 61.8 ± 10.7 | 0.101 |  |  |  |
| Weak | 3/18 (16.7) | 93.3 ± 6.4 |  |  |  |  |
| Tumor margin |  |  |  |  |  |  |
| Infiltrative | 8/17 (47.1) | 56.3 ± 12.5 | 0.007* | 4.713 | 0.794–27.985 | 0.088 |
| Circumscribed | 3/23 (13.0) | 89.7 ± 6.9 |  |  |  |  |
| Peritumoral edema |  |  |  |  |  |  |
| Minimal | 2/21 (9.5) | 94.4 ± 5.4 | 0.003* | 2.431 | 0.394–15.013 | 0.339 |
| Evident | 9/19 (47.4) | 55.8 ± 11.8 |  |  |  |  |
| EOR |  |  |  |  |  |  |
| GTR | 5/29 (17.2) | 80.2 ± 8.0 | 0.021* | 1.502 | 0.341–6.606 | 0.591 |
| STR | 6/11 (54.5) | 63.6 ± 14.5 |  |  |  |  |

* *p* <0.05

CI, confidence interval; EOR, extent of resection; GTR, gross total resection; HR, hazards ratio; PFS, progression-free survival; SE, standard error; STR, subtotal resection; WHO, World Health Organization
