# Supplementary material for: Exploring prognostic factors and treatment strategies for long-term survival in pleomorphic xanthoastrocytoma patients
Source: Sci Rep. 2024 Feb 26;14:4615. doi: 10.1038/s41598-024-55202-6 (PMC10897451; doi:10.1038/s41598-024-55202-6)
Supplement: Supplementary file 3 — Supplementary Table S2. [file 41598_2024_55202_MOESM3_ESM.docx]

Supplementary table 2. Univariate and multivariate analyses of the OS in grade 2 PXA

|  | No. of deaths | Univariate |  | Multivariate |  |  |
| --- | --- | --- | --- | --- | --- | --- |
|  | /No. of patients (%) | at 5 years ([%] ± SE) | Log-rank | HR | 95% CI | *p* value |
| Overall | 8/40 (20.0) | 91.4 ± 4.8 | - |  |  |  |
| Sex |  |  |  |  |  |  |
| Male | 1/20 (5.0) | 94.7 ± 5.1 | 0.023* | 0.516 | 0.053–5.020 | 0.569 |
| Female | 7/20 (35.0) | 79.5 ± 10.8 |  |  |  |  |
| Age (years) |  |  |  |  |  |  |
| ≥30 | 6/14 (42.9) | 81.3 ± 12.4 | 0.007* | 3.595 | 0.641–20.160 | 0.146 |
| <30 | 2/26 (7.7) | 95.8 ± 4.1 |  |  |  |  |
| Location |  |  |  |  |  |  |
| Temporal | 1/17 (5.9) | 93.3 ± 6.4 | 0.125 |  |  |  |
| Non-temporal | 7/23 (30.4) | 90.0 ± 6.8 |  |  |  |  |
| Cystic component |  |  |  |  |  |  |
| Solid | 5/14 (35.7) | 84.6 ± 10.0 | 0.223 |  |  |  |
| Solid + Cystic | 3/19 (15.8) | 92.9 ± 6.9 |  |  |  |  |
| Cystic | 0/7 (0.0) | 100.0 |  |  |  |  |
| Tumor volume (cm^3^) |  |  |  |  |  |  |
| ≥50 | 2/9 (22.2) | 70.0 ± 18.2 | 0.452 |  |  |  |
| <50 | 6/31 (3.2) | 96.7 ± 3.3 |  |  |  |  |
| T1 enhancement |  |  |  |  |  |  |
| Strong | 7/22 (31.8) | 84.0 ± 8.6 | 0.021* | 3.833 | 0.416–35.311 | 0.236 |
| Weak | 1/18 (5.6) | 100.0 |  |  |  |  |
| Tumor margin |  |  |  |  |  |  |
| Infiltrative | 7/17 (41.2) | 87.5 ± 8.3 | 0.003* | 2.784 | 0.227–34.157 | 0.423 |
| Circumscribed | 1/23 (4.3) | 94.1 ± 5.7 |  |  |  |  |
| Peritumoral edema |  |  |  |  |  |  |
| Minimal | 0/21 (0.0) | 100 | 0.001* |  |  |  |
| Evident | 8/19 (42.1) | 81.5 ± 9.8 |  |  |  |  |
| EOR |  |  |  |  |  |  |
| GTR | 3/29 (10.3) | 95.2 ± 4.6 | 0.026* | 2.111 | 0.405–11.001 | 0.375 |
| STR | 5/11 (45.5) | 81.8 ± 11.6 |  |  |  |  |

* *p* <0.05

CI, confidence interval; EOR, extent of resection; GTR, gross total resection; HR, hazards ratio; OS, overall survival; SE, standard error; STR, subtotal resection; WHO, World Health Organization
