# Supplementary material for: Exploring prognostic factors and treatment strategies for long-term survival in pleomorphic xanthoastrocytoma patients
Source: Sci Rep. 2024 Feb 26;14:4615. doi: 10.1038/s41598-024-55202-6 (PMC10897451; doi:10.1038/s41598-024-55202-6)
Supplement: Supplementary file 4 — Supplementary Table S3. [file 41598_2024_55202_MOESM4_ESM.docx]

Supplementary table 3. Univariate and analyses of the PFS and OS in grade 3 PXA

|  | No. of recurrences | Univariate (PFS) |  | No. of deaths | Univariate (OS) |  |
| --- | --- | --- | --- | --- | --- | --- |
|  | /No. of patients (%) | at 5 years ([%] ± SE) | Log-rank | /No. of patients (%) | at 5 years ([%] ± SE) | Log-rank |
| Overall | 7/9 (77.8) | ± | - | 5/9 (55.6) |  | - |
| Sex |  |  |  |  |  |  |
| Male | 1/1 (100.0) | 0.0 | 0.005* | 1/1 (100.0) | 0.0 | 0.094 |
| Female | 6/8 (75.0) | 72.9 ± 16.5 |  | 4/8 (50.0) | 72.9 ± 16.5 |  |
| Age (years) |  |  |  |  |  |  |
| ≥30 | 5/6 (83.3) | 44.4 ± 22.2 | 0.177 | 3/6 (50.0) | 62.5 ± 21.3 | 0.145 |
| <30 | 2/3 (66.7) | 33.3 ± 27.2 |  | 2/3 (66.7) | 66.7 ± 27.2 |  |
| Location |  |  |  |  |  |  |
| Temporal | 1/1 (100.0) | 0.0 | 0.884 | 1/1 (100.0) | 0.0 | 0.635 |
| Non-temporal | 6/8 (75.0) | 50.0 ± 17.7 |  | 4/8 (50.0) | 60.0 ± 18.2 |  |
| Cystic component |  |  |  |  |  |  |
| Solid | 4/5 (80.0) | 40.0 ± 21.9 | 0.328 | 3/5 (60.0) | 53.3 ± 24.8 | 0.889 |
| Solid + Cystic | 3/4 (75.0) | 37.5 ± 28.6 |  | 2/4 (50.0) | 75.0 ± 21.7 |  |
| Cystic | 0/0 (0.0) | 0.0 |  | 0/0 (0.0) | 0.0 |  |
| Tumor volume (cm^3^) |  |  |  |  |  |  |
| ≥50 | 2/2 (100.0) | 50.0 ± 35.4 | 0.122 | 2/2 (100) | 0.0 | 0.540 |
| <50 | 5/7 (71.4) | 47.6 ± 22.5 |  | 3/7 (42.9) | 71.4 ± 17.1 |  |
| T1 enhancement |  |  |  |  |  |  |
| Strong | 1/1 (100.0) | 84.0 ± 8.6 | 0.698 | 0/1 (0.0) | 100.0 | 0.257 |
| Weak | 6/8 (75.0) | 100.0 |  | 5/8 (62.5) | 60.0 ± 18.2 |  |
| Tumor margin |  |  |  |  |  |  |
| Infiltrative | 5/5 (100.0) | 20.0 ± 17.9 | 0.050* | 4/5 (80.0) | 40.0 ± 21.9 | 0.247 |
| Circumscribed | 2/4 (50.0) | 50.0 ± 35.4 |  | 1/4 (25.0) | 100.0 |  |
| Peritumoral edema |  |  |  |  |  |  |
| Minimal | 2/2 (100.0) | 50.0 ± 35.4 | 0.613 | 1/2 (50.0) | 50.0 ± 35.4 | 0.816 |
| Evident | 5/7 (71.4) | 28.6 ± 22.3 |  | 4/7 (57.1) | 68.6 ± 18.6 |  |
| EOR |  |  |  |  |  |  |
| GTR | 4/6 (66.7) | 66.7 ± 19.2 | 0.061 | 2/6 (33.3) | 83.3 ± 15.2 | 0.066 |
| STR | 3/3 (100.0) | 0.0 |  | 3/3 (100.0) | 33.3 ± 27.2 |  |

* *p* <0.05

CI, confidence interval; EOR, extent of resection; GTR, gross total resection; HR, hazards ratio; OS, overall survival; SE, standard error; STR, subtotal resection; WHO, World Health Organization
